# Low Silicon Content in Leaves and Higher Deposition in Roots Is Associated with *Lsi* Genes in *Psathyrostachys juncea*: Microscopy, Gene Expression and Genotyping Study

**DOI:** 10.3390/biom16091351

**Published:** 2026-09-16

**Authors:** Svetlana Dashkevich, Maral Utebayev, Nadezhda Filippova, Oksana Kradetskaya, Irina Chilimova, Botagoz Sharipova, Gulmira Khassanova, Marzhan Kuzbakova, Satyvaldy Jatayev, Nurgul Daniyeva, Yuri Shavrukov

**Affiliations:** 1 A.I.Barayev Research and Production Centre of Grain Farming, Shortandy 021601, Akmola Region, Kazakhstan; phytochem@yandex.ru (M.U.); filippova-nady@mail.ru (N.F.); oksana_cwr@mail.ru (O.K.); coronela@mail.ru (I.C.); 2 Biology Department, Sh.Ualikhanov University, Kokshetau 020000, Kazakhstan; oralovna82@mail.ru; 3 Institute of Agronomy and Forestry, Saken Seifullin Kazakh Agrotechnical University, Astana 010000, Kazakhstan; g.hasanova@kazatu.edu.kz (G.K.); happy.end777@mail.ru (M.K.); s.jatayev@kazatu.edu.kz (S.J.); 4 Core Facilities, Nazarbayev University, Astana 010000, Kazakhstan; nurgul.daniyeva@nu.edu.kz; 5 College of Science and Engineering, Biological Sciences, Flinders University, Adelaide, SA 5042, Australia

**Keywords:** ASQ markers, *Lsi* genes, microscopy, plant genotyping, *Psathyrostachys juncea*, sequencing, silicon content, SNP identification

## Abstract

Accessions of Russian wildrye, *Psathyrostachys juncea*, an important pasture fodder crop, were arranged in group A with high silicon content in leaves and group B with low silicon content. Scanning electron microscopy (SEM) and energy-dispersive X-ray spectroscopy (EDS) analyses revealed low Si accumulation in roots but higher deposition in leaves of plants from group A, whereas, in contrast, plants from group B showed significantly higher Si content in roots but lower in leaves. Three identified *PjLsi* genes encoding influx and efflux of silicon were differentially expressed, with higher levels in plants from group A, including *PjLsi1* and *PjLsi3* in leaves, and *PjLsi2* in roots, compared to those from group B. A working model was proposed based on differences in microscopy and gene expression analyses. Sanger sequencing revealed one SNP in the conservative gene *PjLsi1*, and 14 and 29 SNPs in the more polymorphic DNA sequences of *PjLsi2* and *PjLsi3*, respectively. Allele-specific qPCR (ASQ) markers were developed based on optimally identified SNPs and successfully used for genotyping of all three *PjLsi* genes in a germplasm collection comprising 72 *P. juncea* accessions. Highly significant differences were found between groups of genotypes in all three *PjLsi* genes using ASQ markers with corresponding Si content in the studied accessions, including those from groups A and B. The developed ASQ markers can be tested as candidates in marker-assisted selection for genotyping of hybrid breeding lines and for identification of the best *P. juncea* genotypes with minimal Si content in leaves of pasture fodder crop.

## 1. Introduction

Herbivorous pests encompass a very diverse group of invertebrates that attack plants by sucking and chewing attractive soft leaves that are rich in nutrients. During the long history of their evolution, plants have developed various defence mechanisms against these ‘unwanted guests’ [1,2,3,4].

Pasture crops are bred by humans for grazing or cutting for silage feeding of cattle and sheep (ruminant) or horses (non-ruminant mammals). Therefore, pasture and agricultural fodder crops are selected for larger and more rapid plant growth with minimal defensive barriers against grazing and feeding animals. This situation was described in the well-developed concept of the ‘growth–defence trade-off’, where a signalling system of plant hormone crosstalk regulates the reactions and strategy of plants either to grow or defend against any herbivores, including human animals [5,6,7]. Therefore, animal grazing can be considered a more specific point for better management of pasture rather than plant defence, with some published examples like restoration ecology in riparian livestock [8], increased diversity of plant species for improvement of pasture ecosystem functions [9,10], and potential breeding programs and modelling studies on forage plant species composition for animal diets in pasture [11]. Obviously, the strategies will be very different in wild plants found in natural ecological communities compared to cultivated agricultural crops in pastures [5].

The perennial grass species, Russian wildrye [*Psathyrostachys juncea* (Fisch.) Nevski] is a promising pasture crop, but it accumulates relatively high silicon in leaves, making it less attractive for grazing animals [12,13].

Silicon (Si) is one of the most well-known elements that is absorbed by plants from soil and distributed to shoots and leaves. There are many research papers reporting the defence role of Si in response to biotic [14,15], abiotic [16,17] and both types of stresses combined [18,19]. Silicon is absorbed by plant roots as soluble silicic acid that is transported and later deposited in root endodermis and epidermal cells of the leaf, shoot and inflorescence in a process known as silicification via passive or active accumulation methods [20]. In sorghum [*Sorghum bicolor* (L.) Moench], silicification was found to be higher in leaf epidermis compared to root endodermal cells, and it was associated with greater drought tolerance among two studied cultivars [21].

It is important to emphasise that higher accumulation of Si in plants was strongly associated with better and more efficient defence against herbivores [22]. Moreover, it was demonstrated that soluble silicon in the phloem was redirected from undamaged to damaged plant parts rather than from a direct increase in Si uptake in response to herbivory attack [23]. Clipping plant shoots, which simulates grazing, was reported to cause increased Si uptake both in roots and in shoots of native Australian grass *Microlaena stipoides* [24] but not in Australian kangaroo grass, *Themeda triandra*, and African grass, *Digitaria macroblephara* [25]. Additionally, it was found that silicon defences primarily target chewing insects, but the effect of Si was similar in other groups of herbivores [26]. In rare cases, for example, in the invasive tree *Ligustrum lucidum*, it was reported that silicon accumulation in leaves was not associated with herbivory damage [27].

*Poaceae* grass plants can accumulate a high concentration of silicon to be ready for potential defence against herbivores [28]. The distribution of Si in leaves can be in epidermal cell layers and in special silica cells in polymerised form, and plants can form trichomes as silicon spines and other sharp structures known as phytoliths on their leaf surface. These make leaves abrasive, non-attractive, and indigestible to herbivores [20,29]. In microscopy analysis of the grass model species, *Brachypodium distachyon* (L.) P.Beauv., silicon was reported to change leaf surface morphology, increasing the size but reducing the density of silica and prickle cells, which act as a direct defence against leaf-chewing insects [30]. In sorghum, Si was not accumulated in vacuoles of silica cells or in other parts but initiated deposition in the periphery of living cells [31].

Plants can be variable in their silicon accumulation even between genotypes within one species, as was reported for tall fescue (*Festuca arundinacea* Schreb.) with more than 30-fold differences between 0.05% and 1.57%, with a 0.7% average in all cuts [28]. However, more importantly, higher silicon accumulation can markedly increase leaf abrasiveness [29]. In rice, silicon content varied in shoots with a 2.5-fold difference between 16.5 and 42.4 mg/g silicon in Dhala and Bala accessions, respectively [32]. In soybean, silicon content in whole plants, cvs. Hikmoksorip and Williams 82, grown in hydroponics with supplements, varied 6-fold between 0.4% and 2.4% of Si [33]. Few studies were found in *Psathyrostachys juncea*, but silicate accumulation ranged between 2.08% and 1.39% in vegetative and heading stages, respectively, moderate compared to other grass species [34]. Such results were confirmed in a later report finding 1.18% Si accumulation on average in all stages of *P. juncea* plant growth [35].

Silicon uptake by plant roots is controlled by aquaporin channels, with a proposed passive movement process [36], based on NIP2, silicon anion influx genes named Low silicon, *Lsi1*, as described in rice [37,38] and reviewed in many other plant species [33,39,40,41]. The movement of Si from roots to shoots and leaves is primarily controlled by silicon efflux transporter genes, including *Lsi2*, the first member of the gene family, and the more recently discovered *OsLsi3* and *OsLsi6* in rice [42]. *Lsi2* was found and described in maize, barley, and rice [43], in pumpkin [44], in horsetail [45], and in a bioinformatics analysis of 17 species [46]. Silicon was also shown to regulate proton pumps in vacuoles, which was confirmed with enhanced expression of vacuolar H^+^-pyrophosphatase in rice [47]. Less attention has been paid to the *Lsi3* gene. In rice, *Lsi3* was shown to work in tandem with the *Lsi2* gene, located in the parenchyma tissues, and decreased the distribution of Si to the panicles in knockout mutants [42,48]. Two *Lsi3* genes, *PeLsi3-1* and *PeLsi3-2*, were identified in moso bamboo [*Phyllostachys edulis* (Carrière) J.Houz.] [49].

Genetic polymorphism is vitally important when studying the differences between genotypes, including plants of *P. juncea* with low and high silicon accumulation. Single nucleotide polymorphism (SNP) remains one of the major types of analyses in plant research [50,51]. In *P. juncea*, SNP and genetic polymorphism were studied for tiller-related traits [52,53] and seed shattering [54]. The recently published genetic map of *P. juncea* with 558,344 high-quality specific-locus amplified fragment (SLAF) [55] and the application of over a million SLAF markers in a genome-wide association study (GWAS) of seed yield–related traits [56] are important steps for molecular-genetic study of the species, but these tools are of little use in the absence of published sequences for the mapped and used markers for readers. Additionally, there is no complete genome available for any wildrye *Psathyrostachys* species, including *P. juncea*, making it very difficult for other researchers, with limited progress in further studies.

There are many methods available for plant genotyping based on SNP analysis [57,58]. In general, it is much faster to work with medium-throughput methods, for example, based on fluorescence (Förster) resonance energy transfer (FRET). Recently, allele-specific qPCR (ASQ) methods for plant genotyping have been developed that are also suitable for chickpea [59,60]. For functional analysis, however, it is important to show how gene expression is changed during ontogenesis or in response to herbivory attack [61,62]. An RT-qPCR method thus becomes a reasonable requirement for gene studies in plants, including silicon-related gene expression [63]. Based on genotyping and gene expression analysis, the favourable genotypes can be identified and selected with low accumulation of silicon and with down-regulation or unchanged expression of Si transporter genes.

Our knowledge is insufficient about the basic mechanisms controlling Si accumulation and specific *Lsi* gene expression in *P. juncea*, as well as in the practical application and breeding of the best genotypes to improve pasture feeding crops. Therefore, based on the identified SNPs, marker-assisted selection (MAS) can be tested with candidate genes [64,65]. Such a strategy is applicable for crop improvement in general [66,67], and specifically for prospective *P. juncea* genotypes with low Si accumulation for better pasture quality in Kazakhstan [68].

The aims of this study were: (1) to study genetic polymorphism and to identify accessions of Russian wildrye, *Psathyrostachys juncea*, with low and high silicon accumulation; (2) to investigate the distribution of silicon accumulation inside and on the surface of leaf and root sections in the studied accessions using scanning electron microscopy and energy-dispersive X-ray spectroscopy; (3) to analyse expression of *PjLsi* genes in roots and leaves of the studied accessions; (4) to identify SNPs based on Sanger sequences in *PjLsi* genes and develop molecular ASQ markers for genotyping of the studied accessions; and (5) to compare phenotyping and genotyping results to verify the possibility of marker-assisted selection with developed ASQ markers for silicon content in *P. juncea* accessions for pasture fodder cropping in Kazakhstan.

## 2. Materials and Methods

### 2.1. Plant Material and Growth

**Figure 1:**
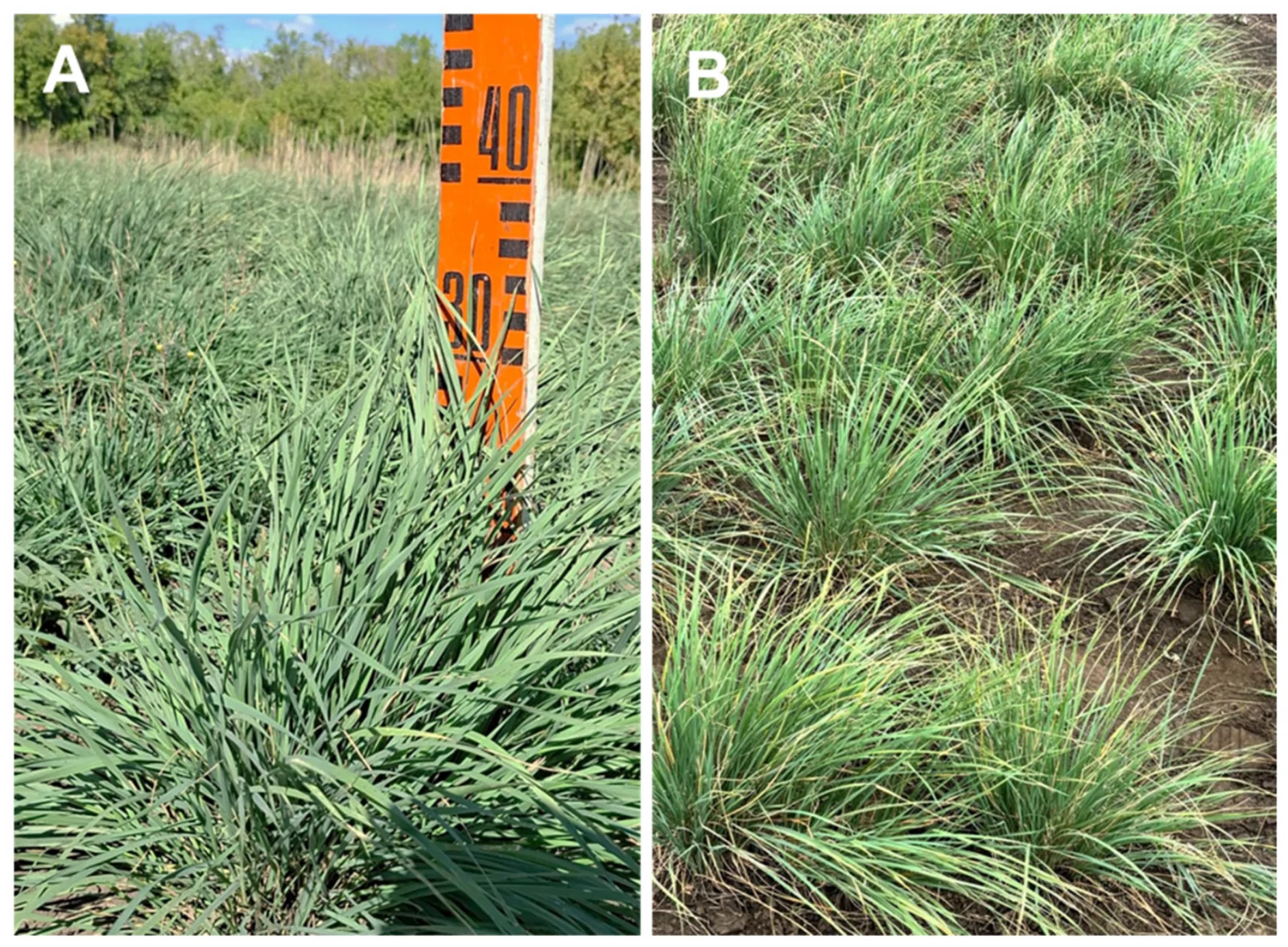
Images of *Psathyrostachys juncea* plants grown in field conditions. (**A**) Plants of standard cv. Shortandinsky. (**B**) Pasture field trial with *P. juncea* plants at the A.I.Barayev Research and Production Centre of Grain Farming, Shortandy (Kazakhstan).

In this study, 72 accessions of Russian wildrye, *Psathyrostachys juncea* (Fisch.) Nevski, were used from various origins. Seeds were received from N.I.Vavilov Research Institute of Plant Industry (VIR), St. Petersburg, Russia, in 2019. The full list of accessions and their origins is present in Supplementary material S1. Examples of *P. juncea* are shown in Figure 1, with standard cv. Shortandinsky and plants in a pasture field trial.

The field trial was carried out at the A.I.Barayev Research and Production Centre of Grain Farming (RPCGF), Shortandy (Kazakhstan), in environmental conditions as described in the previous paper [68]. The soil was carbonated chestnut chernozem with 0.3% total nitrogen, 0.1% phosphorus, and up to 5% carbonate. Slightly alkaline soil pH was recorded, ranging between 7.6 and 7.9, and with silicon content available for plants was 4.8–5.1%. Plants were grown in a research field, without any Si supplements, in single-row plots, 3 m in length and 0.6 m between rows, with a plot area of 1.8 m^2^ and a density of 35–37 plants per m^2^ in each plot. Each genotype had three replicated independent plots with a completely randomised field trial design. Experiments were carried out in 2024 and 2025, using 3-year-old plants from the same accessions grown under conditions as described in previous experiments [68]. Cutting of pasture biomass, imitating grazing, was carried out in the middle of May, 30 days after plants re-grew. At the cutting stage, plants of *P. juncea* were 40–45 cm in height and prior to the appearance of reproductive shoots.

### 2.2. Scanning Electron Microscopy (SEM) and Energy-Dispersive X-Ray Spectroscopy (EDS)

Samples for microscopy were collected from *P. juncea* plants grown in the same research field 30 days after plants re-grew, prior to cuttings. One to two young roots about 5–6 cm in length were dug from the soil alongside each randomly selected plant, cut, briefly rinsed in water to remove soil particles, blotted with paper towels, put in plastic tubes, and frozen in liquid nitrogen. About 2–3 cm sections of the youngest fully developed leaf on the top of the plant rosette were cut from the same plants and similarly frozen. Three biological replicates (individual plants), both roots and leaves, from each accession were selected for microscopy samples.

In the laboratory, three pieces about 5 mm in length were cut from each root and leaf sample, making nine samples of root and leaf for each studied genotype, which were then lyophilised using a Lyotrap Freeze Dryer (LTE Scientific, Greenfield, UK) at −50 °C and a vacuum of 0.3 mbar overnight. The lyophilised samples were coated with a 15 nm thick layer of gold using a sputter coater Quorum Q150ES (Quorum Technologies, Laughton, UK). Scanning electron microscopy (SEM) imaging and sample morphology were carried out using an Auriga Crossbeam 540 (Carl Zeiss, Oberkochen, Germany).

SEM was used in conjunction with elemental composition analysis of the samples based on energy-dispersive X-ray spectroscopy (EDS) using a detector X-Max 150 with supplementary computer software AZtecLive, ver.5.1 (Oxford Instruments, Abingdon, UK). The procedures used were similar to those published recently [17] but broadly based on methods used earlier [29,45,69]. SEM-EDS analysis was performed at an accelerating voltage of 20 kV and a working distance of 5 mm.

Silicon content was measured as weight percentages (w, %). Nine samples were studied for both roots and leaves, including three individual plants (biological replicates) and three sub-samples from each plant. Two images and corresponding Si measurements were made in each studied sub-sample and used for silicon content and distribution analyses. Results for silicon content in sub-samples were averaged, and the final result was presented as an average for biological replicates and standard errors. These analyses were carried out in the Electron Microscopy Laboratory, Core Facilities and HPC, Nazarbayev University, Astana (Kazakhstan).

### 2.3. Bioinformatics Analysis of Lsi1, Lsi2 and Lsi3 Genes and Primer Design

A search of orthologous *Lsi* genes was based on the initially published sequences in rice, *OsLsi1*, AK069842 [38], in barley, *HvLsi2*, AB447483 [43], and *BdLsi3* in *Brachypodium distachyon*, XP_003572047 [49], and was extended to bread wheat using the BLASTN, ver.1 website, Kyoto University (Japan) [70], and the Gramene website [71]. Sequences of the identified orthologs were compared using CLUSTALW, ver.1, Multiple Sequence Alignment website, Kyoto University (Japan) [72]. The identified sequences of orthologous *Lsi* genes are present in Supplementary material S2, and they were used for molecular phylogenetic tree construction using the SplitsTree4 program, ver.4.14.4 [73].

For sequence analyses and RT-qPCR gene expression of *PjLsi1*, *PjLsi2*, and *PjLsi3* in *Psathyrostachys juncea*, primers were designed based on conservative regions of the created alignments. Genetic regions with the designed primers were checked with cDNA sequences in BioProject PRJNA789128 of *P. juncea* with 150 bp Illumina reads [74], generated by Inner Mongolia Agricultural University (China), available in the NCBI database [75]. Primers were checked for absence of self-annealing, complementarity, and hairpins using the Oligo-calculator web page, Northwestern University (USA) [76]. The synthesised primers were tested, and the best-performing primers were selected. Sequences of all primers used, both for sequencing and for RT-qPCR analysis, with details and amplicon sizes of their expected products, are present in Supplementary material S3 and S4, respectively.

### 2.4. RNA Extraction, cDNA Synthesis and Gene Expression

After 30 days of re-growing plants, in mid-May, immediately prior to the first cut of biomass from well-developed plants, root and leaf samples were collected in each accession, from three typical individual plants representing three biological replicates. Roots were dug using a small shovel from soil about 20 cm from the plant and 30 cm in depth, and five young roots were taken, quickly rinsed in a beaker with clean water, and blotted with paper towel. Three-cm fragments from the root tips were then collected in plastic tubes and snap-frozen in liquid nitrogen. One leaf on the top of the plant rosette, as described in the microscopy section above, was cut, and a fragment about 5 cm in length from the middle part of the leaf was put in a plastic tube, similarly frozen in liquid nitrogen and stored at −80 °C.

Root and leaf samples were ground with two 8 mm stainless ball bearings using a Vortex mixer, keeping the samples frozen. PureZOL reagent (BioRad, Hercules, CA, USA) was used for RNA extraction following the manufacturer’s protocol. Each sample, using 2 μg of RNA, was reverse transcribed using the OT-M-MuLV-RH Reverse Transcriptase kit with supplementary DNase treatment (Biolabmix, Novosibirsk, Russia).

Samples of cDNA diluted with water (1:2) were used for both semi-quantitative RT-PCR and qPCR analyses. Pooled cDNA from all samples was used for semi-quantitative RT-PCR, and expected PCR products were checked as described earlier [77]. For qPCR analyses, gene expression was studied using a QuantStudio-7 Real-Time PCR system instrument (Thermo Fisher Scientific, Waltham, MA, USA), following the qPCR protocol published earlier [78] with some recent modifications. Briefly, the total volume (10 μL) of qPCR in each well included 5 μL of 2× Biomaster HS-qPCR SYBR Blue (Biolabmix, Novosibirsk, Russia), 3 μL of diluted cDNA, and 1 μL of two 3 μM gene-specific primers, following the manufacturer’s recommendations [79].

Thermal cycling conditions included a brief initial melt at 95 °C for 3 min, followed by 40 cycles of 95 °C for 5 s and 60 °C for 20 s, and finished with a melt curve from 60 °C to 95 °C, increasing by 0.5 °C increments every 5 s. The efficiencies of all qPCR primers were calculated based on the slope of the corresponding calibration line, E = 10^slope^, and their suitability was confirmed. Specificities of target and reference gene amplifications were verified with single distinct peaks on a melting curve and a single band of the expected size in a 2% agarose gel. The Relative Standard Dilution method was used based on the ABI Guide for relative quantitation of gene expression using real-time quantitative PCR [80], where serial dilutions (1 fmol, ×10^−2^, ×10^−4^, ×10^−6^, and sterile water for negative control) were applied for each target and reference gene individually. Threshold cycle values were determined based on linear calibration of template cDNA dilution factor and Cq value [80].

Expression data for three target genes, *PjLsi1*, *PjLsi2,* and *PjLsi3*, were calculated with normalisation of gene expression based on reference gene *PjActin1*, described earlier as very suitable for gene expression analysis [52], and this gene is an ortholog of reference gene *TaActin-2*, LOC123105253, in bread wheat [81]. The expression level of the reference gene was set to ‘1’, making it possible to compare expressions of the three studied *PjLsi* genes accordingly. Sequences, details, and expected amplicon sizes of amplified products are present in Supplementary material S4. Three biological and two technical replicates were used for samples in each tissue (roots and shoots) and in each of six studied genotypes in RT-qPCR experiments.

### 2.5. DNA Extraction and PCR for Sequencing

Fragments of about five cm of the same leaves, as described in the microscopy section above, from five individual plants in each accession of *P. juncea* were collected in 10 mL tubes separately for DNA extraction and transported in an insulated cooler with ice to the laboratory at S.Seifullin KATRU (Kazakhstan). Tubes with leaf samples were frozen in liquid nitrogen with two added ball bearings and ground with a Vortex mixer, ensuring samples remained frozen. DNA was extracted using the CTAB method at KATRU, as described earlier [82]. The washed and dried DNA pellet was finally dissolved in 100 µL of 1/10 diluted TE Buffer with 25 µg of Ambion RNase Cocktail added (Thermo Fisher Scientific, Waltham, MA, USA). The DNA concentration was measured by a NanoDrop spectrophotometer (Thermo Fisher Scientific, Waltham, MA, USA); the DNA quality was assessed on a 1% agarose gel.

For sequencing, the procedure was as published earlier [83]. PCR was performed in 60 µL reactions containing 6 µL of template DNA adjusted to 20 ng/µL, and with the following components in their final concentrations: 1× PCR buffer, 2 mM MgCl_2_, 0.2 mM each of dNTPs, 0.25 mM of each primer, and 4.0 units of GoTaq Flexi DNA polymerase (Promega, Madison, WI, USA) in each reaction. PCR was carried out on a SimpliAmp Cycler (Thermo Fisher Scientific, Waltham, MA, USA) at KATRU and on a T100 Thermal Cycler (BioRad, Hercules, CA, USA) at Flinders University, using the following program: initial denaturation, 94 °C for 2 min; 35 cycles of 94 °C for 10 s, 56 °C for 15 s, and 72 °C for 1 min, and a final extension of 72 °C for 3 min. PCR visualisation was performed using a 1% agarose gel.

The PCR products were purified using either a PCR Purification kit (Syntol, Novosibirsk, Russia) at KATRU or a FavorPrep PCR Purification kit (Favorgene Biotec, Ping Tung, Taiwan) following the corresponding manufacturer’s protocols, and their concentrations were measured using a NanoDrop spectrophotometer (Thermo Fisher Scientific, Waltham, MA, USA). Sanger sequencing was carried out using BigDye Terminator v3.1 reagents and SeqStudio, Applied Biosystems (Thermo Fisher Scientific, Waltham, MA, USA) at KATRU or as a template for Sanger sequencing at the Australian Genome Research Facility (Adelaide, Australia).

### 2.6. SNP Identification and ASQ Genotyping

SNPs were identified using manual comparison of the visualised sequences using the Chromas computer software program, version 2.0. The identified SNPs were verified by sequencing of the same PCR products in both directions.

Plant genotyping was based on the ASQ method from the original protocol [59] with the following recent modifications [79]. Briefly, the molecular probe with a short 4-bp tag was used, and two allele-specific forward primers together with one reverse primer were designed for the identified SNPs: PjLsi1-SNP1, PjLsi2-SNP4 and PjLsi3-SNP25-27. The composition of the PCR cocktail for ASQ genotyping in a total volume of 10 µL and sequences of the universal molecular probes were published earlier [83]. The design and sequence of allele-specific primers for the ASQ method in all three *PjLsi* genes are present in the Supplementary material S5.

The primers and molecular probes were obtained from DNA Synthesis (Moscow, Russia) for KATRU and purchased from Sigma-Merck (St. Louis, MI, USA) for Flinders University. Reactions were carried out in a 96-well microplate. Allele discrimination was determined using a QuantStudio-7 Real-Time PCR system (Thermo Fisher Scientific, Waltham, MA, USA) at KATRU and a CFX96 Real-Time qPCR system with CFX Maestro software 2.3 (BioRad, Hercules, CA, USA) at Flinders University, with fluorescence automatically recorded on both instruments. Amplification of FAM and HEX was checked and controlled, whereas SNP calling and genotyping results were determined in a post-run step with analysis of the Real-Time dRn setting. Genotyping experiments were carried out with three individual plants (biological replicates), and results were validated with two repeated runs (technical replicates) for each genotype and negative template controls. All sequenced genotypes of *P. juncea* in our experiment showing clear SNPs were used as reference genotypes for allele discrimination. The ASQ markers used passed control points for call quality with perfect agreement between repeated runs and absence of discordance in genotyping within each studied accession.

### 2.7. Silicon Determination

At the cutting stage, leaf biomass from all plants in each cut plot was divided into three similar parts that were treated as three sub-samples for each of the studied genotypes. Each sub-sample was dried in a 60 °C incubator over five days until completely dry before being crushed and ground in a Lab-mill Perten-3100 (Perkin Elmer, Shelton, CT, USA) with a 0.8 mm sieve. Dried and ground sub-samples were kept in a desiccator with silica gel until further biochemical analyses.

Quantitative determination of silicon compounds was carried out following a published protocol [21] with minor modifications. Dried and ground samples were ashed in ceramic crucibles (cups) at a temperature of 550–650 °C in a muffle oven. The ash was dissolved in 10 mL of a mixture of hydrochloric and nitric acids (1:3) with heating at 100 °C for 1 min and subsequent filtration using Whatman ashless filter paper. All elements were dissolved by the acids except silicon, which remained on the filter paper. The filter paper was then rinsed and ashed in clean crucibles at the same temperature of 550–650 °C and weighed after cooling for quantification of Si content in percentages of total dry biomass. The measurements were made twice (two technical repeats) in each sub-sample and averaged in each plot, as mentioned above.

### 2.8. Statistical Treatment

Excel 365 (Microsoft) and IBM software packages SPSS, version 25.0.0.0, were used to calculate and analyse means, standard errors, and significance levels using *t*-tests for microscopy and expression analyses between independent groups, and two-way ANOVA with the post-hoc Tukey test for expression analyses in accessions. Kruskal–Wallis test was employed for comparison between three groups of genotypes and their phenotyping evaluation for silicon content in leaf biomass. Three biological replicates and six sub-samples (three fragments of tissues and two images) were used for microscopy analyses, for both roots and leaves. Three biological replicates and two technical repeats were used for RT-qPCR experiments, plant genotyping, and silicon content study in leaf biomass in each accession, genotype, and experiment, respectively.

The authors declare that no generative artificial intelligence (GenAI) was used in any part of the manuscript preparation.

## 3. Results

### 3.1. Scanning Electron Microscopy (SEM) and Energy-Dispersive X-Ray Spectroscopy (EDS)

**Figure 2:**
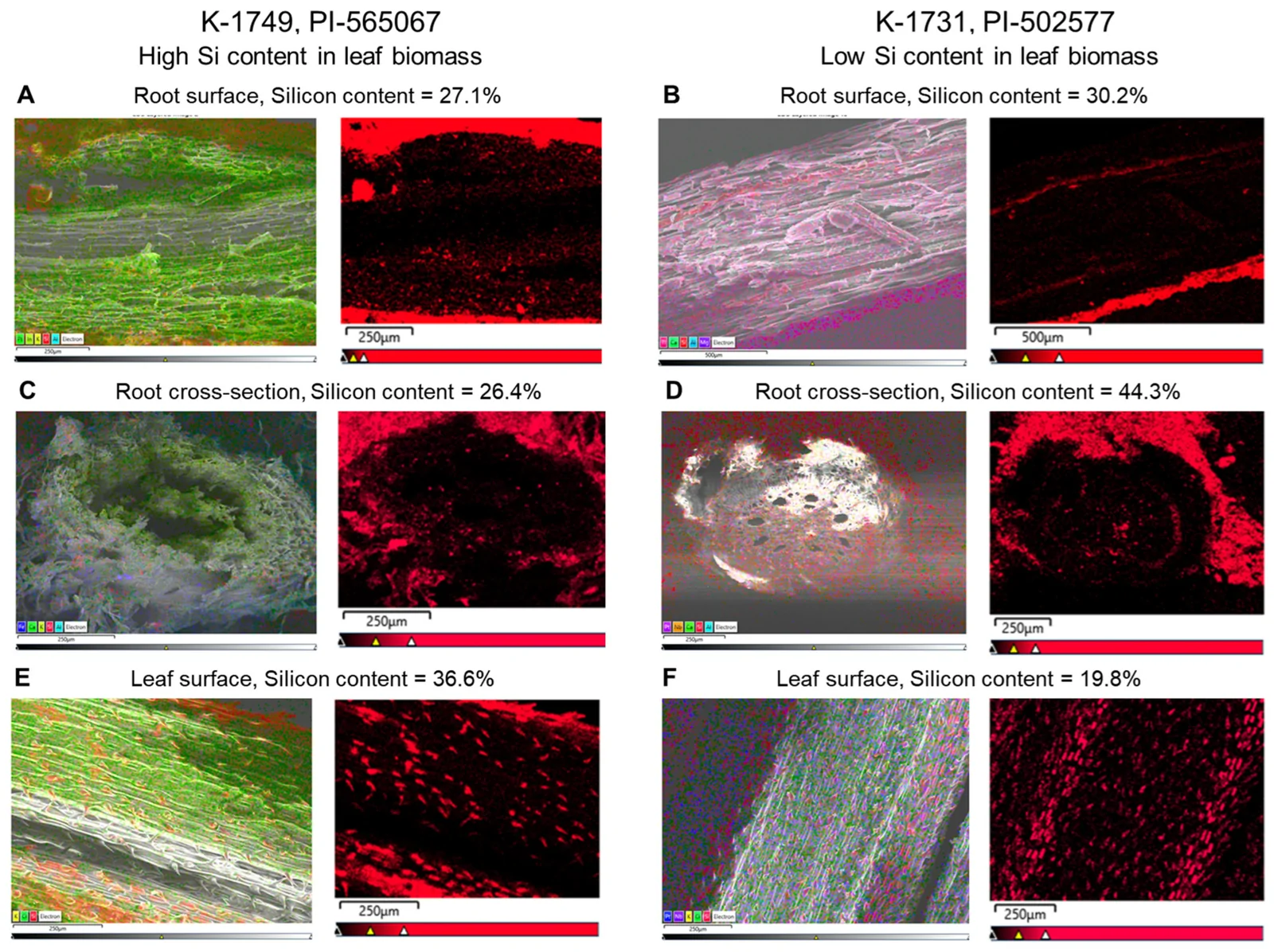
Scanning electron microscopy (SEM) and energy-dispersive X-ray spectroscopy (EDS) analysis of root surface (**A**,**B**), root cross-sections (**C**,**D**) and leaf surface (**E**,**F**) collected from plants of two *P. juncea* accessions, K-1749, PI-565067 (**A**,**C**,**E**) and K-1731, PI-502577 (**B**,**D**,**F**). Each image contains two parts. On the left-hand side, SEM images are shown, whereas on the right-hand side, the same images demonstrate the distribution of silicon only indicated in red using EDS. Each chemical element studied was designated by a different colour shown at the bottom of each image, together with a magnitude scale.

Microscopy analysis of roots and leaves in plants of *Psathyrostachys juncea* showed large differences between genotypes with high and low accumulation of silicate in leaf biomass. Examples of microscopy images of root surface and cross-section and leaf surface in two accessions with contrasting Si content are present in Figure 2. The full report containing the microscope images and quantitative spectra of Si and other elements in roots and leaves of six genotypes of *P. juncea*, three with high and three with low silicon content in leaf biomass, is present in Supplementary material S6.

The distribution of silicon in roots was mostly in the outer cell layers and much smaller in deeper root cells (Figure 2A,B), and this was consistent with root cross-sections, where exodermis and partly cortex layers are shown to contain the most silicon, whereas stele layers with xylem had very little silicon (Figure 2C,D). Accumulation of silicon was at a similar rate on the root surface, but it was 1.7-fold higher in root cross-sections of the K-1731 accession compared to those in the K-1749 accession. However, the presented results for microscopy analysis in roots were not supported by the whole-root tissue study for silicon content.

In leaves, trichomes or leaf spines in the exodermis, representing phytoliths with mostly accumulated silicon, were larger in size but lower in density in the K-1749 accession compared to the smaller in size but greater trichome numbers in the K-1731 accession. Bigger trichomes in the leaves of K-1749 plants were also associated with 1.8-fold higher Si content than those in leaves of K-1731 (Figure 2E,F).

SEM and EDS microscopy results with Si content are summarised in Table 1, where six studied *P. juncea* genotypes were arranged in two groups, A and B, three accessions in each group, with high and low silicon content in leaf biomass, respectively, based on previously published data [68]. The comparison of averaged silicon content with variations showed strongly contrasting results. Plants from group A had significantly lower Si content in roots but higher in leaves compared to those in group B. In other words, the highest level of silicon content was found in leaves of plants in group A and in roots of plants in group B (Table 1).

### 3.2. Identification of Lsi1, Lsi2 and Lsi3 Genes in Cereals and Grasses Related to P. juncea

**Figure 3:**
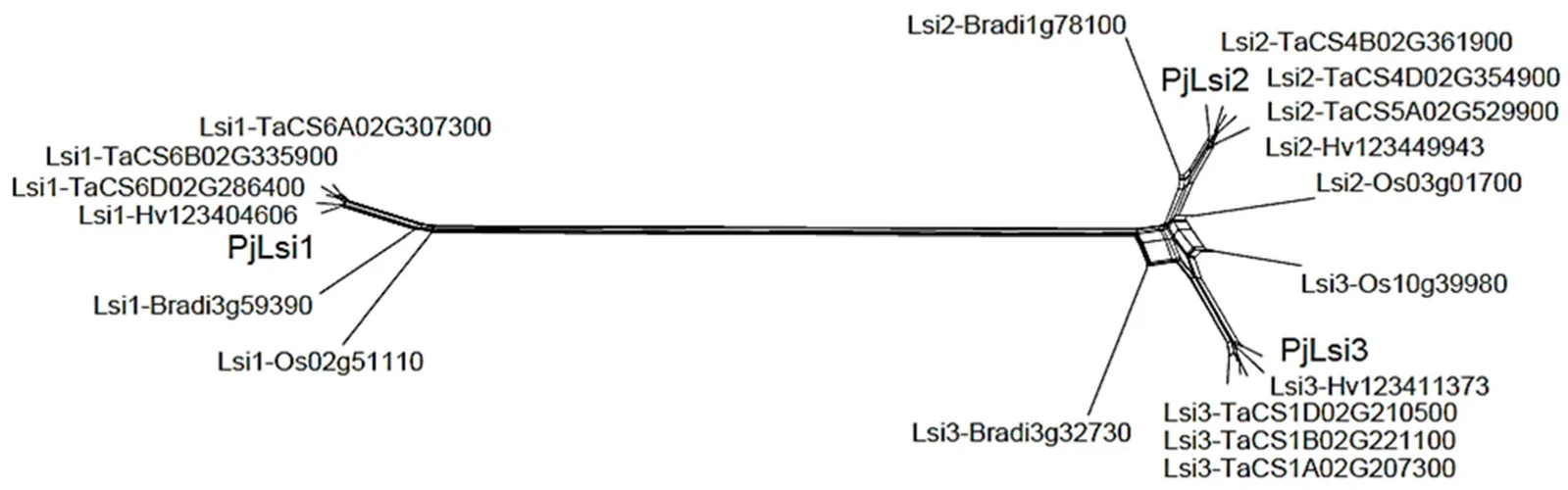
Molecular phylogenetic tree for *Lsi1*, *Lsi2* and *Lsi3* genes in bread wheat, barley, rice, *Brachypodium distachyon* and *Psathyrostachys juncea* based on sequences of coding regions. The molecular dendrogram was constructed using the SplitsTree4 program [73]. The full sequences of the analysed genes are presented in Supplementary material S2.

The groups of *Lsi1*, *Lsi2* and *Lsi3* genes identified as orthologs in rice, were *OsLsi1*, LOC_Os02g51110; *OsLsi2*, LOC_Os03g01700; and *OsLsi3*, LOC_Os10g39980. In the model plant species, *Brachypodium distachyon*, the following orthologs were found: *BdLsi1*, Bradi3g59390; *BdLsi2*, Bradi1g78100; and *BdLsi3*, Bradi3g32730. In other cereal species, barley was selected, and the orthologous genes identified were: *HvLsi1*, LOC-Hv123404606; *HvLsi2*, LOC-Hv123449943; and *HvLsi3*, LOC-Hv123411373. Finally, bread wheat was added with three homeologous genes in bread wheat, cv. Chinese Spring, including *TaLsi1*, TraesCS6A02G307300, TraesCS6B02G335900 and TraesCS6D02G286400; *TaLsi2*, TraesCS5A02G529900, TraesCS4B02G361900 and TraesCS4D02G354900; and *TaLsi3*, TraesCS1A02G207300, TraesCS1B02G221100 and TraesCS1D02G210500. These sequences are present in Supplementary material S2 and were used for molecular phylogenetic tree construction (Figure 3).

The molecular phylogenetic tree based on sequences of three genes, *Lsi1*, *Lsi2* and *Lsi3*, revealed very distant locations of influx genes *Lsi1*, whereas both efflux genes, *Lsi2* and *Lsi3*, were much closer. Genes *PjLsi* from *P. juncea* were closely related to bread wheat and barley, but much more diverse relationships were found with rice and *B. distachyon* (Figure 3).

### 3.3. RT-qPCR Expression Analysis of PjLsi Genes in Roots and Leaves of P. juncea Plants

**Figure 4:**
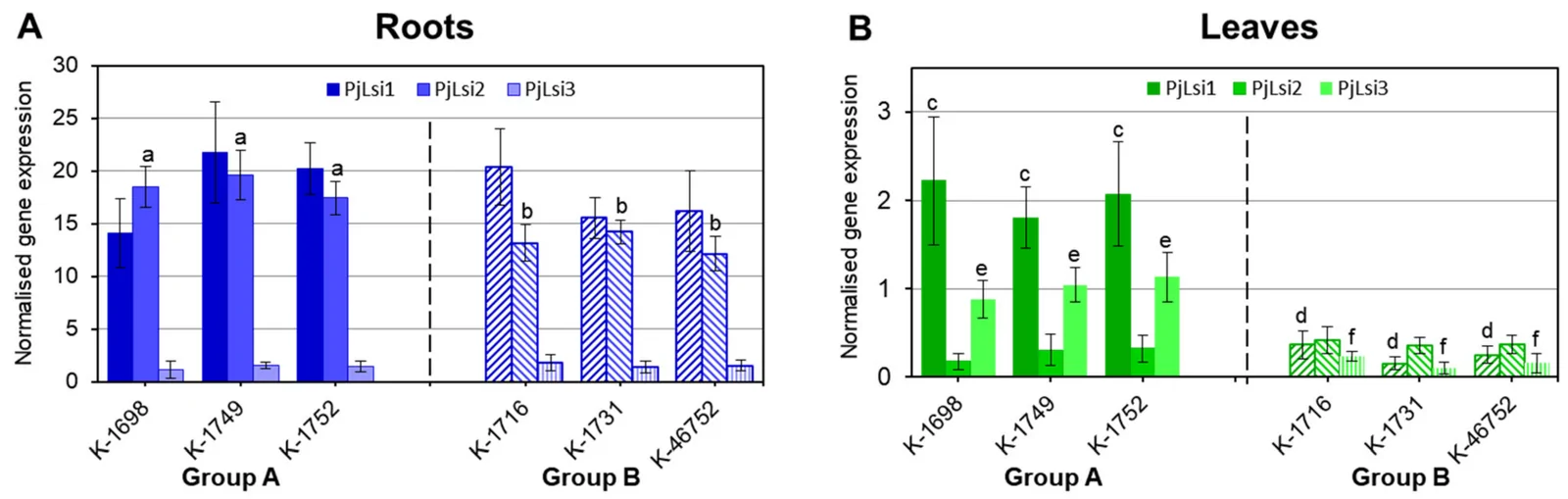
RT-qPCR analysis of *PjLsi1, PjLsi2,* and *PjLsi3* gene expression in roots and leaves of six selected accessions of *Psathyrostachys juncea* arranged in two groups (**A**,**B**), with high and low silicon content in leaf biomass. Three *PjLsi* genes were studied as indicated at the top of each panel. Expression data were normalised using the reference gene, *PjActin1* [52], and these data are presented as bars with the average ± SE of three biological replicates (individual plants) and two technical repeats for each genotype and tissue sample. Significant differences (*p* < 0.05) between genotypes for each gene were calculated using two-way ANOVA with a post hoc Tukey test and indicated by different letters. Absence of letters means no significant differences between genotypes for the studied gene.

In root samples, two genes, *PjLsi1* and *PjLsi2*, controlling Si influx and efflux into and out of cells, respectively, had very high expression levels in all six studied accessions in both groups A and B. In contrast, a significant difference was found in the expression level of the *PjLsi2* gene, averaging 18.5 and 13.2 units in groups A and B, respectively. A much smaller expression level was recorded for the third *PjLsi3* efflux gene, at around 10-fold smaller (1.2–1.8 units), with the absence of significant differences between studied genotypes from both groups, A and B (Figure 4A).

Very different results were obtained in the analyses of leaf samples (Figure 4B). In three genotypes of *P. juncea* in group A with high Si accumulation (K-1698, K1749, and K-1752), the expression of the *PjLsi1* gene varied between 1.8–2.2 units, which was almost 10-fold lower than those in roots of the same accessions. Significantly lower expression of the same gene was recorded in genotypes from group B with low Si accumulation (K-1716, K-1731 and K-46752), and the reduction was about 8-fold compared to group A and 16-fold in roots of the same accessions.

This indicated that the *PjLsi1* gene was lower but still differentially expressed in leaves of *P. juncea* accessions: Genotypes from group A with high silicon showed a significantly higher mRNA level of the *PjLsi1* gene in leaves compared to genotypes from group B accumulating low silicon. In a similar trend, a moderate level of the *PjLsi3* gene was recorded in leaves of *P. juncea* genotypes from group A with high Si content (0.9–1.1 units), but it was still significantly higher than those in leaves of accessions from group B with low Si accumulation (0.1–0.2 units). The gene *PjLsi2* showed a very low level of mRNA, similar in both groups of studied accessions (Figure 4B).

The presented results are summarised in Table 2, where average gene expression data were compared in groups A and B of *P. juncea* genotypes, respectively. They showed some variation, ranging between 14.1 and 21.7 and 12.1–19.6 units in both genes, respectively. *PjLsi1* gene expression levels were similar in the two groups of studied accessions, but with relatively large variations.

Summarised results presented in Table 2 show that there were no differences in the expression of *PjLsi1* and *PjLsi3* genes in roots between the two groups of *P. juncea* genotypes with contrasting Si accumulation. However, *PjLsi2* showed significantly higher expression in roots of plants from group A compared to group B. In leaves, significant differences in gene expression were found for two genes, *PjLsi1* and *PjLsi3*, which were 7.8- and 6.4-fold higher in group A compared to group B, respectively. Therefore, *P. juncea* plants from group A had significantly higher expression profiles of *PjLsi2* in roots, and *PjLsi1* and *PjLsi3* in leaves compared to plants from group B (Table 2).

### 3.4. Sequencing of PjLsi Genes and SNP Identification

**Figure 5:**
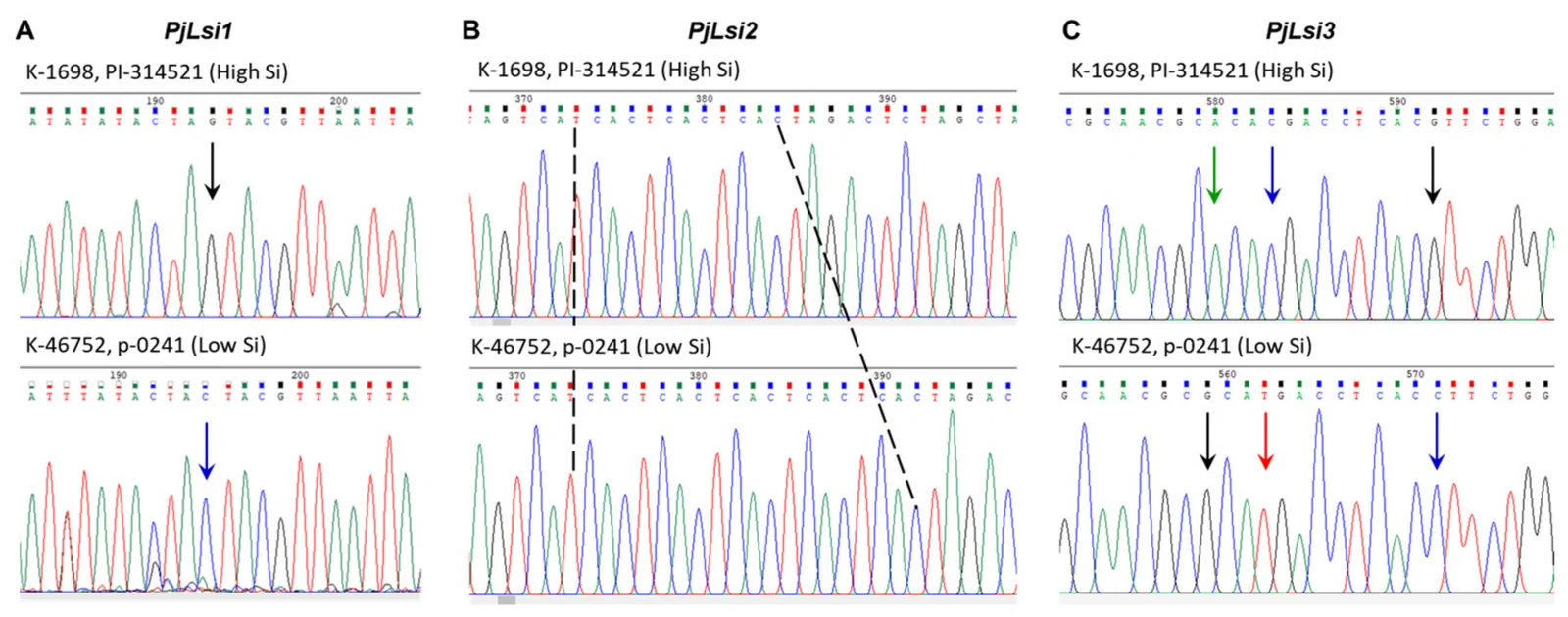
Fragments of Sanger sequencing of three *PjLsi* genes as an example of two accessions of *Psathyrostachys juncea* with high and low silicon content. (**A**) One SNP is shown in the *PjLsi1* gene. (**B**) Insertion and deletion (InDel) are present in the *PjLsi2* gene with repeated units ‘TCAC’ three and five times in accessions K-1698 and K-46752, respectively. (**C**) A cluster with three SNPs was found in the gene *PjLsi3*, demonstrating genetic polymorphism between studied genotypes. Positions of the identified SNPs are shown by arrows with corresponding colours, whereas the position of the InDel is indicated by dashed lines.

Three *Lsi* genes were sequenced in six accessions from groups A and B, studied above, for the first time in genomic DNA of *P. juncea* using Sanger sequencing with three pairs of designed primers (Supplementary material S3). Consolidated sequences of the genes are present in Supplementary material S7. Based on the sequence analysis, the first gene *PjLsi1* was the most conservative, with only one identified SNP, shown in Figure 5A as an example of two accessions of *P. juncea* with high and low accumulation of silicon.

In contrast, 14 SNPs were identified in the *PjLsi2* gene together with other genetic polymorphisms like insertions and deletions (InDel), one of which is present in Figure 5B. The most diverse was the *PjLsi3* gene with 29 various SNPs, often clustered in a small genetic fragment, representing haplotypes inherited together as one unit. The example of three clustered SNPs in *PjLsi3* is shown in Figure 5C for the same accessions of *P. juncea*.

### 3.5. Genotyping of Psathyrostachys juncea Accessions for PjLsi Genes

Genotyping was carried out using selected ASQ markers for each of the three genes, *PjLsi1*, *PjLsi2,* and *PjLsi3*, and these ASQ markers were developed based on the identified SNPs. For the first gene, *PjLsi1*, the ASQ marker PjLsi1-SNP1 was used, targeting SNP [S] with alleles [C] or [G] in intron 4, whereas ASQ marker PjLsi2-SNP4 discriminated SNP [W] with alleles [A] or [T] in intron 1 of *PjLsi2*. The most advanced ASQ markers are PjLsi3-SNP25-27, with three identified and clustered SNPs together as synonymous in exon 2: SNP [R] with alleles [A] or [G], SNP [Y] with alleles [C] or [T], and finally SNP [S] with alleles [C] or [G]. In all three ASQ markers used, the ‘*a*’ allele was associated with high Si content, whereas low silicon was related to ‘*b*’ alleles (Supplementary material S5). The identified SNPs in introns and synonymous SNPs in exons did not change amino acids in the encoded polypeptides, indicating they are only genetic polymorphisms.

**Figure 6:**
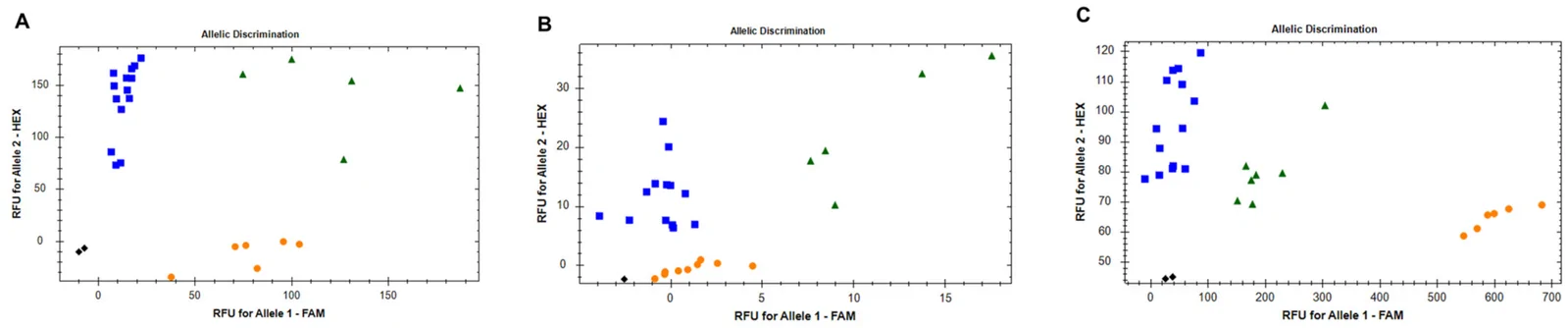
Genotyping of *Psathyrostachys juncea* accessions for three genes, *PjLsi1* (**A**), *PjLsi2* (**B**)*,* and *PjLsi3* (**C**), using the corresponding ASQ markers. Discrimination between homozygote genotypes *aa* and *bb* is shown in the plots and designated by orange dots and blue squares, respectively, whereas heterozygotes *ab* are indicated by green triangles. The fluorescence of two fluorophores, FAM and HEX, was measured in relative fluorescence units (RFU) and shown on the *X*- and *Y*-axes, respectively. The black rhombus shows the no template controls (NTC) using sterile water instead of template DNA.

An overview of the allele discrimination plots is present in Figure 6, where homozygotes *aa* and *bb* correspond to fluorophores FAM and HEX, respectively, and heterozygotes *ab* refer to both fluorophores. The genotyping results indicated that all three ASQ markers based on the identified SNPs were successful, with clear discrimination between alleles of the studied genes in all studied genotypes (Figure 6).

### 3.6. Comparison Between Genotyping of PjLsi Genes and Phenotyping of Studied Psathyrostachys juncea Accessions for Silicon Accumulation

The germplasm collection of 72 *P. juncea* accessions was tested for silicon content in leaf biomass over two years, 2024 and 2025. Data for silicon content in the two years and average values are present in Supplementary material S1, and they were grouped into three groups based on two standards, cv. Shortandinsky and cv. Bozoisky, for moderate ranks of silicon content. The first and third groups, with high and low silicon ranks, were based on previous experiments and ranged to more than 2.62% and less than 2.30% silicon content in leaf biomass, respectively. The presented data were ranked as 30 high, 26 moderate, and 16 low silicon content, respectively (Supplementary material S1).

All 72 *P. juncea* accessions were genotyped with PjLsi1-SNP1, PjLsi2-SNP4 and PjLsi3-SNP25-27 markers for three *PjLsi* genes, respectively (Supplementary material S5). The comparison of genotyping results for each *PjLsi* gene with phenotyping for silicon accumulation is shown in Table 3, with statistical treatment results of the Kruskal–Wallis test.

Kruskal–Wallis test was used to estimate statistical differences between genotype groups, presented in lines (*aa*, *ab,* and *bb*), as a ranked variable, with corresponding Si accumulation (nominal variable) in 72 studied *P. juncea* accessions. Three groups of genotypes in lines with their corresponding silicon content showed highly significant differences between each other (*p* < 0.0001), which indicates a very strong association between genotypes and phenotypes in each of the three *PjLsi* genes and silicon accumulation (Supplementary material S1). Details of the statistical analysis of the Kruskal–Wallis test are present in Supplementary material S8.

Additionally, the differences between genotyping and phenotype results are presented in Table 3 and are called ‘mismatches’, which represent two types of pairs between homozygote genotypes *aa* and heterozygotes *ab* and similarly between homozygote genotypes *bb* and heterozygotes *ab*. There were no recorded mismatches between the two homozygote genotypes, *aa* and *bb*. Therefore, all mismatches, indicated in Table 3, were present in *P. juncea* accessions with silicon content on the boundaries between both high-moderate and moderate-low silicon content but not between accessions with contrasting high and low silicon in leaf biomass.

**Table 3:** Comparison between scores of molecular markers developed for three genes, *PjLsi1*, *PjLsi2,* and *PjLsi3*, and phenotyping for silicon content in leaf biomass of 72 *Psathyrostachys juncea* accessions. Kruskal–Wallis test was used to estimate statistical differences between genotype groups with corresponding Si accumulation. Genotypes *aa*, *ab,* and *bb*, with corresponding fluorophores, are indicated by similar colours as shown in Figure 6 above.

Fluorophore	Geno- Type	Number of Genotyped Accessions	Number of Phenotyped Accessions and Silicon Content			Kruskal–Wallis Test
			Total	Mismatches	Si Content Rank	
***PjLsi1***						
FAM	***aa***	33	30	3	High	*p* = 1.02 × 10^−13^ *p* < 0.0001
FAM + HEX	***ab***	20	26	6	Moderate	
HEX	***bb***	19	16	3	Low	
***PjLsi2***						
FAM	***aa***	33	30	3	High	*p* = 3.80 × 10^−13^ *p* < 0.0001
FAM + HEX	***ab***	19	26	7	Moderate	
HEX	***bb***	20	16	4	Low	
***PjLsi3***						
FAM	***aa***	32	30	2	High	*p* = 1.42 × 10^−13^ *p* < 0.0001
FAM + HEX	***ab***	21	26	5	Moderate	
HEX	***bb***	19	16	3	Low	

**Figure 7:**
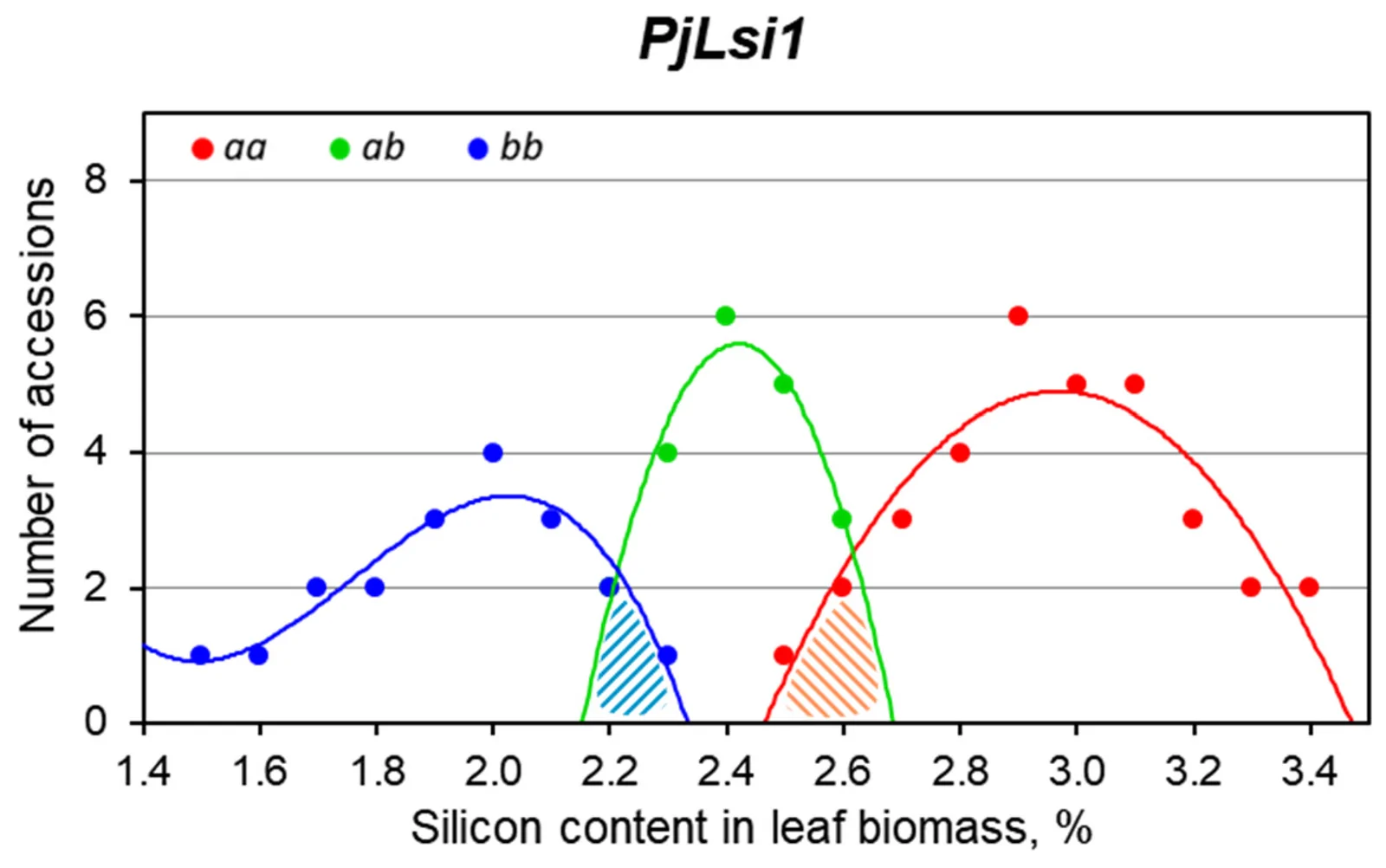
Distribution of 72 *P. juncea* accessions based on their silicon content in leaf biomass and identified with genotypes of the *PjLsi1* gene. Overlapped regions of genotype distribution highlighted by strips represent areas with mismatches in the genotyping. Genotypes *aa*, *ab* and *bb*, with corresponding fluorophores, are indicated by similar colours as shown in Figure 6 and Table 3 above.

This point is illustrated in Figure 7, showing a distribution of 72 *P. juncea* accessions based on their silicon content and identified with genotypes of the *PjLsi1* gene as an example.

In summary, genotyping with homozygotes using all three markers showed very promising results for their clear discrimination between plants with high and low silicon accumulation. However, a relatively small number of heterozygotes shared the genotyping with homozygotes, and more plants with moderate silicon content occurred in either homozygote class. This is reflected in the number of mismatches, whereas it is well known that phenotyping of silicon content can be very variable depending on environmental conditions, but genotyping results are much more stable (Table 3 and Figure 7).

## 4. Discussion

One of the most effective anti-herbivory mechanisms in plants is the accumulation of silicon and, in particular, its distribution in the epidermis and exodermis of leaves and shoots. This process of silicification protects plants from both chewing insects and from grazing mammals [1,2,3,4]. However, therein lies a conflict for agricultural pasture management. High Si content is preferable for pest resistance, but low or as minimum as possible Si in leaf biomass is required for food crop development for animal grazing and silage cuttings in pasture. Therefore, breeding strategies of crops for anti-herbivory by insects and for animal grazing in pasture are developing in absolutely opposite directions, for highest and lowest silicification and Si accumulation in leaves [5,6,7].

The very promising pasture crop, Russian wildrye (*Psathyrostachys juncea*) [84,85], is characterised by relatively high accumulation of silicon. In the current study, a germplasm collection of *P. juncea* with diverse Si content in leaf biomass was used, ranging from 1.45 to 4.11% Si [68], which was much wider than published earlier [34] but slightly above the data in another report [35]. Despite the practical interest in only minimal accumulation of silicon, genotypes with both low and high Si content were used for the current study.

### 4.1. Microscopy Analysis and Gene Expression

Results of microscopy analysis of six accessions in the current study indicated that silicon is mostly accumulated in root exodermis and cortex layers but not in endodermis, stele layers, and xylem, whereas in leaves, Si was distributed mostly in trichomes and leaf exodermis (Figure 2). However, the presented results for microscopy analysis in roots have a limitation based on the local fragments studied. The presented results were similar to those published about microscopy analysis and Si deposition on the leaf surface of grass species [21,29,86] and different deposition in roots [41,87,88].

Genetic control of influx and efflux is well known, and in the current study, three genes, *PjLsi1*, *PjLsi2,* and *PjLsi3*, were identified and analysed in six *P. juncea* genotypes. The influx *PjLsi1* gene was strongly expressed in roots of all six studied accessions, but the expression level of the same gene was 8–16-fold smaller in leaves of the same *P. juncea* genotypes from groups A and B, respectively (Figure 4). These results are in perfect consensus with those published earlier, where very strong expression of the *Lsi1* gene was reported in roots but very weak or no expression was recorded in leaves of rice [38,39], bread wheat [89], ryegrass [90], moso bamboo [49], and other species [40,41]. However, the expression profile in rice from the Genevestigator database also showed a moderate level of *OsLsi1* gene expression in a wide range of tissues, including leaf, shoot, and panicle [46]. In soybean, two isoforms of the *GmLsi1* gene were expressed in both root and shoot tissues [33], whereas the *PeLsi1* gene showed moderate expression in short and long stems of moso bamboo plants [49]. The latter results can indicate relatively wide variability in *OsLsi1*, *GmLsi1,* and *PeLsi1* gene expression in various tissues, including leaves, whereas expression of the *Lsi1* gene consistently showed the highest level in roots of all studied plant species.

The efflux *PjLsi2* gene in the current study also had very high expression levels in roots of the six studied *P. juncea* accessions, similar to the *PjLsi1* gene, and was very weak and almost undetectable in leaves of the same genotypes (Figure 4). Very similar results were reported earlier for *Lsi2* gene expression in rice [39,46,91], maize and barley [43], and in moso bamboo plants [49]. However, expression levels of two isoforms of *CmLsi2* in pumpkin and *EaLsi2* in horsetail, in leaves and shoots, were even significantly higher than in roots [44,45]. Additionally, semi-quantitative PCR in the uppermost node of rice plants at the heading stage also showed high expression of the *OsLsi2* gene [42]. These results differ from the results presented for *P. juncea* genotypes in the current study.

The efflux *Lsi3* gene is the closest homolog of *Lsi2*, and based on the presented study, they both work together in a complementary manner. In the current study, expression of *PjLsi3* was very low in roots of all six studied *P. juncea* accessions (Figure 4). The presented results were similar to those in rice published earlier, where *OsLsi3* was shown to be mainly expressed in roots and this expression was much smaller compared to *OsLsi2* [48]. However, the results in the current study show that in leaves, the *PjLsi3* gene was expressed at a much lower level but, nevertheless, genotypes from group A had moderate and significantly higher expression compared to leaf samples from group B (Figure 4). These results also have some similarity with rice. For example, no expression of *OsLsi3* was reported in the leaf blade or sheath at the vegetative stage of rice plants [48], but *OsLsi3* was highly expressed in the inflorescence and in the uppermost node of mature rice plants [42,48]. Additionally, in a similar manner, *PeLsi3* expression was high in both very short and long stems of moso bamboo plants [49].

### 4.2. Working Model of Si Influx and Efflux in Psathyrostachys juncea

**Figure 8:**
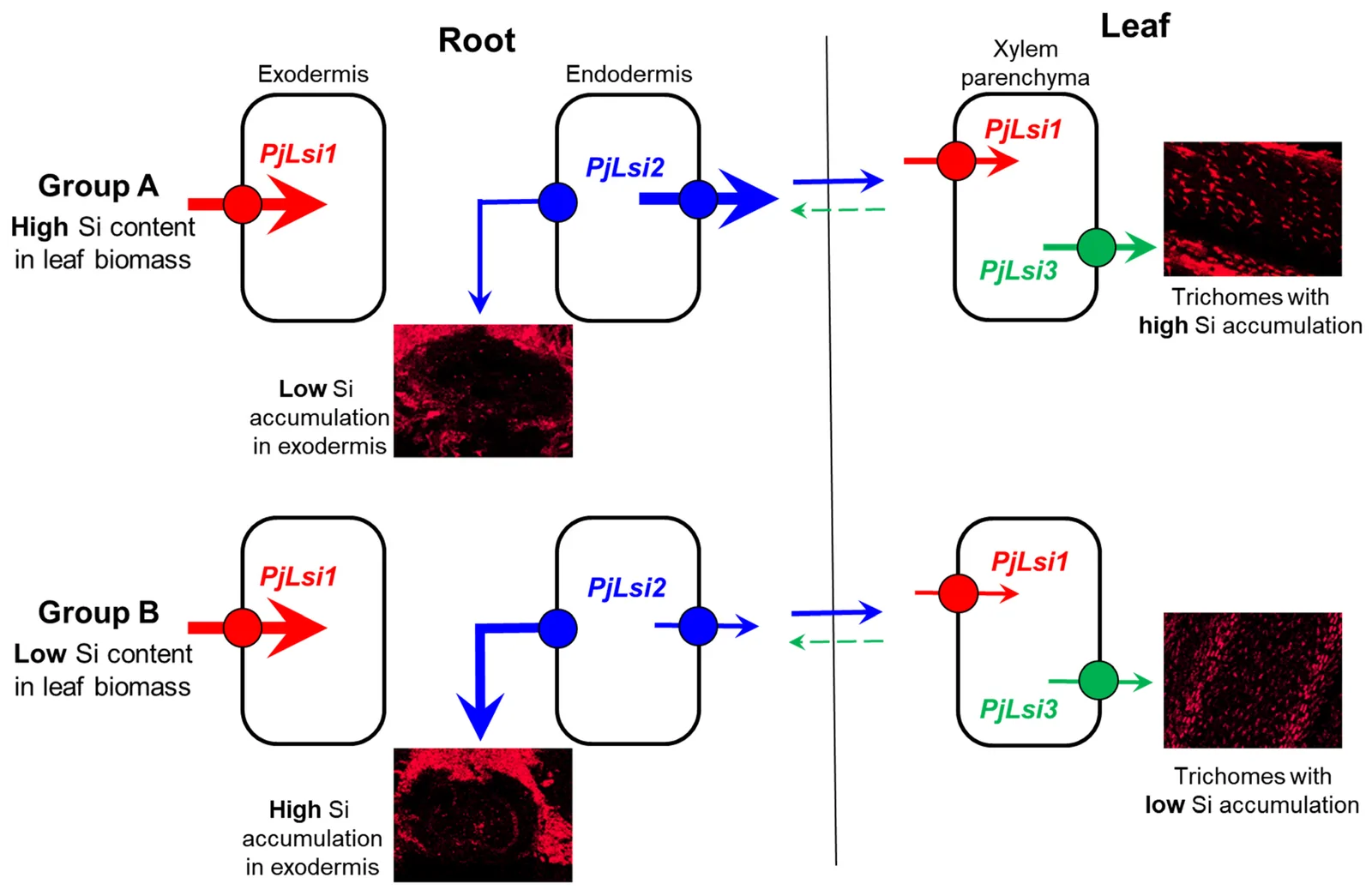
Schematic working model based on the current study presenting the expression of three genes, *PjLsi1*, *PjLsi2,* and *PjLsi3*, in roots and leaves of *Psathyrostachys juncea* plants arranged in two groups, A and B, with high and low Si content in leaves, respectively. The expression results were compared with microscopy analyses of the same samples for the distribution and content of silicon in a cross-section of roots and the surface of leaves. The size of arrows for Si influx and efflux genes *PjLsi* genes reflects their expression level in RT-qPCR analysis. The list of accessions in groups A and B is present in Table 1.

To summarise the presented results for microscopy and expression of three *PjLsi* genes in the current study, a working hypothesis was created for simpler explanation and easier comparison of influx and efflux silicon transporters in *P. juncea* plants with high and low Si content in leaf biomass from pasture cuts and in other plant species (Figure 8).

In *P. juncea*, both genes *PjLsi1* and *PjLsi2* are highly expressed in roots, possibly in both exodermis and endodermis, as shown in rice [39], but it is more likely that they have separate locations, with active expression of *PjLsi1* in the exodermis, whereas *PjLsi2* can be expressed in the endodermis of roots, as shown in maize and barley [43]. Nevertheless, in rice mutant *lsi2* and in non-mutant plants, it was reported that silicon accumulated not only in root endodermis but also in exodermal and sclerenchyma cells, and less in the outer epidermis of roots [92,93]. Similar published results indicated that silicon was deposited at its highest level in the root exodermis of onion plants [93], and in outer parenchyma cells in roots of bread wheat [94]. Therefore, we can hypothesise that *PjLsi2* can efflux Si from the root endodermis in both directions: one is to the stele and xylem with subsequent transportation to shoots and leaves, but probably more effectively and even more strongly is the ‘pushing’ of silicon back to the exodermis of roots (Figure 8).

This dual efflux of silicon in two-ways from the root endodermis has been supported by microscopy analysis results in the current study, where silicon was shown to be distributed in the epidermis and exodermis but not in internal layers of roots in six studied *P. juncea* genotypes regardless of their silicon accumulation (Figure 2 and Table 1). In the results, a portion and perhaps a large portion of silicon after influx remains in roots but, at least partially, some is relocated back to the exodermis. At the same time, another and possibly smaller portion of silicon is transported into shoots and leaves of *P. juncea* plants. It is important to emphasise that dual relocation of silicic acid either in the root exodermis or in the xylem, with upward transport to shoots and leaves, is genotype-dependent, resulting in the corresponding higher Si accumulation either in roots or in leaves, respectively (Figure 8).

This part of the working model is strongly supported by microscopy analysis, where three *P. juncea* genotypes from group A accumulate significantly less silicon in root exodermis, whereas, in contrast, a higher level of Si was found in roots of three accessions in group B (Figure 2 and Table 1). These results are similar to others published earlier, where the majority of plant species accumulated a lower level of silicon in roots and higher in shoots, for example in rice [91] and in sorghum [21]. In contrast, higher silicon was reported to accumulate in roots compared to shoots in onion plants [93]. The presented results for gene expression indicated that the level of mRNA production in *PjLsi1* in roots was similar in both groups (Figure 4 and Table 2). Therefore, it is logical to propose that low relocation of silicon to the root exodermis will be accompanied by increased Si moving to the upper part of plants into shoots and leaves. If so, it is obvious that, vice versa, plants with high accumulation of silicon in root exodermis will have reduced upstream Si flow-through into leaves (Figure 8).

There are published data with indirect support for this part of the working model. For example, in ryegrass, a negative correlation was reported between the expression level of the *LpLsi1* gene and Si accumulation in shoots [90]. However, such results may be a paradox or mistake because any up-regulation of *Lsi1* expression in roots will cause stronger influx, with a resulting higher level of Si in shoots and leaves, generating positive correlations. Only one reason can destroy this statement, and this is a dual function of efflux genes, *Lsi2* or *Lsi3* or both in some species. If high mRNA production of the *Lsi1* gene causes high influx of silicic acid in roots but also accompanying higher expression of *Lsi2* or *Lsi3* or both, and most Si is then effluxed back to the root exodermis, this process definitely results in a reduction of silicon accumulation in shoots and leaves, which is probably what happened in the case of ryegrass [90]. This point can be supported by *OsLsi3* expression levels in rice roots and the significant negative correlation shown for shoot Si accumulation [48]. This is only possible if silicon remains in roots, with reduced relocation to shoots and higher efflux after increased expression of *OsLsi3*. As shown in rice, double layers of Casparian strips and coordinated work of both *OsLsi2* and *OsLsi3* can potentially control the loading of silicic acid from root endodermis to xylem and its transportation to shoots and leaves [48].

In leaves, the expression results of the studied *PjLsi* genes in our work showed a differential pattern in six *P. juncea* genotypes from groups A and B, with high and low accumulation of silicate in leaf biomass, respectively. In group A, higher expressions of both influx *PjLsi1* and efflux *PjLsi3* means more Si coming to leaf cells, whereas lower expression levels of the same genes in group B are related to less Si deposition in leaf cells. This then results in trichomes with significantly high and low silicon quantification by microscopy analysis in groups A and B, respectively (Figure 2 and Table 1).

The described process of interaction between the three studied *PjLsi* genes in roots and leaves of six *P. juncea* genotypes is very important. Once silicic acid reaches shoots or leaves, it polymerises into trichomes and other phytoliths with solid amorphous silica. Silicon polymerisation can occur rapidly, with deposition of insoluble Si in the final stage. However, a small portion of non-polymerised silicic acid or remobilised silicon can be transported back downstream from shoots to roots via the phloem, driven by sink-source gradients. For example, substantial levels of soluble Si were present in the phloem exudate in a report finding that silicon was redirected from undamaged to damaged leaves via the phloem in wheat [23]. Such remobilisation of silicic acid from shoots to roots via the phloem is possible but, at this stage, there is no clear evidence for this and, therefore, it is indicated by dashed arrows in Figure 8.

The presented working model shows the very general processes of influx and efflux of silicon in roots and leaves of plants in six *P. juncea* accessions, where not all steps are clarified. However, it can be used to address why some *P. juncea* genotypes accumulate high or low silicon content in groups A and B, and relate it directly to the expression activity and interactions between influx and efflux Si transporter genes.

### 4.3. Sanger Sequencing, Genetic Polymorphism and SNP Identification

Despite hundreds of published papers about *Lsi* genes in various plant species, sequences or natural genetic polymorphisms were released in only a few cases. In our research, all three genes, *PjLsi1*, *PjLsi2,* and *PjLsi3*, were sequenced in six accessions from groups A and B with contrasting silicon content in leaf biomass, and the consolidated sequences are present in Supplementary material S7. This task was very challenging in the absence of a publicly available full genome sequence of *Psathyrostachys juncea*. The sequence of the *PjLsi1* gene was very conservative and revealed only one SNP in intron regions (Figure 5A). In rice, 20 SNPs were reported in *OsLsi1,* including a 10Kb interval on both sides of the ‘start’ and ‘stop’ codons of the gene. Despite the advanced technology of the high-density SNP chip used in the study, none of these SNPs were associated with genotypes with high or low silicon accumulation in 50 accessions of rice from five diverse populations [32].

The results in the current study showed 14 SNPs and one InDel identified within the *PjLsi2* gene, indicating high variability in intron regions (Figure 5B). These results were in consensus with rice, where 10 SNPs were reported in *OsLsi2,* including one synonymous SNP in the first exon. However, only two SNPs in the 2.5Kb and 6.5Kb upstream 5′-untranslated region (UTR) were associated with silicon content in the studied rice genotypes [32].

More diverse genetic polymorphism, with 29 SNPs clustered in haplotypes, was found in the *PjLsi3* gene in the current study (Figure 5C). Very similar results were reported in rice, with 20 SNPs in the *OsLsi3* gene with 10Kb intervals in the upstream and downstream UTRs. The genetic polymorphism in rice included four SNPs within exons and six SNPs within introns, whereas only one non-synonymous SNP was detected in the first exon with a rare allele and was associated with silicon content in rice accessions [32].

In summary, the studied *PjLsi* genes were very different, with the most conservative being the *PjLsi1* gene with just one detected SNP, but much more variability found in *PjLsi2* and especially in *PjLsi3,* with a high level of genetic polymorphism in the studied *P. juncea* genotypes. All these results were very similar to those in rice [32].

### 4.4. Plant Genotyping for PjLsi Genes and Their Comparison with Phenotyping

In our work, SNP-based molecular markers were developed and tested for genotyping of the germplasm collections for *PjLsi1*, *PjLsi2*, and *PjLsi3* genes (Figure 6). During the comparison of genotyping results with ASQ markers, PjLsi1-SNP1, PjLsi2-SNP4, and PjLsi3-SNP25-27, and phenotyping results for silicon content in leaf biomass, strongly significant differences were found between three groups of genotypes (*aa*, *ab,* and *bb*) in all 72 *P. juncea* accessions (Table 3). Therefore, accessions from rank 3 with low silicon content (Supplementary material S1) and genotypes *bb* in the studied *PjLsi* genes represent promising germplasm for further analysis of hybrid populations and development of prospective breeding lines.

The nature of mismatches in plant genotyping was shown in Figure 7. In comparison with rice, where a promising SNP associated with high and low accumulation of silicon in shoots [32], very similar results are present in the current study. Two molecular markers, lsi2-b and lsi2-c, were developed for the *Lsi2* gene in rice, indicating a non-synonymous mutation causing Ser-115-Asp substitution and changing OsLsi2 protein structure and activity [91]. However, no other molecular markers were developed and tested in rice and other plant species, which required additional analyses to make further comparison with the results presented in the current study.

The studied germplasm collection of *Psathyrostachys juncea,* together with the produced hybrids, was studied earlier not only for silicon content but also for various traits of nutrient content, which are important for pasture forage crops. It provides a validation of the studied genotypes suitable for further use in breeding of *P. juncea* [68].

Molecular markers developed and used in the current study can be tested as candidates for marker-assisted selection (MAS), for example in progenies from hybrid populations. In the results of MAS, the best breeding lines can be identified with associations for low silicon content in leaf biomass of *P. juncea* accessions to improve the quality of this pasture fodder crop. Future research is still required to support stronger functional validation of the studied *PjLsi* genes and their analyses in segregating populations, including molecular marker validation and application in MAS, subject to sufficient funds.

## 5. Conclusions

(1) Based on analyses of scanning electron microscopy (SEM) and energy-dispersive X-ray spectroscopy (EDS), plants of *Psathyrostachys juncea* were arranged in two groups. Group A consists of plants with low Si accumulation in roots but higher deposition in leaves, whereas plants from group B showed significantly higher Si content in roots but lower in leaves.
(2) Three identified influx and efflux *PjLsi* genes were differentially expressed, with higher levels in plants from group A, including *PjLsi1* and *PjLsi3* in leaves, and *PjLsi2* in roots, compared to those from group B.
(3) A working model was proposed based on differences in microscopy and gene expression analyses.
(4) Allele-specific qPCR (ASQ) markers were developed based on optimally identified SNPs and successfully used for genotyping of all three *PjLsi* genes in a germplasm collection comprising 72 *P. juncea* accessions.
(5) Sanger sequencing revealed one SNP in the conservative gene *PjLsi1*, and 14 and 29 SNPs in the more polymorphic coding DNA sequences of *PjLsi2* and *PjLsi3*, respectively.
(6) Highly significant differences were found between three groups of genotypes (*aa*, *ab* and *bb*) in all three *PjLsi* genes using ASQ markers and Si content in the studied accessions.
(7) The developed ASQ markers can be tested as candidates in marker-assisted selection for genotyping of hybrid breeding lines and for identification of the best *P. juncea* genotypes with minimal Si content in leaves of pasture fodder crops.

## Figures and Tables

**Table 1:** Summary results of SEM-EDS microscopy analysis of silicon content in roots (surface and cross-sections) and in leaves of plants from two groups of *P. juncea* with contrasting Si accumulation. Results are presented as averages for three biological replicates ± standard errors. Significant differences in silicon content between groups of genotypes, calculated using a *t*-test, are designated by asterisks, *, *p* < 0.05; **, *p* < 0.01.

Accession	Silicon Accumulation in Tissue Studied, w %		
	Root Surface	Root Cross-Section	Leaf Surface
**Group A.** High Si content in leaf biomass			
K-1698, PI-314521	19.5 ± 0.5	20.2 ± 1.0	32.5 ± 1.2
K-1749, PI-565067	27.1 ± 1.1	26.4 ± 1.2	36.6 ± 1.6
K-1752, PI-565073	23.2 ± 0.9	29.7 ± 1.5	31.3 ± 1.3
**Average**	23.3 ± 0.7	25.4 ± 1.3	33.5 ± 1.4
**Group B.** Low Si content in leaf biomass			
K-1716, PI-430875	33.6 ± 1.4	37.4 ± 1.8	21.4 ± 0.7
K-1731, PI-502577	30.2 ± 1.2	44.3 ± 2.2	19.8 ± 0.4
K-46752, p-0241	36.3 ± 1.7	41.5 ± 2.0	23.9 ± 1.0
**Average**	33.4 ± 1.5	41.1 ± 2.1	21.7 ± 0.8
***t*** **-test between groups**	* *p* = 0.0230 < 0.05	* *p* = 0.0104 < 0.05	** *p* = 0.0042 < 0.01

**Table 2:** Summary results for *PjLsi1*, *PjLsi2,* and *PjLsi3* normalised gene expression units in roots and leaves for the comparison between two groups of *Psathyrostachys juncea* genotypes with high (K-1698, K-1749 and K-1752) and low (K-1716, K-1731 and K-46752) accumulation of silicon, shown in Figure 4. Significant differences in gene expression between groups of genotypes calculated using a *t*-test are designated by asterisks *, *p* < 0.05, or ‘n.s.’, non-significant. SE, Standard error.

Gene Studied	Normalised Gene Expression Units				*t*-Test Between Groups
	Group A High Si Accumulation in Leaf Biomass		Group B Low Si Accumulation in Leaf Biomass		
	Average	SE	Average	SE	
**Roots**					
*PjLsi1*	18.68	3.50	17.36	3.13	n.s.
*PjLsi2*	18.51 *	1.98	13.17 *	1.52	* *p* = 0.042 < 0.05
*PjLsi3*	1.37	0.54	1.58	0.62	n.s.
**Leaves**					
*PjLsi1*	2.03 *	0.95	0.46 *	0.11	* *p* = 0.012 < 0.05
*PjLsi2*	0.27	0.07	0.38	0.12	n.s.
*PjLsi3*	1.02 *	0.28	0.20 *	0.09	* *p* = 0.015 < 0.05

## Data Availability

The original data presented in this study are included in the research paper and in the Supplementary Material. Further inquiries can be directed to the corresponding authors.

## Supplementary Materials

The following supporting information can be downloaded at https://www.mdpi.com/article/10.3390/biom16091351/s1. Supplementary material S1: List of 72 used accessions of *Psathyrostachys juncea* used in this study. Supplementary material S2: Sequences of studied CDS regions of *Lsi1*, *Lsi2,* and *Lsi3* genes in rice, *Brachypodium distachyon*, barley, bread wheat, and *Psathyrostachys juncea* used for molecular phylogeny, and alignments between bread wheat, barley, and *P. juncea* for designing primers for sequencing. Supplementary material S3: Details of primers used in this study for sequencing and amplicon size of the produced PCR products based on genomic DNA of bread wheat. Supplementary material S4: Details of primers used for RT-qPCR analysis of gene expression in *Psathyrostachys juncea*, with the following indication of the position in the consolidated CDS sequences. Supplementary material S5: Details of allele-specific primers used for ASQ genotyping of three genes, *PjLsi,* in *Psathyrostachys juncea.* Supplementary material S6: Microscopy analysis in *Psathyrostachys juncea.* Supplementary material S7: Sequences of studied genomic regions of *Lsi1*, *Lsi2* and *Lsi3* genes in *Psathyrostachys juncea* using Sanger sequencing. Primers for sequencing are indicated in the presented sequences. Exons are indicated by different colours while introns are not highlighted by colours. Supplementary material S8: Statistical analysis of the Kruskal–Wallis test presented in Table 3.
