# Supplementary material for: Low Silicon Content in Leaves and Higher Deposition in Roots Is Associated with Lsi Genes in Psathyrostachys juncea: Microscopy, Gene Expression and Genotyping Study
Source: Biomolecules. 2026 Sep 16;16(9):1351. doi: 10.3390/biom16091351 (PMC13604245; doi:10.3390/biom16091351)
Supplement: Supplementary file 1 [file biomolecules-16-01351-s001.zip › Supplementary material S2-S8.pdf]

Supplementary materials to the manuscript

Dashkevich S., Utebayev M., Filippova N., Kradetskaya O., Chilimova I., Sharipova B., Khassanova G., Kuzbakova M., Jatayev S., Daniyeva N., Shavrukov Y.

Low silicon content in leaves and higher deposition in roots is associated with lsi genes in *Psathyrostachys juncea*: Microscopy, gene expression and genotyping study

Content

<u>Supplementary material S1:</u> List of 72 used accessions of *Psathyrostachys juncea* used in this study with primary data for silicon accumulation during two years and scores of genotyping results using potential candidate ASQ markers for *PjLsi* genes. – **In separate Excel-file**

<u>Supplementary material S2:</u> Sequences of studied CDS regions of *Lsi1*, *Lsi2* and *Lsi3* genes in rice, *Brachypodium distachyon*, barley, bread wheat, and *Psathyrostachys juncea* used for molecular phylogeny, and alignments between bread wheat, barley and *P. juncea* for designing of primers for sequencing – **Pages 2-20**

<u>Supplementary material S3:</u> Details of primers used in this study for sequencing and amplicon size of the produced PCR products based on genomic DNA of bread wheat – – **Page 21**

<u>Supplementary material S4:</u> Details of primers used for RT-qPCR analysis of gene expression in *Psathyrostachys juncea*, with following indication of the position in the consolidated CDS sequences – **Pages 22-23**

<u>Supplementary material S5:</u> Details of allele-specific primers used for ASQ genotyping of three genes *PjLsi* in *Psathyrostachys juncea* – **Pages 24-25**

<u>Supplementary material S6</u>: Microscopy analysis in *Psathyrostachys juncea* – **Pages 26-43**

<u>Supplementary material S7:</u> Sequences of studied genomic regions of *Lsi1*, *Lsi2* and *Lsi3* genes in *Psathyrostachys juncea* using Sanger sequencing. Primers for sequencing are indicated in the presented sequences. Exons are indicated by different colours while introns did not highlighted by colours – **Pages 44-48**

<u>Supplementary material S8</u>: Statistical analysis of Kruskal–Wallis test presented in Table 3 – **Pages 49-51**

Supplementary material S2

Sequences of studied CDS regions of *Lsi1*, *Lsi2* and *Lsi3* genes in rice, *Brachypodium distachyon*, barley, bread wheat, and *Psathyrostachys juncea* used for molecular phylogeny, and alignments between bread wheat, barley and *P. juncea* for designing of primers for sequencing.

Lsi1

In CDS sequences, 'Start'- and 'Stop'-codons are highlighted by green and red, respectively. Exons are indicated by different colours on example of *P. juncea* sequence.

>Lsi1-Os02g51110
AGAAATGGCCAGCAACAACTCGAGAACAAACTCCAGGGCGAACTACTCCAACGAGATCCA
CGATCTCTCCACGGTGCAGAACGGCACCATGCCTACCATGTACTACGGCGAGAAGGCCAT
CGCCGACTTCTTCCCTCCTCACCTCCTCAAGAAGGTCGTGTCGGAGGTGGTGGCCACGTT
CCTGCTGGTGTTCATGACGTGTGGGGCGGCAGGGATCAGCGGCAGCGACCTGTCTCGCAT
ATCGCAGCTGGGACAGTCGATCGCCGGTGGCCTCATCGTGACGGTGATGATCTACGCCGT
CGGCCACATCTCCGGCGCCCACATGAACCCCGCCGTGACGCTCGCGTTCGCCGTGTTCAG
GCATTTCCCCTGGATTCAGGTTCCGTTCTACTGGGCGGCGCAGTTCACCGGAGCGATATG
CGCGTCGTTCGTGCTCAAGGCGGTGATCCACCCGGTGGATGTGATCGGAACCACCACGCC
CGTGGGGCCGCACTGGCACTCGCTCGTCGTCGAGGTCATCGTGACGTTCAACATGATGTT
CGTCACGCTCGCCGTCGCCACGGACACGAGAGCGGTGGGTGAGTTGGCCGGGTTGGCGGT
TGGTTCCGCGGTTTGCATTACGTCCATCTTCGCAGGGGCAATTTCAGGTGGATCGATGAA
CCCGGCAAGGACGCTGGGGCCGGCGCTGGCGAGCAACAAGTTCGACGGCCTGTGGATCTA
CTTCCTGGGCCCAGTCATGGGCACGCTCTCGGGAGCATGGACCTACACCTTCATCCGCTT
CGAGGACACCCCCAAGGAAGGCTCCTCCCAGAAGCTCTCCTCCTTCAAGCTGCGCCGCTT
GCGGAGCCAGCAGTCCATCGCCGCCGACGACGTCGACGAGATGGAGAACATCCAAGTGTG
ATAGG

>Lsi1-Bradi3g59390
CAAAATGTCTACAAACTCGAGATCCAACTCGAGGGCCAACTTCTCGAACGAGATCCACGA
CATGGCGACGCCGCAGAACTCCAACATGCCCAACATGATGTACTACAACGAGAGGTCGCT
GGCGGACTTCTTCCCTCCTCACCTCCTCAAGAAGATGGTGTCGGAGGTGGTGTCGACGTT
CCTGCTGGTGTTCGTGACGTGCGGGGCGTCGGCGATCAACGGCAACGACCCGTCGCGCAT
ATCGCAGCTCGGGCAGTCGGTCGCCGGAGGGCTCATCGTCACCGTCATGATCTACTCCGT
CGGCCACATCTCCGGCGCCCACATGAACCCCGCCGTCACCACCGCCTTCGCCGTCTTCCG
CCATTTCCCATGGATTCAGGTTCCGTTCTACTGGGCGTCGCAGTTCACGGGGGCGATCTG
CGCGTCGTTCGTGCTGAAGGCGGTGCTGCACCCGATCGAGGTGCTGGGAACCACGACGCC
CGTGGGGCCGCACTGGCACTCGCTGCTCATCGAGATCATCGTCACCTTCAACATGATGTT
CGTCACCCTCGCCGTCGCCACGGACACCAGAGCGGTGGGTGAGTTGGCCGGGTTAGCTGT
CGGGTCCTCCGTTTGCATTACGTCCATCTTCGCAGGGGCGGTGTCGGGCGGATCGATGAA
CCCGGCTCGGACGCTGGGGCCGGCGTTGGCGAGCAACCGCTACACGGGGCTGTGGCTCTA
TTTCTTGGGCCCGATCCTAGGCACACTGTCGGGCGCCTGGACCTACACCTTCATCCGCTT
CGAGGACTCCCCCAAGGACGCCCCCCAGAAGCTCTCCTCCTTCAAGCTCCGACGCCTCCA
GAGCCAATCCGTCGCCGCCGAAGACGACGACGTCCTCGACCACATCCCCGTCTGATCAT

>Lsi1-Hv123404606

AACAATGGCCAGCAACTCGAGATCGAACTCCAGGGCGACCTTCTCCAGCGAGATCCACGA
CATCGGAACGGTGCAGAACTCCACCACGCCCAGCATGGTGTACTACACCGAGCGGTCCAT
CGCCGACTACTTCCCTCCTCACCTCCTCAAGAAGGTGGTGTCGGAGGTGGTGTCGACGTT
CCTGCTGGTGTTCGTGACGTGCGGGGCGGCGGCCATCAGCGCCCACGACGTCACGCGCAT
ATCGCAGCTCGGCCAGTCGGTCGCCGGCGGGCTCATCGTCGTCGTGATGATCTATGCCGT
CGGCCACATCTCCGGCGCCCACATGAACCCCGCCGTCACCCTCGCCTTCGCCATCTTCCG
CCATTTCCCCTGGATTCAGGTCCCGTTCTACTGGGCGGCGCAGTTCACGGGCGCCATCTG
CGCGTCCTTCGTGCTCAAGGCGGTGCTCCACCCCATCACCGTGATCGGCACCACCGAGCC
GGTGGGGCCGCACTGGCACGCTCTGGTCATCGAGGTCGTCGTCACCTTCAACATGATGTT
CGTCACCCTCGCCGTCGCCACGGACACTAGAGCGGTTGGTGAGTTGGCTGGGTTGGCTGT
CGGTTCCTCCGTTTGCATTACCTCCATCTTCGCAGGGGCGGTGTCAGGTGGATCGATGAA
CCCGGCGAGGACGCTGGGCCCGGCGCTGGCGAGCAACCGCTACCCTGGCCTCTGGCTCTA
CTTCCTGGGACCCGTCCTTGGCACGCTCAGCGGGGCCTGGACCTACACCTACATCCGCTT
CGAGGACCCGCCCAAGGACGCGCCCCAGAAGCTCTCCTCCTTCAAGCTCCGGCGGCTGCA
GAGCCAGTCCGTCGCCGCCGACGACGACGAGCTCGACCACATCCCCGTCTGATCCT

>Lsi1-TaCS6A02G307300

GGAAATGGCCACCAACTCGAGGTCGAACTCCAGGGCGACCTTCTCCAGCGAGATCCACGA
CATCGGCACGGTGCAGAACTCCGCCACGCCCAGCATGGTGTACTACACCGAGCGGTCCAT
CGCCGACTACTTCCCTCCCCACCTCCTCAAGAAGGTGGTGTCGGAGGTGGTGTCCACGTT
CCTGCTGGTGTTCGTGACGTGCGGGGCGGCGGCGATCAGCGCCCACGACGTCACGCGCAT
ATCGCAGCTCGGCCAGTCGGTCGCCGGCGGGCTCATCGTCGTCGTCATGATCTACGCCGT
CGGCCACATCTCCGGCGCGCACATGAACCCCGCCGTCACCCTCGCCTTCGCCATATTCCG
CCATTTCCCCTGGATTCAGGTCCCGTTCTACTGGGCGGCGCAGTTCACGGGCGCGATCTG
CGCGTCCTTCGTGCTCAAGGCCGTGCTCCACCCCATCACCGTGATCGGCACCACCGAGCC
GGTCGGGCCGCACTGGCACGCGCTGGTCATCGAGGTCGTCGTCACCTTCAACATGATGTT
CGTCACCCTCGCCGTCGCCACGGACACCAGAGCGGTGGGTGAGTTGGCTGGGTTGGCTGT
CGGTTCCTCCGTTTGCATTACCTCCATCTTCGCAGGGGCGGTGTCAGGTGGATCGATGAA
CCCGGCGAGGACGCTGGGCCCGGCGCTGGCCAGCAACCGCTACCCCGGCCTCTGGCTCTA
CTTCCTCGGCCCCGTCCTCGGCACGCTCTCCGGGGCCTGGACCTACACCTACATCCGCTT
CGAGGACGCGCCCAAGGACGGCCCCCAGAAGCTCTCCTCCTTCAAGCTCCGCCGGCTGCA
GAGCCAGTCCGTCGCCGCCGACGACGACGAGCTCGACCACATCCCCGTCTGATCCT

>Lsi1-TaCS6B02G335900

GAAAATGGCCACCAACTCGAGGTCGAACTCCAGGGCGACCTTCTCCAGCGAGATCCACGA
CATCGGCACGGTGCAGAACTCCACCACGCCCAGCATGGTGTACTACACCGAGCGGTCCAT
CGCCGACTACTTCCCTCCTCACCTCCTCAAGAAGGTGGTGTCGGAGGTGGTGTCGACGTT
CCTGCTGGTGTTCGTGACGTGCGGGGCGGCGGCGATCAGCGCCCACGACGTGACGCGCAT
ATCGCAGCTCGGCCAGTCGGTCGCCGGCGGGCTCATCGTCGTCGTGATGATCTATGCCGT
CGGCCACATCTCTGGCGCGCACATGAACCCCGCCGTCACCCTCGCCTTCGCCATCTTCCG
CCATTTCCCCTGGATTCAGGTCCCGTTCTACTGGGCGGCGCAGTTCACGGGCGCGATCTG
CGCGTCCTTCGTGCTCAAGGCGGTGCTCCACCCCATCACCGTGATCGGCACCACCGAGCC
GGTCGGGCCGCACTGGCACGCGCTGGTCATCGAGGTCGTCGTCACCTTCAACATGATGTT
CGTCACCCTCGCCGTCGCCACGGACACTAGAGCGGTGGGTGAGTTGGCTGGGTTGGCTGT
CGGTTCCTCCGTTTGCATTACCTCCATCTTCGCAGGGGCGGTGTCAGGTGGATCGATGAA

CCCGGCGAGGACGCTGGGCCCGGCGCTGGCCAGCAACCGCTACCCCGGCCTCTGGCTCTA
CTTCCTGGGCCCCGTCCTCGGCACGCTCTCCGGGGCCTGGACCTACACCTACATCCGCTT
CGAGGAGCCGCCCAAGGACGGGCCCCAGAAGCTCTCCTCCTTCAAGCTCCGGCGGCTGCA
GAGCCAGTCCGTGGCCGCCGACGACGACGAGCTCGACCACATCCCCGTCTGATCCT

>Lsi1-TaCS6D02G286400

GGAAATGGCCACCAACTCGAGGTCGAACTCCAGGGCCACCTTCTCCAGCGAGATCCACGA
CATCGGCACGGTGCAGAACTCCACCACGCCCAGCATGGTGTACTACACCGAGCGGTCCAT
CGCCGACTACTTCCCTCCTCACCTCCTCAAGAAGGTGGTGTCGGAGGTGGTGTCGACGTT
CCTGCTGGTGTTCGTGACGTGCGGGGCGGCGGCGATCAGCGCCCACGACGTCACGCGCAT
ATCGCAGCTCGGCCAGTCGGTCGCCGGCGGGCTCATCGTCGTCGTCATGATCTATGCCGT
CGGGCACATCTCCGGCGCGCACATGAACCCCGCCGTCACCCTCGCCTTCGCCATATTCCG
CCATTTCCCCTGGATTCAGGTCCCGTTCTACTGGGCGGCGCAGTTCACCGGCGCGATCTG
CGCGTCCTTCGTGCTCAAGGCGGTGCTCCACCCCATCACCGTGATCGGCACCACCGAGCC
GGTCGGGCCGCACTGGCACGCGCTGGTCATCGAGGTCGTCGTCACCTTCAACATGATGTT
CGTCACCCTCGCCGTCGCCACGGACACCAGAGCGGTGGGTGAGTTGGCTGGGTTGGCTGT
CGGTTCCTCCGTTTGCATTACCTCCATCTTCGCAGGGGCGGTGTCAGGTGGATCGATGAA
CCCGGCGAGGACGCTGGGCCCGGCGCTGGCCAGCAACCGCTACCCCGGCCTCTGGCTCTA
CTTCCTGGGCCCCGTCCTCGGCACGCTCAGCGGGGCCTGGACCTACACCTACATCCGCTT
CGAGGACCCGCCCAAGGACGGCCCCCAGAAGCTCTCCTCCTTCAAGCTCCGGCGGCTGCA
GAGCCAGTCCGTCGCCGCCGACGACGACGAGCTCGACCACATCCCCGTCTGATTCG

>PjLsi1

GGAAATGGCCACCAACTCGAGATCGAACTCCAGGGCGACCTTCTCCAGCGAGATCCACGA
CATCGCCACCGTGCAGAACTCCACCACGCCCAGCATGGTGTACTACACCGAGAGGTCCAT
CGCCGACTACTTCCCTCCTCATCTCCTCAAGAAGGTGGTGTCGGAGGTGGTGTCGACGTT
CCTGCTGGTGTTCGTGACGTGCGGGGCGGCGGCGATCAGCGCCCACGACGTCACGCGCAT
ATCGCAGCTCGGCCAGTCGGTCGCCGGCGGGCTCATCGTCGTCGTGATGATCTATGCCGT
CGGCCACATCTCCGGCGCGCACATGAACCCCGCCGTCACCCTCGCCTTCGCCATCTTCCG
GCATTTCCCCTGGATTCAGGTCCCGTTCTACTGGGCGGCGCAGTTCACGGGCGCGATCTG
CGCGTCGTTCGTTCTCAAGGCGGTGCTCCACCCCATCACCGTGATCGGCACCACCGAGCC
GGTGGGGCCGCACTGGCACGCGCTGGTCATCGAGATCGTCGTCACCTTCAACATGATGTT
CGTCACCCTCGCCGTCGCCACGGACACCAGAGCGGTGGGTGAGTTGGCTGGGTTGGCTGT
CGGTTCCTCCGTTTGCATTACCTCCATCTTCGCAGGGGCGGTGTCAGGTGGATCGATGAA
CCCGGCGAGGACGCTGGGGCCGGCGCTGGCGAGCAACCGCTACTCCGGCCTCTGGCTCTA
CTTCCTGGGCCCCGTCCTCGGCACGCTCAGCGGGGCCTGGACCTACACCTACATCCGCTT
CGAGGACCCGCCCAAGGACGCGCCCCAGAAGCTCTCCTCCTTCAAGCTCCGCCGGCTGCA
GAGCCAGTCCGTCGCCGCCGACGACGACCTCGACCACATCCCGGTCTGATCCA

Clustal-W for *Lsi1* genes, CDS. Designed primers are indicated by different colours as shown in Supplementary material S2.

```
TraesCS6A02G307300      GGAAATGGCCACCAACTCGAGGTCGAACTCCAGGGCGACCTTCTCCAGCGAGATCCACGA
TraesCS6D02G286400      GGAAATGGCCACCAACTCGAGGTCGAACTCCAGGGCCACCTTCTCCAGCGAGATCCACGA
TraesCS6B02G335900      GAAAATGGCCACCAACTCGAGGTCGAACTCCAGGGCGACCTTCTCCAGCGAGATCCACGA
LOC-Hv123404606         AACAATGGCCAGCAACTCGAGATCGAACTCCAGGGCGACCTTCTCCAGCGAGATCCACGA
P-juncea-Lsi1           GGAAATGGCCACCAACTCGAGATCGAACTCCAGGGCGACCTTCTCCAGCGAGATCCACGA
                           ******** ********* ************** ***********************

TraesCS6A02G307300      CATCGGCACGGTGCAGAACTCCGCCACGCCCAGCATGGTGTACTACACCGAGCGGTCCAT
TraesCS6D02G286400      CATCGGCACGGTGCAGAACTCCACCACGCCCAGCATGGTGTACTACACCGAGCGGTCCAT
TraesCS6B02G335900      CATCGGCACGGTGCAGAACTCCACCACGCCCAGCATGGTGTACTACACCGAGCGGTCCAT
LOC-Hv123404606         CATCGGAACGGTGCAGAACTCCACCACGCCCAGCATGGTGTACTACACCGAGCGGTCCAT
P-juncea-Lsi1           CATCGCCACCGTGCAGAACTCCACCACGCCCAGCATGGTGTACTACACCGAGAGGTCCAT
                        *****  ** ************ ***************************** *******

TraesCS6A02G307300      CGCCGACTACTTCCCTCCCCACCTCCTCAAGAAGGTGGTGTCGGAGGTGGTGTCCACGTT
TraesCS6D02G286400      CGCCGACTACTTCCCTCCTCACCTCCTCAAGAAGGTGGTGTCGGAGGTGGTGTCGACGTT
TraesCS6B02G335900      CGCCGACTACTTCCCTCCTCACCTCCTCAAGAAGGTGGTGTCGGAGGTGGTGTCGACGTT
LOC-Hv123404606         CGCCGACTACTTCCCTCCTCACCTCCTCAAGAAGGTGGTGTCGGAGGTGGTGTCGACGTT
P-juncea-Lsi1           CGCCGACTACTTCCCTCCTCATCTCCTCAAGAAGGTGGTGTCGGAGGTGGTGTCGACGTT
                        ****************** ** ******************************** *****

TraesCS6A02G307300      CCTGCTGGTGTTCGTGACGTGCGGGGCGGCGGCGATCAGCGCCCACGACGTCACGCGCAT
TraesCS6D02G286400      CCTGCTGGTGTTCGTGACGTGCGGGGCGGCGGCGATCAGCGCCCACGACGTCACGCGCAT
TraesCS6B02G335900      CCTGCTGGTGTTCGTGACGTGCGGGGCGGCGGCGATCAGCGCCCACGACGTGACGCGCAT
LOC-Hv123404606         CCTGCTGGTGTTCGTGACGTGCGGGGCGGCGGCCATCAGCGCCCACGACGTCACGCGCAT
P-juncea-Lsi1           CCTGCTGGTGTTCGTGACGTGCGGGGCGGCGGCGATCAGCGCCCACGACGTCACGCGCAT
                        ********************************* ***************** ********

TraesCS6A02G307300      ATCGCAGCTCGGCCAGTCGGTCGCCGGCGGGCTCATCGTCGTCGTCATGATCTACGCCGT
TraesCS6D02G286400      ATCGCAGCTCGGCCAGTCGGTCGCCGGCGGGCTCATCGTCGTCGTCATGATCTATGCCGT
TraesCS6B02G335900      ATCGCAGCTCGGCCAGTCGGTCGCCGGCGGGCTCATCGTCGTCGTGATGATCTATGCCGT
LOC-Hv123404606         ATCGCAGCTCGGCCAGTCGGTCGCCGGCGGGCTCATCGTCGTCGTGATGATCTATGCCGT
P-juncea-Lsi1           ATCGCAGCTCGGCCAGTCGGTCGCCGGCGGGCTCATCGTCGTCGTGATGATCTATGCCGT
                        ********************************************* ******** *****

TraesCS6A02G307300      CGGCCACATCTCCGGCGCGCACATGAACCCCGCCGTCACCCTCGCCTTCGCCATATTCCG
TraesCS6D02G286400      CGGGCACATCTCCGGCGCGCACATGAACCCCGCCGTCACCCTCGCCTTCGCCATATTCCG
TraesCS6B02G335900      CGGCCACATCTCTGGCGCGCACATGAACCCCGCCGTCACCCTCGCCTTCGCCATCTTCCG
LOC-Hv123404606         CGGCCACATCTCCGGCGCCCACATGAACCCCGCCGTCACCCTCGCCTTCGCCATCTTCCG
P-juncea-Lsi1           CGGCCACATCTCCGGCGCGCACATGAACCCCGCCGTCACCCTCGCCTTCGCCATCTTCCG
                        *** ******** ***** *********************************** *****

TraesCS6A02G307300      CCATTTCCCCTGGATTCAGGTCCCGTTCTACTGGGCGGCGCAGTTCACGGGCGCGATCTG
TraesCS6D02G286400      CCATTTCCCCTGGATTCAGGTCCCGTTCTACTGGGCGGCGCAGTTCACCGGCGCGATCTG
TraesCS6B02G335900      CCATTTCCCCTGGATTCAGGTCCCGTTCTACTGGGCGGCGCAGTTCACGGGCGCGATCTG
LOC-Hv123404606         CCATTTCCCCTGGATTCAGGTCCCGTTCTACTGGGCGGCGCAGTTCACGGGCGCCATCTG
P-juncea-Lsi1           GCATTTCCCCTGGATTCAGGTCCCGTTCTACTGGGCGGCGCAGTTCACGGGCGCGATCTG
                         *********************************************** ***** *****

TraesCS6A02G307300      CGCGTCCTTCGTGCTCAAGGCCGTGCTCCACCCCATCACCGTGATCGGCACCACCGAGCC
TraesCS6D02G286400      CGCGTCCTTCGTGCTCAAGGCGGTGCTCCACCCCATCACCGTGATCGGCACCACCGAGCC
TraesCS6B02G335900      CGCGTCCTTCGTGCTCAAGGCGGTGCTCCACCCCATCACCGTGATCGGCACCACCGAGCC
LOC-Hv123404606         CGCGTCCTTCGTGCTCAAGGCGGTGCTCCACCCCATCACCGTGATCGGCACCACCGAGCC
P-juncea-Lsi1           CGCGTCGTTCGTTCTCAAGGCGGTGCTCCACCCCATCACCGTGATCGGCACCACCGAGCC
                        ****** ***** ******** **************************************
```

```
TraesCS6A02G307300      GGTCGGGCCGCACTGGCACGCGCTGGTCATCGAGGTCGTCGTCACCTTCAACATGATGTT
TraesCS6D02G286400      GGTCGGGCCGCACTGGCACGCGCTGGTCATCGAGGTCGTCGTCACCTTCAACATGATGTT
TraesCS6B02G335900      GGTCGGGCCGCACTGGCACGCGCTGGTCATCGAGGTCGTCGTCACCTTCAACATGATGTT
LOC-Hv123404606         GGTGGGGCCGCACTGGCACGCTCTGGTCATCGAGGTCGTCGTCACCTTCAACATGATGTT
P-juncea-Lsi1           GGTGGGGCCGCACTGGCACGCGCTGGTCATCGAGATCGTCGTCACCTTCAACATGATGTT
                        *** ***************** ************ *************************

TraesCS6A02G307300      CGTCACCCTCGCCGTCGCCACGGACACCAGAGCGGTGGGTGAGTTGGCTGGGTTGGCTGT
TraesCS6D02G286400      CGTCACCCTCGCCGTCGCCACGGACACCAGAGCGGTGGGTGAGTTGGCTGGGTTGGCTGT
TraesCS6B02G335900      CGTCACCCTCGCCGTCGCCACGGACACTAGAGCGGTGGGTGAGTTGGCTGGGTTGGCTGT
LOC-Hv123404606         CGTCACCCTCGCCGTCGCCACGGACACTAGAGCGGTTGGTGAGTTGGCTGGGTTGGCTGT
P-juncea-Lsi1           CGTCACCCTCGCCGTCGCCACGGACACCAGAGCGGTGGGTGAGTTGGCTGGGTTGGCTGT
                        *************************** ******** ***********************

TraesCS6A02G307300      CGGTTCCTCCGTTTGCATTACCTCCATCTTCGCAGGGGCGGTGTCAGGTGGATCGATGAA
TraesCS6D02G286400      CGGTTCCTCCGTTTGCATTACCTCCATCTTCGCAGGGGCGGTGTCAGGTGGATCGATGAA
TraesCS6B02G335900      CGGTTCCTCCGTTTGCATTACCTCCATCTTCGCAGGGGCGGTGTCAGGTGGATCGATGAA
LOC-Hv123404606         CGGTTCCTCCGTTTGCATTACCTCCATCTTCGCAGGGGCGGTGTCAGGTGGATCGATGAA
P-juncea-Lsi1           CGGTTCCTCCGTTTGCATTACCTCCATCTTCGCAGGGGCGGTGTCAGGTGGATCGATGAA
                        ************************************************************

TraesCS6A02G307300      CCCGGCGAGGACGCTGGGCCCGGCGCTGGCCAGCAACCGCTACCCCGGCCTCTGGCTCTA
TraesCS6D02G286400      CCCGGCGAGGACGCTGGGCCCGGCGCTGGCCAGCAACCGCTACCCCGGCCTCTGGCTCTA
TraesCS6B02G335900      CCCGGCGAGGACGCTGGGCCCGGCGCTGGCCAGCAACCGCTACCCCGGCCTCTGGCTCTA
LOC-Hv123404606         CCCGGCGAGGACGCTGGGCCCGGCGCTGGCGAGCAACCGCTACCCTGGCCTCTGGCTCTA
P-juncea-Lsi1           CCCGGCGAGGACGCTGGGGCCGGCGCTGGCGAGCAACCGCTACTCCGGCCTCTGGCTCTA
                        ****************** *********** ************ * **************

TraesCS6A02G307300      CTTCCTCGGCCCCGTCCTCGGCACGCTCTCCGGGGCCTGGACCTACACCTACATCCGCTT
TraesCS6D02G286400      CTTCCTGGGCCCCGTCCTCGGCACGCTCAGCGGGGCCTGGACCTACACCTACATCCGCTT
TraesCS6B02G335900      CTTCCTGGGCCCCGTCCTCGGCACGCTCTCCGGGGCCTGGACCTACACCTACATCCGCTT
LOC-Hv123404606         CTTCCTGGGACCCGTCCTTGGCACGCTCAGCGGGGCCTGGACCTACACCTACATCCGCTT
P-juncea-Lsi1           CTTCCTGGGCCCCGTCCTCGGCACGCTCAGCGGGGCCTGGACCTACACCTACATCCGCTT
                        ****** ** ******** *********  ******************************

TraesCS6A02G307300      CGAGGACGCGCCCAAGGACGGCCCCCAGAAGCTCTCCTCCTTCAAGCTCCGCCGGCTGCA
TraesCS6D02G286400      CGAGGACCCGCCCAAGGACGGCCCCCAGAAGCTCTCCTCCTTCAAGCTCCGGCGGCTGCA
TraesCS6B02G335900      CGAGGAGCCGCCCAAGGACGGGCCCCAGAAGCTCTCCTCCTTCAAGCTCCGGCGGCTGCA
LOC-Hv123404606         CGAGGACCCGCCCAAGGACGCGCCCCAGAAGCTCTCCTCCTTCAAGCTCCGGCGGCTGCA
P-juncea-Lsi1           CGAGGACCCGCCCAAGGACGCGCCCCAGAAGCTCTCCTCCTTCAAGCTCCGCCGGCTGCA
                        ******  ************  ***************************** ********

TraesCS6A02G307300      GAGCCAGTCCGTCGCCGCCGACGACGACGAGCTCGACCACATCCCCGTCTGATCCT
TraesCS6D02G286400      GAGCCAGTCCGTCGCCGCCGACGACGACGAGCTCGACCACATCCCCGTCTGATTCG
TraesCS6B02G335900      GAGCCAGTCCGTGGCCGCCGACGACGACGAGCTCGACCACATCCCCGTCTGATCCT
LOC-Hv123404606         GAGCCAGTCCGTCGCCGCCGACGACGACGAGCTCGACCACATCCCCGTCTGATCCT
P-juncea-Lsi1           GAGCCAGTCCGTCGCCGCCGACGACGAC---CTCGACCACATCCCGGTCTGATCCA
                        ************ ***************   ************** ******* *
```

Lsi2

In CDS sequences, 'Start'- and 'Stop'-codons are highlighted by green and red, respectively. Exons are indicated by different colours on example of *P. juncea* sequence.

>Lsi2-Os03g01700

GAAGATGAGTGAGCTTGCGTCGGCGCCCAAGGTGGCGCTTGGATCGATCGCGTTCGCGGT
GTTCTGGATGATGGCGGTGTTCCCGTCGGTGCCGTTCCTGCCGATCGGGCGGACGGCGGG
GTCGCTGCTGAGCGCGGTGCTGATGGTGATATTCCACGTGATCAGCCCCGACGACGCGTA
CGCCTCCATCGACCTCCCAATCCTGGGCCTCCTCTTCGCCACCATGGTGGTGGGCAGCTA
CCTCCGGAACGCCGGGATGTTCAAGCACCTGGGGCGTCTGCTGGCGTGGAAGAGCCAGGG
CGGGCGCGACCTCATGTGCCGCGTCTGCGTCGTCACCGCCCTCGCCAGCGCCCTCTTCAC
CAACGACACCTGCTGCGTCGTCCTCACCGAGTTCGTCCTCGAGCTCGCCGCCGAGCGCAA
CCTCCCCGCCAAGCCCTTCCTCCTCGCCCTCGCCTCCAGCGCCAACATCGGCTCCGCCGC
CACCCCCATCGGCAACCCCCAGAACCTGGTCATCGCCTTCAATAGCAAGATCACCTTCCC
CAAGTTCCTCATGGGAATCCTCCCGGCCATGCTCGTCGGGATGGCCGTCAACATGGTCAT
GCTGCTCTGCATGTACTGGAGGGAGCTGGGCGGAGGGGCCGAGCTCAGCGTCGACGGCAA
GCAGATGGAGGCGGTGGAGGAAGGCAGGTCGCCGGCGTCGGCCAAGAGCACGCCGCAGCT
GAACGGCAACGGCAACACGATGATGTCGCTGGAGATGTCGGAGAACATAACGACCAAGCA
CCCATGGTTCATGCAGTGCACGGAGGCGCGGCGGAAGCTGTTCCTCAAGAGCTTCGCGTA
CGTGGTGACGGTGGGGATGGTGGTGGCCTACATGGTGGGGCTCAACATGTCGTGGACGGC
CATCACCACGGCGCTGGCGCTGGTGGTGGTCGACTTCCGCGACGCCGAGCCGTGCCTGGA
CACCGTGTCCTACTCGCTGCTCGTCTTCTTCTCCGGGATGTTCATCACCGTCAGCGGCTT
CAACAAGACGGGCCTCCCGGGAGCCATCTGGGACTTCATGGCCCCCTACTCCAAGGTCAA
CAGCGTCGGCGGCATCTCCGTCCTCTCCGTCATCATCCTCCTCCTCTCCAACCTCGCATC
AAACGTACCAACGGGTAATTATACATTCTATTAATCCAGAATTAATGTATACTACTCCAT
ATAAGTATAACTAATTAACATACAAAAATATATATGCGCATGCATGCAGTGCTTCTTATG
GGTGATGAGGTGGCGAAGGCGGCGGCGCTGATATCGCCGGCGGCGGTGACGACGTCGTGG
CTGCTGCTGGCGTGGGTGAGCACGGTGGCGGGGAACCTGTCGCTGCTGGGGTCGGCGGCG
AACCTGATAGTGTGCGAGCAGGCGAGGAGGGCGCCCAGGAACGCCTACGACCTCACCTTC
TGGCAGCACATCGTCTTCGGCGTCCCATCCACCCTCATCGTCACCGCCGTCGGCATACCC
CTCATCGGCAAGATCTGATCTCA

>Lsi2-Bradi1g78100

CATCATGGTGCTCGCATCTCTACCCAAGGTGGTCCTTGGCTCGATCGCCTTCGGCATCTT
CTGGATGATGGCGGTGTTCCCGTCGGTGCCATTCCTCCCCATCGGACGGACCGCCGGCTC
GCTCCTCTCGGCGGTCCTCATGATCGTCTTCCACGTCATCAGCCCGGACGATGCCTATGC
CTCCATCGACCTCCCGATCCTCGGCCTCCTCTTCTCCACCATGGTCGTCGGCGGCTACCT
CAAGAACGCTGGCATGTTCAAGCATCTCGGAACCCTCCTAGCATGGAAGTCCCAGGGAGG
GCGTGACCTGCTCTGCCGGGTCTGCGTCGTCACAGCCCTTGCCTCGGCCCTCTTCACCAA
CGACACCTGCTGCGTCGTGCTCACTGAGTTCGTCCTCGAGCTCGCCGCCGAGAGGAACCT
CCCGGCCAAGCCCTTCTTGCTGGCCTTGGCGTCCAGTGCCAATATTGGGTCCAGTGCCAC
CCCTATTGGGAACCCACAGAATCTGGTCATAGCATTCAACAGCAAAATCCCCTTCCCCAA
GTTCTTGCTTGGGATTCTGCCGGCCATGCTCGCTGGCATGGCTGTCAACATGGTGATGCT
GCTAGCCATGTACTGGAAGGACCTTGAGGGTACGAGCCCTTGCATGGCCAACAACGGTAA
GCAGATGGAGGCCATCGAGGAAGGCCGGTCTCCCTCCGTGGCATCCCTCAAGAGCCCGCA
CCCGATCACGGGAGCCATGGTCGTCGATGACGGCGACTCAATGATGGAGGAGAACATATC

CACCAAGCACCCGTGGTTCATGCAGTGCACAGAGCAGCGGAGGAAGCTGTTCCTCAAGAG
CTTCGCCTACATCGTGACCGTCGGCATGGTGGTGGCCTACATGGTCGGGCTGAACATGTC
GTGGACGGCCATCACGACGGCAATCGCCCTAGTCGTCGTCGATTTCAGGGATGCCGAGCC
GTGCTTGGTGAAGGTGTCCTATTCGCTGCTGGTCTTCTTCTCCGGGATGTTTATCACGGT
GAGTGGGTTCAATAAGACAGGGCTCCCCGGAGCTATTTGGGACTTCATGGCGCCCTACTC
CAAGGTTGACAGCGTTGGTGGGATATCTGTGCTGTCTGTCATCATCTTGCTCCTCTCCAA
CCTCGCCTCCAACGTACCAACAGTGCTGCTGATGGGTAACGAGGTTGCCACGGCAGCGGC
GCTGATATCGCCGGCAGCGGTGACGCGGTCGTGGCTGCTTCTGGCATGGGTGAGCACGGT
GGCTGGGAACCTGTCGCTGCTGGGGTCGGCGGCGAACCTGATCGTGTGCGAGCAGGCGCG
CCGGGCCCCACGGAACGCCTACGAGCTCACCTTCTGGAACCATCTTGTCTTCGGCGTCCC
CTCCACCCTCATCGTCACCGCTGTCGGCATCCCCCTCATCGGAAAGATGTAGCCACC

>Lsi2-Hv123449943

AGCCATGGCGCTCGCGTCTCTCCCCAAGGTGGTGCTGGGCTCCTTCGCCTTCGCCGTCTT
CTGGATGATGGCGGTGTTCCCGTCGGTGCCGTTTCTCCCCATCGGCCGGACGGCGGGGTC
GCTGCTCTCCGCCGTGCTCATGATCGTCTTCCACGTGATCAGCCCCGACGACGCCTACGC
CTCCATCGACCTCCCCATCCTCGGCCTGCTCTTCTCCACCATGGTCGTCGGTGGGTACCT
CAAGAACGCCGGCATGTTCAAGCACCTCGGCACCCTCCTCGCGTGGAAGAGCCAGGGCGG
CCGCGACCTGCTCTGCCGCGTCTGCGTCGTCACCGCGCTCGCCTCCGCGCTCTTCACCAA
CGACACCTGCTGCGTCGTGCTCACCGAGTTCGTGCTCGAGCTCGCCGCCGAGCGCAACCT
GCCCGCCAAGCCCTTCCTCCTCGCGCTCGCCTCCAGCGCCAACATCGGCTCCAGCGCCAC
CCCCATCGGCAACCCGCAGAACCTCGTCATCGCCTTCAACAGCAAGATCTCCTTCCCCAG
GTTCCTCATCGGGATCCTGCCGGCCATGCTCGCCGGCATGGCCGTCAACATGGTCATGCT
GCTCTGCATGTACTGGAAGGACCTCGAGGGCGTGGCCCCCGACGCCGACGGCAAGCAGAT
GGCCGTCGTGGAGGAGGGCGGCCGCTCGCCGTCCGTGGCGTCGCTCAAGAGCCCGCACCC
CTTCAACGGCACCATGGCCGACGACGGGAACGAGTCGATGATGGAGGAGAACCTCTCCAC
CAAGCACCCCTGGTTCATGCAGTGCACGGAGCACCGGCGCAAGCTCTTCCTCAAGAGCTT
CGCCTACATCGTGACGGTGGGCATGGTGGTGGCCTACATGGCCGGGCTCAACATGTCGTG
GACGGCCATCACCACCGCCATCGCGCTGGTTGTCGTAGACTTCCGGGACGCCGAGCCCTG
CCTCGTCAAGGTCTCCTACTCGCTGCTCGTCTTCTTCTCCGGCATGTTCATCACCGTCAG
CGGCTTCAACAAGACGGGGCTGCCGGGCGCCATCTGGAACTTCATGGCGCCCTACTCCAA
GGTGGACAGCGCCGGCGGCATCTCCGTGCTCTCCGTCATCATCCTCCTCCTCTCCAACCT
CGCCTCCAACGTACCAACAGTGCTGCTGATGGGGAACGAGGTGGCTACCGCGGCGGCGCT
CATCTCGCCGGCAGCGGTGACGCGGTCCTGGCTGTTGCTGGCGTGGGTGAGCACGGTGGC
GGGCAACCTGTCGCTGCTAGGGTCAGCAGCGAACCTGATCGTGTGCGAGCAGGCACGCCG
GGCGCCCCGCAACGCCTACGAGCTCACCTTCTGGAACCACCTCATCTTCGGCGTGCCCTC
CACCCTCATCGTCACCGCCGTCGGCATCCCCCTCATCGGAAAGATGTAGCCACC

>Lsi2-TaCS5A02G529900

AGCCATGGCGCTCGCGTCTCTCCCCAAGGTGGTGCTCGGCTCCATCGCCTTCGCCGTCTT
CTGGATGATGGCGGTGTTCCCGTCGGTGCCCTTCCTGCCCATCGGCCGCACGGCGGGCTC
GCTGCTCTCCGCCGTGCTCATGATCGTCTTCCACGTGATCAGCCCCGACGACGCCTACGC
CTCCATCGACCTCCCCATCCTCGGCCTGCTCTTCTCCACCATGGTCGTCGGTGGCTACCT
CAAGAACGCCGGCATGTTCAAGCACCTCGGCACGCTCCTCGCCTGGAAGAGCCAGGGCGG
CCGCGACCTGCTCTGCCGCGTCTGCGTCGTCACCGCGCTGGCCTCGGCGCTCTTCACCAA
CGACACCTGCTGCGTCGTGCTCACCGAGTTCGTGCTCGAGCTCGCCGCCGAGCGGAACCT
CCCGGCCAAGCCCTTCCTCCTGGCCCTCGCCTCCAGCGCCAACATCGGCTCCAGCGCCAC

CCCCATCGGCAACCCGCAGAACCTGGTCATCGCCTTCAACAGCAAGATCTCCTTCCCCAG
GTTCCTCATCGGCATCCTGCCGGCCATGCTCGCCGGCATGGCCGTCAACATGGTCATGCT
GCTCTGCATGTACTGGAAGGACCTCGAGGGTGTGGCCCCCGACGCGGCCGGCAAGCAGAT
GTCGGTCGTCGAGGAGGGCGGCCGCTCGCCGTCCGTGGCGTCGCTCAAGAGCCCGCACCC
GTTCAACGGCACCACGGCCGACGACGGCAACGAGTCGATGATGGAGGAGAACATCTCGAC
CAAGCACCCGTGGTTCATGCAGTGCACGGAGCACCGACGCAAGCTGTTCCTCAAGAGCTT
CGCCTACATCGTGACGCTGGGCATGGTGGTGGCCTACATGGCTGGGCTCAACATGTCGTG
GACGGCCATCACCACCGCCATCGCGCTGGTCGTCGTCGACTTCCGGGACGCCGAGCCGTG
CCTCGTCAAGGTCTCCTACTCGCTGCTCGTCTTCTTCTCCGGCATGTTCATCACGGTGAG
CGGGTTCAACAAGACGGGGCTGCCGGGCGCCATCTGGAACTTCATGGCGCCCTACTCCAA
GGTGGACAGCGCCGGCGGCATCTCCGTGCTCTCCGTCATCATCCTCCTCCTCTCCAACCT
CGCCTCCAACGTACCAACAGTGCTGCTGATGGGGAACGAGGTGGCGACCGCAGCGGCCCT
GATCTCGCCGGCAGCGGTGACGCGGTCGTGGCTGCTGCTGGCGTGGGTGAGCACGGTGGC
AGGGAATCTCTCGCTGCTGGGGTCGGCGGCGAACCTGATCGTGTGTGAGCAGGCGCGTCG
GGCGCCGCGTAACGCCTATGAGCTCACCTTCTGGAACCACCTCATCTTTGGCGTGCCCTC
CACCCTCATCGTCACCGCCGTCGGCATCCCCCTCATCGGAAAGATGTAGCCACC

>Lsi2-TaCS4B02G361900
AGCCATGGCGCTCGCGTCTCTCCCCAAGGTGGTGCTCGGCTCCGTCGCCTTCGCCATCTT
CTGGATGATGGCCGTGTTCCCGTCGGTGCCCTTCCTGCCCATCGGCCGGACGGCGGGGTC
GCTGCTCTCCGCCGTGCTCATGATCGTCTTCCACGTGATCAGCCCCGACGACGCCTACGC
CTCCATCGACCTCCCCATCCTCGGCCTGCTCTTCTCCACCATGGTCGTCGGCGGCTACCT
CAAGAACGCCGGCATGTTCAAGCACCTCGGCACCCTGCTCGCCTGGAAGAGCCAGGGCGG
CCGCGACCTGCTCTGCCGGGTCTGCGTGGTGACCGCGCTCGCCTCCGCGCTCTTCACCAA
CGACACCTGCTGCGTCGTGCTCACCGAGTTCGTGCTCGAGCTCGCCGCCGAGCGGAACCT
TCCGGCCAAGCCCTTCCTCCTGGCCCTCGCCTCCAGCGCCAACATCGGTTCCAGCGCCAC
CCCCATCGGCAACCCGCAGAACCTGGTCATCGCCTTCAACAGCAAGATCTCGTTTCCAAA
GTTTTTGATCGGCATCCTGCCGGCCATGCTCGCCGGCATGGCCGTCAACATGGTCATGCT
GCTCTGCATGTACTGGAAGGACCTGGAGGGCGTGGCCCCCGACGCGGCCGGCAAGCAGAT
GTCGGTCGTCGAGGAGGGGGGCCGCTCGCCGTCCGTGGCATCGCTCAAGAGCCCGCACCC
GTTCAACGGCACCACGGCCGACGACGGGAACGAGTCGATGATGGAGGAGAACATCTCGAC
CAAGCACCCGTGGTTCATGCAGTGCACGGAGCACCGGCGCAAGCTGTTCCTCAAGAGCTT
CGCCTACATCGTGACGCTGGGCATGGTGGTAGCATACATGGCCGGGCTCAACATGTCGTG
GACGGCCATCACCACGGCCATCGCGCTGGTCGTCGTCGACTTCCGGGACGCCGAGCCGTG
CCTCGTCAAGGTCTCCTACTCGCTGCTCGTCTTCTTCTCCGGCATGTTCATCACGGTGAG
CGGGTTCAACAAGACGGGGCTGCCGGGCGCCATCTGGAACTTCATGGCGCCCTACTCCAA
GGTGGACAGCGCCGGCGGCATCTCCGTGCTCTCCGTCATCATCCTCCTCCTCTCCAACCT
CGCCTCCAACGTACCAACAGTGCTGCTGATGGGGAACGAGGTGGCGACCGCGGCGGCTCT
GATCTCCCCGGCGGCGGTGACTCGGTCGTGGCTGCTGCTGGCGTGGGTGAGCACGGTGGC
GGGCAACCTTTCGCTGCTGGGGTCGGCGGCGAACCTGATCGTGTGCGAGCAGGCGCGCCG
GGCGCCGCGCAACGCCTACGAGCTCACCTTCTGGAACCACCTCATCTTCGGCGTGCCCTC
CACCCTCATCGTCACCGCCGTCGGCATCCCCCTCATCGGAAAGATGTAGCCACC

>Lsi2-TaCS4D02G354900
AGCCATGGCGCTCGCGTCCCTCCCCAAGGTGGTGCTCGGCTCCTTCGCCTTCGCCATCTT
CTGGATGATGGCCGTGTTCCCGTCGGTGCCCTTCCTGCCCATCGGCCGGACGGCGGGCTC
GCTGCTCTCCGCCGTGCTCATGATCGTCTTCCACGTGATCAGCCCCGACGACGCCTACGC

CTCCATCGACCTCCCCATCCTCGGCCTGCTCTTCTCCACCATGGTCGTCGGCGGCTACCT
CAAGAACGCCGGCATGTTCAAGCACCTCGGCACGCTCCTCGCCTGGAAGAGCCAGGGCGG
CCGCGACCTGCTCTGCCGCGTCTGCGTCGTCACGGCGCTCGCCTCGGCGCTCTTCACCAA
CGACACCTGCTGCGTCGTGCTCACCGAGTTCGTGCTCGAGCTCGCCGCCGAGCGGAACCT
CCCGGCCAAGCCCTTCCTCCTCGCCCTCGCCTCCAGCGCCAACATCGGCTCCAGCGCCAC
CCCCATCGGCAACCCGCAGAACCTGGTCATCGCCTTCAACAGCAAGATCTCCTTCCCCAG
GTTCCTCATCGGCATCCTGCCGGCCATGCTCGCCGGCATGGCCGTCAACATGGTCATGCT
GCTCTGCATGTACTGGAAGGACCTGGAGGGCGTGGCCCCCGACGCGGCCGGCAAGCAGAT
GTCGGTCGTCGAGGAGGGCGGCCGCTCGCCGTCCGTGGCGTCGCTCAAGAGCCCGCACCC
GTTCAACGGCACCACCGCCGACGACGGGAACGAGTCGATGATGGAGGAGAACATCTCGAC
CAAGCACCCGTGGTTCATGCAGTGCACGGAGCACCGGCGCAAGCTGTTCCTCAAGAGCTT
TGCCTACATCGTGACGCTGGGCATGGTGGTGGCATACATGGCCGGGCTCAACATGTCGTG
GACGGCCATCACCACCGCCATCGCGCTCGTCGTCGTCGACTTCCGGGACGCCGAGCCTTG
CCTCGTCAAGGTCTCCTACTCGCTGCTCGTCTTCTTCTCCGGCATGTTCATCACGGTGAG
TGGGTTCAACAAGACGGGGCTGCCGGGCGCCATCTGGAACTTCATGGCGCCCTACTCCAA
GGTGGACAGCGCCGGCGGCATCTCCGTGCTCTCCGTCATCATCCTCCTCCTCTCCAACCT
CGCCTCCAACGTACCAACAGTGCTACTGATGGGGAACGAGGTGGCGACCGCGGCGGCTCT
GATCTCCCTGGCGGCGGTGACTCGGTCGTGGCTGCTGTTGGCGTGGGTGAGCACGGTGGC
GGGCAACCTGTCGCTGCTGGGGTCGGCGGCGAACCTGATCGTGTGCGAGCAGGCGCGCCG
GGCGCCGCGCAACGCCTACGAGCTCACCTTCTGGAACCACCTCATCTTCGGCGTGCCCTC
CACCCTCATCGTCACCGCCGTCGGCATCCCCCTCATCGGAAAGATGTAGCCACC

>PjLsi2

AGCCATGGCGCTCGCGTCTCTCCCCAAGGTGGTGCTGGGCTCCTTCGCCTTCGCCATCTT
CTGGATGATGGCGGTGTTCCCGTCGGTGCCCTTCCTGCCCATCGGCCGGACGGCGGGGTC
GCTGCTCTCCGCCGTGCTCATGATCGTCTTCCACGTGATCAGCCCCGACGACGCCTACGC
CTCCATCGACCTCCCCATCCTCGGCCTGCTCTTCTCCACCATGGTCGTCGGTGGCTACCT
CAAGAACGCCGGCATGTTCAAGCACCTGGGCACGCTCCTGGCCTGGAAGAGCCAGGGCGG
GCGCGACCTGCTCTGCCGCGTATGCGTCGTCACCGCGCTGGCCTCGGCGCTCTTCACCAA
CGACACCTGCTGCGTCGTGCTCACCGAGTTCGTGCTCGAGCTCGCCGCTGAGCGGAACCT
CCCGGCCAAGCCCTTCCTCCTCGCGCTCGCCTCCAGCGCCAACATCGGCTCCAGTGCCAC
CCCCATCGGCAACCCGCAGAATCTGGTCATCGCCTTCAACAGCAAGATCTCCTTCCCCAA
GTTTTTGATCGGCATCCTGCCAGCCATGCTCGCCGGCATGGCCGTCAACATGGTCATGCT
GCTCTGCATGTACTGGAAGGACCTTGAGGGCGTCGCCCCCGACGCGGCCGGCAAGCAGAT
GACGGTCGTGGAGGAGGGCCGCTCGCCATCCGTGGCATCGCACAAGAGCCCGCACCCGTT
CAACGGCACCGTGGCCGACGACGGGGCCGAGTCAATGATGGAGGAGAACATCTCGACCAA
GCACCCATGGTTCATGCAGTGCACGGAGCACCGGCGCAAGCTGTTCCTCAAGAGCTTCGC
CTACATCGTGACGGTGGGCATGGTGGTGGCCTACATGGCCGGGCTCAACATGTCGTGGAC
GGCCATCACCACCGCCATCGCGCTCGTCGTCGTCGACTTCCGGGACGCCGAGCCCTGCCT
CGTCAAGGTCTCCTACTCGCTGCTCGTCTTCTTCTCCGGCATGTTCATCACGGTGAGTGG
GTTCAACAAGACGGGGCTGCCGGGCGCCATCTGGAACTTCATGGCGCCCTACTCCAAGGT
CGACAGCGCCGGCGGCATCTCCGTGCTCTCCGTCATCATCCTCCTCCTCTCCAACCTCGC
ATCCAACGTACCCACAGTGCTGCTGATGGGGAACGAGGTGGCCACCGCGGCGGCGCTGAT
CTCGCCGGCAGCGGTGACGCGGTCGTGGCTGCTGCTAGCGTGGGTGAGCACGGTGGCGGG
CAACCTGTCGCTGCTGGGGTCGGCGGCGAACCTGATCGTGTGTGAGCAGGCGCGCCGGGC
GCCGCGGAACGCCTACGAGCTCACCTTCTGGAACCACCTCGTCTTTGGCGTGCCCTCCAC
CCTCATCGTCACCGCCGTCGGCATCCCCCTCATCGGAAAGATGTAGCCGCC

Clustal-W for *Lsi2* genes, CDS. Designed primers are indicated by different colours as shown in Supplementary material S2.

```
TraesCS4B02G361900      AGCCATGGCGCTCGCGTCTCTCCCCAAGGTGGTGCTCGGCTCCGTCGCCTTCGCCATCTT
TraesCS4D02G354900      AGCCATGGCGCTCGCGTCCCTCCCCAAGGTGGTGCTCGGCTCCTTCGCCTTCGCCATCTT
TraesCS5A02G529900      AGCCATGGCGCTCGCGTCTCTCCCCAAGGTGGTGCTCGGCTCCATCGCCTTCGCCGTCTT
P-juncea-Lsi2           AGCCATGGCGCTCGCGTCTCTCCCCAAGGTGGTGCTGGGCTCCTTCGCCTTCGCCATCTT
LOC-Hv123449943         AGCCATGGCGCTCGCGTCTCTCCCCAAGGTGGTGCTGGGCTCCTTCGCCTTCGCCGTCTT
                        ****************** ***************** ****** *********** ****

TraesCS4B02G361900      CTGGATGATGGCCGTGTTCCCGTCGGTGCCCTTCCTGCCCATCGGCCGGACGGCGGGGTC
TraesCS4D02G354900      CTGGATGATGGCCGTGTTCCCGTCGGTGCCCTTCCTGCCCATCGGCCGGACGGCGGGCTC
TraesCS5A02G529900      CTGGATGATGGCGGTGTTCCCGTCGGTGCCCTTCCTGCCCATCGGCCGCACGGCGGGCTC
P-juncea-Lsi2           CTGGATGATGGCGGTGTTCCCGTCGGTGCCCTTCCTGCCCATCGGCCGGACGGCGGGGTC
LOC-Hv123449943         CTGGATGATGGCGGTGTTCCCGTCGGTGCCGTTTCTCCCCATCGGCCGGACGGCGGGGTC
                        ************ ***************** ** ** *********** ******** **

TraesCS4B02G361900      GCTGCTCTCCGCCGTGCTCATGATCGTCTTCCACGTGATCAGCCCCGACGACGCCTACGC
TraesCS4D02G354900      GCTGCTCTCCGCCGTGCTCATGATCGTCTTCCACGTGATCAGCCCCGACGACGCCTACGC
TraesCS5A02G529900      GCTGCTCTCCGCCGTGCTCATGATCGTCTTCCACGTGATCAGCCCCGACGACGCCTACGC
P-juncea-Lsi2           GCTGCTCTCCGCCGTGCTCATGATCGTCTTCCACGTGATCAGCCCCGACGACGCCTACGC
LOC-Hv123449943         GCTGCTCTCCGCCGTGCTCATGATCGTCTTCCACGTGATCAGCCCCGACGACGCCTACGC
                        ************************************************************

TraesCS4B02G361900      CTCCATCGACCTCCCCATCCTCGGCCTGCTCTTCTCCACCATGGTCGTCGGCGGCTACCT
TraesCS4D02G354900      CTCCATCGACCTCCCCATCCTCGGCCTGCTCTTCTCCACCATGGTCGTCGGCGGCTACCT
TraesCS5A02G529900      CTCCATCGACCTCCCCATCCTCGGCCTGCTCTTCTCCACCATGGTCGTCGGTGGCTACCT
P-juncea-Lsi2           CTCCATCGACCTCCCCATCCTCGGCCTGCTCTTCTCCACCATGGTCGTCGGTGGCTACCT
LOC-Hv123449943         CTCCATCGACCTCCCCATCCTCGGCCTGCTCTTCTCCACCATGGTCGTCGGTGGGTACCT
                        *************************************************** ** *****

TraesCS4B02G361900      CAAGAACGCCGGCATGTTCAAGCACCTCGGCACCCTGCTCGCCTGGAAGAGCCAGGGCGG
TraesCS4D02G354900      CAAGAACGCCGGCATGTTCAAGCACCTCGGCACGCTCCTCGCCTGGAAGAGCCAGGGCGG
TraesCS5A02G529900      CAAGAACGCCGGCATGTTCAAGCACCTCGGCACGCTCCTCGCCTGGAAGAGCCAGGGCGG
P-juncea-Lsi2           CAAGAACGCCGGCATGTTCAAGCACCTGGGCACGCTCCTGGCCTGGAAGAGCCAGGGCGG
LOC-Hv123449943         CAAGAACGCCGGCATGTTCAAGCACCTCGGCACCCTCCTCGCGTGGAAGAGCCAGGGCGG
                        *************************** ***** ** ** ** *****************

TraesCS4B02G361900      CCGCGACCTGCTCTGCCGGGTCTGCGTGGTGACCGCGCTCGCCTCCGCGCTCTTCACCAA
TraesCS4D02G354900      CCGCGACCTGCTCTGCCGCGTCTGCGTCGTCACGGCGCTCGCCTCGGCGCTCTTCACCAA
TraesCS5A02G529900      CCGCGACCTGCTCTGCCGCGTCTGCGTCGTCACCGCGCTGGCCTCGGCGCTCTTCACCAA
P-juncea-Lsi2           GCGCGACCTGCTCTGCCGCGTATGCGTCGTCACCGCGCTGGCCTCGGCGCTCTTCACCAA
LOC-Hv123449943         CCGCGACCTGCTCTGCCGCGTCTGCGTCGTCACCGCGCTCGCCTCCGCGCTCTTCACCAA
                         ***************** ** ***** ** ** ***** ***** **************

TraesCS4B02G361900      CGACACCTGCTGCGTCGTGCTCACCGAGTTCGTGCTCGAGCTCGCCGCCGAGCGGAACCT
TraesCS4D02G354900      CGACACCTGCTGCGTCGTGCTCACCGAGTTCGTGCTCGAGCTCGCCGCCGAGCGGAACCT
TraesCS5A02G529900      CGACACCTGCTGCGTCGTGCTCACCGAGTTCGTGCTCGAGCTCGCCGCCGAGCGGAACCT
P-juncea-Lsi2           CGACACCTGCTGCGTCGTGCTCACCGAGTTCGTGCTCGAGCTCGCCGCTGAGCGGAACCT
LOC-Hv123449943         CGACACCTGCTGCGTCGTGCTCACCGAGTTCGTGCTCGAGCTCGCCGCCGAGCGCAACCT
                        ************************************************ ***** *****

TraesCS4B02G361900      TCCGGCCAAGCCCTTCCTCCTGGCCCTCGCCTCCAGCGCCAACATCGGTTCCAGCGCCAC
TraesCS4D02G354900      CCCGGCCAAGCCCTTCCTCCTCGCCCTCGCCTCCAGCGCCAACATCGGCTCCAGCGCCAC
TraesCS5A02G529900      CCCGGCCAAGCCCTTCCTCCTGGCCCTCGCCTCCAGCGCCAACATCGGCTCCAGCGCCAC
P-juncea-Lsi2           CCCGGCCAAGCCCTTCCTCCTCGCGCTCGCCTCCAGCGCCAACATCGGCTCCAGTGCCAC
LOC-Hv123449943         GCCCGCCAAGCCCTTCCTCCTCGCGCTCGCCTCCAGCGCCAACATCGGCTCCAGCGCCAC
                         ** ***************** ** *********************** ***** *****
```

```
TraesCS4B02G361900      CCCCATCGGCAACCCGCAGAACCTGGTCATCGCCTTCAACAGCAAGATCTCGTTTCCAAA
TraesCS4D02G354900      CCCCATCGGCAACCCGCAGAACCTGGTCATCGCCTTCAACAGCAAGATCTCCTTCCCCAG
TraesCS5A02G529900      CCCCATCGGCAACCCGCAGAACCTGGTCATCGCCTTCAACAGCAAGATCTCCTTCCCCAG
P-juncea-Lsi2           CCCCATCGGCAACCCGCAGAATCTGGTCATCGCCTTCAACAGCAAGATCTCCTTCCCCAA
LOC-Hv123449943         CCCCATCGGCAACCCGCAGAACCTCGTCATCGCCTTCAACAGCAAGATCTCCTTCCCCAG
                        ********************* ** ************************** ** ** * 

TraesCS4B02G361900      GTTTTTGATCGGCATCCTGCCGGCCATGCTCGCCGGCATGGCCGTCAACATGGTCATGCT
TraesCS4D02G354900      GTTCCTCATCGGCATCCTGCCGGCCATGCTCGCCGGCATGGCCGTCAACATGGTCATGCT
TraesCS5A02G529900      GTTCCTCATCGGCATCCTGCCGGCCATGCTCGCCGGCATGGCCGTCAACATGGTCATGCT
P-juncea-Lsi2           GTTTTTGATCGGCATCCTGCCAGCCATGCTCGCCGGCATGGCCGTCAACATGGTCATGCT
LOC-Hv123449943         GTTCCTCATCGGGATCCTGCCGGCCATGCTCGCCGGCATGGCCGTCAACATGGTCATGCT
                        ***  * ***** ******** **************************************

TraesCS4B02G361900      GCTCTGCATGTACTGGAAGGACCTGGAGGGCGTGGCCCCCGACGCGGCCGGCAAGCAGAT
TraesCS4D02G354900      GCTCTGCATGTACTGGAAGGACCTGGAGGGCGTGGCCCCCGACGCGGCCGGCAAGCAGAT
TraesCS5A02G529900      GCTCTGCATGTACTGGAAGGACCTCGAGGGTGTGGCCCCCGACGCGGCCGGCAAGCAGAT
P-juncea-Lsi2           GCTCTGCATGTACTGGAAGGACCTTGAGGGCGTCGCCCCCGACGCGGCCGGCAAGCAGAT
LOC-Hv123449943         GCTCTGCATGTACTGGAAGGACCTCGAGGGCGTGGCCCCCGACGCCGACGGCAAGCAGAT
                        ************************ ***** ** *********** * ************

TraesCS4B02G361900      GTCGGTCGTCGAGGAGGGGGGCCGCTCGCCGTCCGTGGCATCGCTCAAGAGCCCGCACCC
TraesCS4D02G354900      GTCGGTCGTCGAGGAGGGCGGCCGCTCGCCGTCCGTGGCGTCGCTCAAGAGCCCGCACCC
TraesCS5A02G529900      GTCGGTCGTCGAGGAGGGCGGCCGCTCGCCGTCCGTGGCGTCGCTCAAGAGCCCGCACCC
P-juncea-Lsi2           GACGGTCGTGGAGGAGG---GCCGCTCGCCATCCGTGGCATCGCACAAGAGCCCGCACCC
LOC-Hv123449943         GGCCGTCGTGGAGGAGGGCGGCCGCTCGCCGTCCGTGGCGTCGCTCAAGAGCCCGCACCC
                        * * ***** *******   ********** ******** **** ***************

TraesCS4B02G361900      GTTCAACGGCACCACGGCCGACGACGGGAACGAGTCGATGATGGAGGAGAACATCTCGAC
TraesCS4D02G354900      GTTCAACGGCACCACCGCCGACGACGGGAACGAGTCGATGATGGAGGAGAACATCTCGAC
TraesCS5A02G529900      GTTCAACGGCACCACGGCCGACGACGGCAACGAGTCGATGATGGAGGAGAACATCTCGAC
P-juncea-Lsi2           GTTCAACGGCACCGTGGCCGACGACGGGGCCGAGTCAATGATGGAGGAGAACATCTCGAC
LOC-Hv123449943         CTTCAACGGCACCATGGCCGACGACGGGAACGAGTCGATGATGGAGGAGAACCTCTCCAC
                         ************   ***********   ****** *************** **** **

TraesCS4B02G361900      CAAGCACCCGTGGTTCATGCAGTGCACGGAGCACCGGCGCAAGCTGTTCCTCAAGAGCTT
TraesCS4D02G354900      CAAGCACCCGTGGTTCATGCAGTGCACGGAGCACCGGCGCAAGCTGTTCCTCAAGAGCTT
TraesCS5A02G529900      CAAGCACCCGTGGTTCATGCAGTGCACGGAGCACCGACGCAAGCTGTTCCTCAAGAGCTT
P-juncea-Lsi2           CAAGCACCCATGGTTCATGCAGTGCACGGAGCACCGGCGCAAGCTGTTCCTCAAGAGCTT
LOC-Hv123449943         CAAGCACCCCTGGTTCATGCAGTGCACGGAGCACCGGCGCAAGCTCTTCCTCAAGAGCTT
                        ********* ************************** ******** **************

TraesCS4B02G361900      CGCCTACATCGTGACGCTGGGCATGGTGGTAGCATACATGGCCGGGCTCAACATGTCGTG
TraesCS4D02G354900      TGCCTACATCGTGACGCTGGGCATGGTGGTGGCATACATGGCCGGGCTCAACATGTCGTG
TraesCS5A02G529900      CGCCTACATCGTGACGCTGGGCATGGTGGTGGCCTACATGGCTGGGCTCAACATGTCGTG
P-juncea-Lsi2           CGCCTACATCGTGACGGTGGGCATGGTGGTGGCCTACATGGCCGGGCTCAACATGTCGTG
LOC-Hv123449943         CGCCTACATCGTGACGGTGGGCATGGTGGTGGCCTACATGGCCGGGCTCAACATGTCGTG
                         *************** ************* ** ******** *****************

TraesCS4B02G361900      GACGGCCATCACCACGGCCATCGCGCTGGTCGTCGTCGACTTCCGGGACGCCGAGCCGTG
TraesCS4D02G354900      GACGGCCATCACCACCGCCATCGCGCTCGTCGTCGTCGACTTCCGGGACGCCGAGCCTTG
TraesCS5A02G529900      GACGGCCATCACCACCGCCATCGCGCTGGTCGTCGTCGACTTCCGGGACGCCGAGCCGTG
P-juncea-Lsi2           GACGGCCATCACCACCGCCATCGCGCTCGTCGTCGTCGACTTCCGGGACGCCGAGCCCTG
LOC-Hv123449943         GACGGCCATCACCACCGCCATCGCGCTGGTTGTCGTAGACTTCCGGGACGCCGAGCCCTG
                        *************** *********** ** ***** ******************** **

TraesCS4B02G361900      CCTCGTCAAGGTCTCCTACTCGCTGCTCGTCTTCTTCTCCGGCATGTTCATCACGGTGAG
TraesCS4D02G354900      CCTCGTCAAGGTCTCCTACTCGCTGCTCGTCTTCTTCTCCGGCATGTTCATCACGGTGAG
TraesCS5A02G529900      CCTCGTCAAGGTCTCCTACTCGCTGCTCGTCTTCTTCTCCGGCATGTTCATCACGGTGAG
P-juncea-Lsi2           CCTCGTCAAGGTCTCCTACTCGCTGCTCGTCTTCTTCTCCGGCATGTTCATCACGGTGAG
LOC-Hv123449943         CCTCGTCAAGGTCTCCTACTCGCTGCTCGTCTTCTTCTCCGGCATGTTCATCACCGTCAG
                        ****************************************************** ** **
```

```
TraesCS4B02G361900        CGGGTTCAACAAGACGGGGCTGCCGGGCGCCATCTGGAACTTCATGGCGCCCTACTCCAA
TraesCS4D02G354900        TGGGTTCAACAAGACGGGGCTGCCGGGCGCCATCTGGAACTTCATGGCGCCCTACTCCAA
TraesCS5A02G529900        CGGGTTCAACAAGACGGGGCTGCCGGGCGCCATCTGGAACTTCATGGCGCCCTACTCCAA
P-juncea-Lsi2             TGGGTTCAACAAGACGGGGCTGCCGGGCGCCATCTGGAACTTCATGGCGCCCTACTCCAA
LOC-Hv123449943           CGGCTTCAACAAGACGGGGCTGCCGGGCGCCATCTGGAACTTCATGGCGCCCTACTCCAA
                           ** ********************************************************

TraesCS4B02G361900        GGTGGACAGCGCCGGCGGCATCTCCGTGCTCTCCGTCATCATCCTCCTCCTCTCCAACCT
TraesCS4D02G354900        GGTGGACAGCGCCGGCGGCATCTCCGTGCTCTCCGTCATCATCCTCCTCCTCTCCAACCT
TraesCS5A02G529900        GGTGGACAGCGCCGGCGGCATCTCCGTGCTCTCCGTCATCATCCTCCTCCTCTCCAACCT
P-juncea-Lsi2             GGTCGACAGCGCCGGCGGCATCTCCGTGCTCTCCGTCATCATCCTCCTCCTCTCCAACCT
LOC-Hv123449943           GGTGGACAGCGCCGGCGGCATCTCCGTGCTCTCCGTCATCATCCTCCTCCTCTCCAACCT
                          *** ********************************************************

TraesCS4B02G361900        CGCCTCCAACGTACCAACAGTGCTGCTGATGGGGAACGAGGTGGCGACCGCGGCGGCTCT
TraesCS4D02G354900        CGCCTCCAACGTACCAACAGTGCTACTGATGGGGAACGAGGTGGCGACCGCGGCGGCTCT
TraesCS5A02G529900        CGCCTCCAACGTACCAACAGTGCTGCTGATGGGGAACGAGGTGGCGACCGCAGCGGCCCT
P-juncea-Lsi2             CGCATCCAACGTACCCACAGTGCTGCTGATGGGGAACGAGGTGGCCACCGCGGCGGCGCT
LOC-Hv123449943           CGCCTCCAACGTACCAACAGTGCTGCTGATGGGGAACGAGGTGGCTACCGCGGCGGCGCT
                          *** *********** ******** ******************** ***** ***** **

TraesCS4B02G361900        GATCTCCCCGGCGGCGGTGACTCGGTCGTGGCTGCTGCTGGCGTGGGTGAGCACGGTGGC
TraesCS4D02G354900        GATCTCCCTGGCGGCGGTGACTCGGTCGTGGCTGCTGTTGGCGTGGGTGAGCACGGTGGC
TraesCS5A02G529900        GATCTCGCCGGCAGCGGTGACGCGGTCGTGGCTGCTGCTGGCGTGGGTGAGCACGGTGGC
P-juncea-Lsi2             GATCTCGCCGGCAGCGGTGACGCGGTCGTGGCTGCTGCTAGCGTGGGTGAGCACGGTGGC
LOC-Hv123449943           CATCTCGCCGGCAGCGGTGACGCGGTCCTGGCTGTTGCTGGCGTGGGTGAGCACGGTGGC
                           ***** * *** ******** ***** ****** ** * ********************

TraesCS4B02G361900        GGGCAACCTTTCGCTGCTGGGGTCGGCGGCGAACCTGATCGTGTGCGAGCAGGCGCGCCG
TraesCS4D02G354900        GGGCAACCTGTCGCTGCTGGGGTCGGCGGCGAACCTGATCGTGTGCGAGCAGGCGCGCCG
TraesCS5A02G529900        AGGGAATCTCTCGCTGCTGGGGTCGGCGGCGAACCTGATCGTGTGTGAGCAGGCGCGTCG
P-juncea-Lsi2             GGGCAACCTGTCGCTGCTGGGGTCGGCGGCGAACCTGATCGTGTGTGAGCAGGCGCGCCG
LOC-Hv123449943           GGGCAACCTGTCGCTGCTAGGGTCAGCAGCGAACCTGATCGTGTGCGAGCAGGCACGCCG
                           ** ** ** ******** ***** ** ***************** ******** ** **

TraesCS4B02G361900        GGCGCCGCGCAACGCCTACGAGCTCACCTTCTGGAACCACCTCATCTTCGGCGTGCCCTC
TraesCS4D02G354900        GGCGCCGCGCAACGCCTACGAGCTCACCTTCTGGAACCACCTCATCTTCGGCGTGCCCTC
TraesCS5A02G529900        GGCGCCGCGTAACGCCTATGAGCTCACCTTCTGGAACCACCTCATCTTTGGCGTGCCCTC
P-juncea-Lsi2             GGCGCCGCGGAACGCCTACGAGCTCACCTTCTGGAACCACCTCGTCTTTGGCGTGCCCTC
LOC-Hv123449943           GGCGCCCCGCAACGCCTACGAGCTCACCTTCTGGAACCACCTCATCTTCGGCGTGCCCTC
                          ****** ** ******** ************************ **** ***********

TraesCS4B02G361900        CACCCTCATCGTCACCGCCGTCGGCATCCCCCTCATCGGAAAGATGTAGCCACC
TraesCS4D02G354900        CACCCTCATCGTCACCGCCGTCGGCATCCCCCTCATCGGAAAGATGTAGCCACC
TraesCS5A02G529900        CACCCTCATCGTCACCGCCGTCGGCATCCCCCTCATCGGAAAGATGTAGCCACC
P-juncea-Lsi2             CACCCTCATCGTCACCGCCGTCGGCATCCCCCTCATCGGAAAGATGTAGCCGCC
LOC-Hv123449943           CACCCTCATCGTCACCGCCGTCGGCATCCCCCTCATCGGAAAGATGTAGCCACC
                          *************************************************** **
```

Lsi3

In CDS sequences, 'Start'- and 'Stop'-codons are highlighted by green and red, respectively. Exons are indicated by different colours on example of *P. juncea* sequence.

>Lsi3-Os10g39980

GGCCATGGCGCTGGCGTCGCTGCCGAAGGTGGTGATGGGGTCGGTGGCGTTCGGTGTGTT
CTGGATGCTGGCGGTGTTCCCGTCGGTGCCGTTCCTGCCGATCGGGCGGACGGCGGGGGC
GCTGCTGGGCGCGGTGCTGATGATCGTGTTCCACGTGATCAGCGCCGACGACGCCTACGC
CTCCATCGACCTCCCCATCCTGGGCCTCCTCTTCGCCACCATGGTCGTCGGCGGCTACCT
CAAGAACGCCGGCATGTTCAGGCACCTGGGCCGGCTGCTGGCGTGGCGGAGCCAGGGCGG
GCGCGACCTCATGTGCCGCGTCTGCGTCGTCACCGCGCTCGCCAGCGCGCTCTTCACCAA
CGACACCTGCTGCGTCGTCCTCACCGAGTTCGTGCTCGAGCTCGCCGCCGAGCGCAACCT
CCCGGCGAAGCCGTTCCTGCTCGCGCTCGCCACCAGCGCCAACATCGGCTCCAGCGCGAC
GCCCATCGGCAACCCGCAGAACCTGGTCATCGCCTTCAACAGCAAGATCTCCTTTATTTC
CTTCCTCCTCGGCATCCTCCCCGCCATGCTCGCCGGCATGGGGATCAACATGCTCATGCT
GCTCTGCATGTACTGGAAGGAGCTCGACGGCGGCGCCTGCAGCCCCGACGAGGTCGCCGC
CGGCAAGCAGATGGAGGCCATCGAGGAGGGCCGCCGCACGGCGCTCAACAACAACAAGAA
AGACGACGGCGACGCCGCCACCCCCGCATCGCCGGAGGACGACGACGGCGGCGACGCCGA
GTCGATGATGTCGGAGAACATCTCGACGAAGCACCGGTGGTTCATGCAGTGCTCGGAGCA
CCGGCGGAAGCTGTTCCTCAAGAGCTTCGCGTACGTGGTCACCGTCGGCATGCTCGTCGC
CTACATGCTCGGCCTCAACATGTCGTGGACCGCCATCACGACCGCCATCGCCCTCGTCGT
CGTCGACTTCCGCGACGCCGAGCCATGCCTCGACAAGGTGTCGTACTCGCTGCTGGTGTT
CTTCTCGGGGATGTTCGTGACGGTGAGCGGGTTCAACAAGACGGGGCTACCCGGGGCCAT
CTGGAACGTGATGGCGCCCTACTCAAAGATCAACCACGTCACCGGCGTCACCGTCCTCTC
CGTCATCATCCTCCTCCTCTCCAACCTCGCCTCCAACGTCCCCACCGTGTTGTTGATGGG
GGATGAGGTGGCGGCGGCGGCGGCGACGATATCACCGGCGGCGGTGACGCGGTCGTGGCT
GCTGCTGGCGTGGGTGAGCACGGTGGCGGGGAACCTGTCGCTGCTGGGGTCGGCGGCGAA
CCTGATCGTGTGCGAGCAGGCGAGGCGGGCGACGCGCAACGCCTACGACCTCACCTTCTG
GAACCACGTCATCTTCGGACTCCCATCCACCCTCGTCGTCACCGCCATCGGCATCCCTCT
CATCGGCAAGATCAACATCTAGTATTC

>Lsi3-Bradi3g32730

CGATATGTCGCAGCTGGCGTCTCTGCCCAAGGTGATCCTAGGCTCGGCATCCTTCGGCGT
ATTCTGGGTCCTCGCCGTGTTCCCCTCCGTCCCGTTCCTCCCCATCGGGCGCACGGCGGG
GGCCCTGCTGAGCGCGGCCCTGATGATCCTCTTCCACGTCATCAACCCGGACGACGCCTA
CGCGTCCATCGACCTGCCCATCCTGGGCCTCCTCTTCGCCACCATGGTGGTGGGGGGTTA
CCTGAAGCAGGCCGGCATGTTCGCCCACCTGGGCCGGCTCCTGGCCTGGCGCTGCCGGCC
TGGGGGCCGCGACCTGCTCTGCCGCGTCTGCGTCGTCACGGCGCTCGCCTCCGCGCTCTT
CACCAACGACACCTGCTGCGTCGTGCTAACCGAGTTCGTGCTCCAGCTCGCCGCCGACAG
GAAGCTCCCCGCCAAGCCCTTCTTGCTGGCGCTGGCCACGAGTGCCAATATTGGGTCTAG
TGCGACGCCCATCGGGAACCCGCAGAATCTGGTCATCGCGTTTAGCAGCAAGATTTCCTT
CGTGGGGTTTTTGCTTGGCGTCTTGCCGGCCATGCTCGCCGGCATGGCTGTCAATATGGT
GCTGCTCCTCTGCATGTACTGGAAGGACCTCGCCCCCGGCGACATGGCTGCCGGGAATAG
TAAGGAGATGGAGATGGAGGATGTCGTGGTCGTCGAGGAGGGCTCGGGCTTGCCATCGCC
ACCGTCGACGTTGGTGGCCACCGGCGCCGGTGAAGAAGACGACCAGGAGAGCAGCATCTC
GACGAAGCACAGGTGGTTCATGGAGTGCACCGAGCGGCGGCGGAGGGTGTTCCTGAAGAG

CTTCGCGTACGTGGCGACGGCGGGGATGGTGGTGGCGTACATGATGGGGCTCAACATGTC
GTGGACGGCCATCACCACCGCCGTGGCGCTCGTCGTCGCCGACTTCCGGGACGCGGAGCG
CTGCCTGGGGAAGGTGTCCTACTCGCTGCTCGTCTTCTTCTCCGGGATGTTCGTCACGGT
CAGCGGGTTCAACAAGACGGGGCTCCCGGGGGCCATCTGGAACGTCGTGGCGCCCTACTC
TAGGATTGATCATGTCAGCGGCGTCACCGTGCTGTCGCTCATCATACTTCTCCTCTCCAA
CCTCGCCTCCAACGTGCCTACTGTGCTGCTGATGGGGGACGAGGTGGCGGCGTCGGCGGC
GACGATCTCGGAGGCGGCGGTGAGACGGTCGTGGCTGCTGCTGGCGTGGGTGAGCACGGT
GGCGGGGAACCTGTCGCTGCTGGGATCGGCGGCGAACCTGATCGTGTGCGAGCAAGCGCG
CCGGGCGCCGCGCAACGCCCACGACCTCACCTTCTGGAGCCACGTCGTCTTCGGCGCCCC
CTCCACGCTGCTCGTCACCGCCGTCGGCATACCGCTCATCGGCATCCTCAACACCGCCTA
GCTGGC

>Lsi3-Hv123411373

TAGCATGGCGCTGGCGTCCTTGTCCAAGGTGGTGCTGGGATCGGCGGCGTTCGGGGTGTT
CTGGGTGCTGGCCGTGTTCCCGTCGGTGCCTTTCATGCCTATCGGCCGCACGGCGGGCGC
GCTGCTGGGGGCGGCGCTGATGGTCGTCTTCCACGTGATCAGCCCGGATGACGCCTACGC
CTCCGTGGACCTCCCCATCCTGGGCCTCCTCTTTGCCACCATGGTCGTCGGCGGCTACCT
CAAGAGCGTCGGCATGTTCGGGCACCTGGGCCGGCTGCTGGCGTGGCGGAGCCAGGGCGG
GCGCGACCTCCTGTGCCGCGTCTGCGTCGTCACGGCACTCGCCAGCGCGCTCTTCACCAA
CGACACCTGCTGCGTCGTGCTCACGGAGTTCGTGCTCGAGCTCGCCGCCGAGCGCAACCT
CCCCGCGAAGCCGTTCCTGCTGGCGCTGGCCACCAGCGCCAACATCGGGTCCAGCGCCAC
GCCCATCGGGAACCCGCAGAACCTGGTCATCGCCTTCAACAGCAAGATAACCTTCGTGGG
ATTCTTCCTCGGCATCCTGCCGGCCATGGTCGCAGGCATGGCCGTCAACATGGTCATGCT
GCTCTGCATGTACTGGAAGGACCTCGAGGGGAACAGTCCCGACGCGATAGCCGCCGAGAA
GGAGATGGCGGCAGTTGAGGAGGGTCGCCGGCCGTCCCCGGCGACGTCGGGGACGCTCAA
GAGCCCCGGCCAGACGATGCTGGTTCAGTCGTCATCGCTGGTCGCGGCCGCCGACCACGA
CTCGGTGATGGTGGAGAGCATCTCGACCAAGCACCGGTGGTTCATACAGTGCTCGGCGCC
GCAGCGGAGGCTGTTCCTCAAGAGCTTCGCGTACATCGTGACCGCCGGCATGCTGGTCGC
CTACATGCTGGGGCTCAACATGTCGTGGACCGCCATCACCACCGCCGTCGCCCTCATCGT
CGTGGACTTCCGCGACGCCGAGCTCTGCCTCGGCAAGGTGTCTTACTCGCTGCTGGTTTT
CTTCACGGGGATGTTCGTGACGGTGAGCGGGTTCAATAAGACGGGGCTCCCTGGCGCCAT
CTGGAACGTCATGGCGCCATACTCCAAGATCAGCCACGTCAGCGGCGTCACCGTGCTCTC
CATCATCATCATTATCCTCTCCAACCTAGCCTCCAACGTACCGACCGTGCTGCTGATGGG
TGACGAGGTGGCGGCATCGGCAGCGACGATCTCGGCGGCGGCAGTGACGCGGTCGTGGCT
GCTGCTGGCGTGGGTCAGCACGGTGGCGGGTAACCTGTCCCTGCTGGGGTCAGCGGCGAA
CCTCATCGTGTGCGAGCAGGCACGCCGGGCGCCGCGCAACGCACACGACCTCACGTTCTG
GAGCCACGTCGTCTTCGGCGCACCATCCACGCTCGTCGTCACCGCCGTCGGCATACCCCT
CATCGGCATCATCAACGCTGCCTAGATTCA

>Lsi3-TaCS1A02G207300

TAGCATGGCGCTGGCGTCTTTGTCCAAGGTGGTGCTGGGGTCGGCGGCGTTCGGGGTATT
CTGGGTGCTGGCCGTGTTCCCGTCGGTCCCTTTCCTGCCGATCGGGCGCACGGCGGGTGC
GCTGCTGGGGGCTGCTCTGATGGTCGTCTTCCACGTGATCAGCCCGGACGACGCCTACGC
GTCCGTGGACCTCCCCATCCTGGGCCTCCTCTTTGCCACCATGGTCGTCGGCGGCTACCT
CAAGAGCGCCGGCATGTTCGGGCACCTTGGCCGGCTGCTGGCGTGGCGGAGCCAGGGCGG
GCGCGACCTGCTGTGCCGCATCTGCGTCGTCACGGCCCTCGCCAGCGCGCTCTTCACCAA
CGACACCTGCTGCGTCGTGCTCACGGAGTTCGTGCTCGAACTCGCCGCCGAGCGCAACCT

CCCCGCGAAGCCGTTCCTGCTGGCGCTGGCCACCAGCGCCAACATCGGGTCCAGCGCCAC
GCCCATCGGGAACCCGCAGAACCTGGTCATCGCCTTCAACAGTAAGATCTCATTCGTGGG
CTTCCTCCTCGGCATCCTGCCGGCCATGCTCGTTGGTATGGCCGTCAACATGGTCATGCT
GCTCTGCATGTACTGGAAGGACCTCGAGGGGAGCAGTCCCGACGCGGTGGCCGCCGAGAA
GGAGATGGCGGCAGTCGAGGAGGGTCGCCGGCCGTCCCCGGCGACGTCGACGACGCTCAA
GAGCCCCGGCCGGACGATGCTGGTCCAGTCCTCGTCGCTGGTCGCCGGCGCCGACCACGA
GTCGGTGATGGTGGAGAGCATCTCGACCAAGCACCGGTGGTTCATACAGTGCTCGGCGCC
GCAGCGGAGGCTGTTCCTCAAGAGCTTCGCGTACGTCGTGACCGCCGGCATGCTGGTCGC
CTACATGCTGGGGCTCAACATGTCGTGGACGGCCATCACCACCGCCGTCGCCCTCATCGT
GGTGGACTTCCGCGACGCCGAGCTCTGCCTCGGCAAGGTGTCTTACTCGTTGCTGGTTTT
CTTCACGGGAATGTTCGTGACCGTGAGCGGGTTCAACAAGACGGGGCTCCCCGGCGCCAT
CTGGAAAGTCATGGAGCCATACTCCAAGATCAACCACGTGAGGGGCATCACAGTGCTCTC
CCTCATCGTCATCGTCCTCTCCAACCTTGCCTCCAACGTACCAACCGTGCTGCTGATGGG
TGACGAGGTGGCGGCGTCGGCAGCGACGATCTCGACGGCGGCGGTGACGCGGTCGTGGCT
GCTGCTGGCGTGGGTGAGCACGGTGGCGGGTAACCTGTCCCTGCTGGGGTCGGCGGCGAA
CCTCATCGTGTGCGAGCAGGCACGCCGGGCGCCGCGCAACGCGCACGACCTCACCTTCTG
GAGCCACGTCGTCTTCGGCGCGCCGTCCACGCTCGTCGTCACCGCCGTCGGCATACCCCT
CATTGGCATCATCAACGCTGCCTAGTTCGA

>Lsi3-TaCS1B02G221100

TAGCATGGCGCTGGCGTCTTTGTCCAAGGTGGTGCTGGGGTCGGCGGCGTTCGGGGTGTT
CTGGGTGCTGGCCGTGTTCCCGTCGGTGCCTTTCCTGCCGATCGGCCGCACGGCGGGTGC
GCTGCTGGGGGCGGCGCTGATGGTCGTCTTCCACGTGATCAGCCCGGATGACGCCTACGC
CTCCGTGGACCTCCCCATCCTGGGCCTCCTCTTTGCCACCATGGTCGTCGGCGGCTACCT
CAAGAGCGCCGGCATGTTCGGGCACCTTGGCCGGCTGCTGGCGTGGCGGAGCCAGGGCGG
GCGCGACCTGCTTTGCCGCGTCTGCGTCGTCACGGCCCTCGCCAGCGCGCTCTTCACCAA
CGACACCTGCTGCGTCGTGCTCACGGAGTTCGTGCTCGAGCTCGCCGCCGAGCGCAACCT
CCCCGCGAAGCCGTTCCTGCTGGCGCTGGCCACCAGCGCCAATATCGGGTCCAGCGCCAC
GCCCATCGGGAACCCGCAGAACCTGGTCATCGCCTTCAACAGCAAGATCTCTTTCGTGGG
CTTCTTCCTCGGCATCCTGCCGGCCATGCTCGCAGGTATGGCCGTCAACATGGTCATGCT
GCTCTGCATGTACTGGAAGGACCTCGAGGGGAGCAGTCCCGACGAGGTGGCCGCCGAGAA
GGAGATGGCGGCCGTCGAGGAGGGTCGCCTGCCGTCCCCGGCAACGTCGACGACGCTCAA
GGGCCCCGGCCAGACGATGCTGGTCCAGTCGTCATCGCTGGTCGCCGCCGCCGGCCACGA
CTCGGTGATGATGGAGAGCATCTCGACCAAGCACCGGTGGTTCATACAGTGCTCGGCGCC
GCAGCGGAGGCTGTTCCTCAAGAGCTTCGCGTACATCGTGACCGCCGGCATGCTGGTCGC
CTACATGCTCGGGCTCAACATGTCGTGGACGGCCATCACCACCGCCGTCGCGCTCATCGT
GGTGGACTTCCGCGACGCGGAGCTCTGCCTCGGCAAGGTGTCTTACTCGCTGCTGGTTTT
CTTCACGGGGATGTTCGTGACGGTGAGCGGGTTCAATAAGACGGGGCTCCCTGGTGCCAT
CTGGAACGTCATGGAGCCATACTCCAAGATCAACCACGTCAGGGGCATCACCGTGCTCTC
CCTCATCGTCATCATCCTCTCCAACCTAGCCTCCAACGTACCAACCGTGCTCCTGATGGG
TGACGAGGTGGCGGCGTCGGCAGCGACGATCTCGGCGGCGGCGGTGACGCGGTCGTGGCT
GCTGCTGGCGTGGGTGAGCACGGTGGCGGGTAACCTGTCCCTGCTGGGGTCGGCGGCGAA
CCTCATCGTGTGCGAGCAGGCACGCCGGGCGCCGCGCAACGCACACGACCTCACCTTCTG
GAGCCACGTCGTCTTCGGCGCGCCGTCCACGCTCGTTGTCACCGCCGTCGGCATACCACT
CATCGGCATCATCAACGCTGCCTAGATCGA

>Lsi3-TaCS1D02G210500

TAGCATGGCGCTGGCGTCTTTGTCCAAGGTGGTGCTGGGGTCGGCGGCGTTCGGGGTGTT
CTGGGTGCTGGCCGTGTTCCCGTCGGTGCCTTTCCTGCCGATCGGCCGCACGGCGGGTGC
GCTGCTGGGGGCGGCGCTGATGGTCGTCTTCCACGTGATCAGCCCGGATGACGCCTACGC
CTCCGTGGACCTCCCCATCCTGGGCCTCCTCTTTGCCACCATGGTGGTCGGCGGCTACCT
CAAGAGCGCCGGCATGTTCGGGCACCTTGGCCGGCTGCTGGCGTGGCGGAGCCAGGGCGG
GCGCGACCTGCTGTGCCGCGTCTGCGTCGTCACGGCCCTCGCCAGCGCGCTCTTCACCAA
CGACACCTGCTGCGTGGTGCTCACGGAGTTCGTGCTCGAGCTCGCCGCCGAGCGCAACCT
CCCCGCGAAGCCGTTCCTGCTGGCGCTGGCCACCAGCGCCAATATCGGGTCCAGCGCCAC
GCCCATCGGGAACCCGCAGAACCTGGTCATCGCCTTCAACAGCAAGATCTCCTTCGTGGG
CTTCTTCCTCGGCATCCTACCGGCCATGCTCGCAGGCATGGCCGTCAACATGGTCATGCT
GCTCTGCATGTACTGGAAGGACCTCGAGGGGAGCAGTCCCGACGCGGTGGCCGCCGAGAA
GGAGATGGCGGCAGTCGAGGAGGGTCGCCGGCCGTCCCCGGCAACGTCGACGACGCTCAA
GAGCCCCGGCCAGACGATGCTGGTCCAGTCGCCGTCGCTGGTCGCGGCCGCCGACCACGA
CTCGGTGATGGTGGAGAGCATCTCGACCAAGCACCGGTGGTTCATACAGTGCTCGGCGCC
GCAGCGGAGGCTGTTCCTCAAGAGCTTCGCGTACATCGTGACCGCCGGCATGCTGGTCGC
CTACATGCTCGGGCTCAACATGTCGTGGACCGCCATCACCACCGCCGTCGCGCTCATCGT
GGTGGACTTCCGCGACGCCGAGCTCTGCCTCGGCAAGGTGTCTTACTCGCTGCTGGTTTT
CTTCACGGGGATGTTCGTGACGGTGAGCGGGTTCAATAAGACGGGGCTCCCTGGCGCCAT
CTGGAAAGTCATGGAGCCATACTCCAAGATCAACCACGTCAGGGGCATCACCGTGCTCTC
CCTCATCGTCATCATCCTCTCCAACCTAGCCTCCAACGTACCAACCGTGCTGCTGATGGG
TGACGAGGTGGCGGCGTCGGCGGCGACGATCTCGGCGGCGGCGGTGACGCGGTCGTGGCT
GCTGCTGGCGTGGGTGAGCACGGTGGCGGGTAACCTGTCCCTGCTGGGGTCGGCGGCGAA
CCTCATCGTGTGCGAGCAGGCACGCCGGGCGCCGCGCAACGCACACGACCTCACCTTCTG
GAGCCACGTCGTCTTCGGCGCGCCGTCCACGCTCGTCGTCACCGCCGTCGGCATACCCCT
CATCGGCATCATCAACGCTGCCTAGATCGA

>PjLsi3

TAGCATGGCGCTGGCGTCTCTGTCCAAGGTGGTGATGGGGTCGGCGGCGTTCGGGGTGTT
CTGGGTGCTGGCCGTGTTCCCGTCGGTGCCTTTCCTGCCGATCGGCCGCACGGCGGGTGC
GCTGCTGGGGGCTGCGCTGATGGTCGTCTTCCACGTGATCAGCCCGGATGACGCCTACGC
CTCCGTGGACCTCCCCATCCTGGGCCTCCTCTTTGCCACCAAGGTCGTCGGCGGCTACCT
CAAGAGCGCCGGCATGTTCGGGCACCTGGGCCGGCTGCTGGCGTGGCGGAGCCAGGGCGG
GCGCGACCTCCTGTGCCGCGTCTGCGTCGTCACGGCCCTCGCCAGCGCGCTCTTCACCAA
CGACACCTGCTGCGTCGTGCTCACGGAGTTCGTGCTCGAGCTCGCAGACGAGCGCAACCT
ACCCGCGAAGCCGTTCCTGCTGGCGCTGGCCACCAGCGCCAACATCGGGTCCAGCGCCAC
GCCCATCGGGAACCCGCAGAACCTGGTTATCGCCTTCAACACCAAGATCTCCTTCGTGGG
CTTCTTCCTCGGCATCCTGCCGGCCATGCTCGCCGGCATGGCCGTCAACATGGCCATGCT
GCTCTGCATGTACTGGAAGGACCTCGAGGGGAACAGTCCCGACGCGGTGGCCGCCGAGAA
GGAGATGGCGGCCGTCGAGGAGGGTCGCCGGCCGTCCCCGGCAACGTCGACGACGCTCAA
GAGCCCCGCCCAGACGATGTTGGTCCAGTCGTCGTCGCTGGTCGCGGCCGCCGACCTCGA
CTCGGCGATGGCGGAGAGCATCTCGACCAAGCACCGGTGGTTCATACAGTGCTCGGCGCC
GCAGCGGAGGCTGTTCCTCAAGAGCTTCGCGTACATCGTGACCGCCGGCATGCTGGTCGC
CTACATGCTGGGGCTCAACATGTCGTGGACCGCCATCACCACCGCCGTCGCCCTCATCGT
CGTGGACTTCCGCGACGCCGAGCTCTGCCTCGGCAAGGTGTCTTACTCGCTGCTGGTTTT
CTTCACGGGGATGTTCGTGACGGTGAGCGGGTTCAATAAGACGGGGCTCCCTGGCGCCAT
CTGGAACGTCATGGCGCCATACTCCAAGATCAACCACGTCAGCGGCGTCACCGTGCTCTC

CCTCATCATCCTCCTCCTCTCCAACCTTGCCTCCAACGTACCAACCGTGCTGATGGGTGA
CGAGGTGGCGGCGTCGGCAGCGACCATCTCGGCGGCGGCGGTGACGCGGTCGTGGCTGCT
GCTGGCGTGGGTGAGCACGGTGGCGGGTAACCTGTCCCTGCTGGGGTCGGCGGCGAACCT
CATCGTGTGCGAGCAGGCGCGCCGGGCGCCGCGCAACGCGCATGACCTCACCTTCTGGAG
CCACGTCGTCTTCGGCGCACCGTCCACGCTCGTCGTCACCGCCGTCGGCATACCCCTCAT
CGGCATCATCAACGCTGCCTGAATCCA

Clustal-W for *Lsi3* genes, CDS. Designed primers are indicated by different colours as shown in Supplementary material S2.

```
TraesCS1B02G221100      TAGCATGGCGCTGGCGTCTTTGTCCAAGGTGGTGCTGGGGTCGGCGGCGTTCGGGGTGTT
TraesCS1D02G210500      TAGCATGGCGCTGGCGTCTTTGTCCAAGGTGGTGCTGGGGTCGGCGGCGTTCGGGGTGTT
TraesCS1A02G207300      TAGCATGGCGCTGGCGTCTTTGTCCAAGGTGGTGCTGGGGTCGGCGGCGTTCGGGGTATT
P-juncea-Lsi3           TAGCATGGCGCTGGCGTCTCTGTCCAAGGTGGTGATGGGGTCGGCGGCGTTCGGGGTGTT
LOC-Hv123411373         TAGCATGGCGCTGGCGTCCTTGTCCAAGGTGGTGCTGGGATCGGCGGCGTTCGGGGTGTT
                        ******************  ************** **** ***************** **

TraesCS1B02G221100      CTGGGTGCTGGCCGTGTTCCCGTCGGTGCCTTTCCTGCCGATCGGCCGCACGGCGGGTGC
TraesCS1D02G210500      CTGGGTGCTGGCCGTGTTCCCGTCGGTGCCTTTCCTGCCGATCGGCCGCACGGCGGGTGC
TraesCS1A02G207300      CTGGGTGCTGGCCGTGTTCCCGTCGGTCCCTTTCCTGCCGATCGGGCGCACGGCGGGTGC
P-juncea-Lsi3           CTGGGTGCTGGCCGTGTTCCCGTCGGTGCCTTTCCTGCCGATCGGCCGCACGGCGGGTGC
LOC-Hv123411373         CTGGGTGCTGGCCGTGTTCCCGTCGGTGCCTTTCATGCCTATCGGCCGCACGGCGGGCGC
                        *************************** ****** **** ***** *********** **

TraesCS1B02G221100      GCTGCTGGGGGCGGCGCTGATGGTCGTCTTCCACGTGATCAGCCCGGATGACGCCTACGC
TraesCS1D02G210500      GCTGCTGGGGGCGGCGCTGATGGTCGTCTTCCACGTGATCAGCCCGGATGACGCCTACGC
TraesCS1A02G207300      GCTGCTGGGGGCTGCTCTGATGGTCGTCTTCCACGTGATCAGCCCGGACGACGCCTACGC
P-juncea-Lsi3           GCTGCTGGGGGCTGCGCTGATGGTCGTCTTCCACGTGATCAGCCCGGATGACGCCTACGC
LOC-Hv123411373         GCTGCTGGGGGCGGCGCTGATGGTCGTCTTCCACGTGATCAGCCCGGATGACGCCTACGC
                        ************ ** ********************************* **********

TraesCS1B02G221100      CTCCGTGGACCTCCCCATCCTGGGCCTCCTCTTTGCCACCATGGTCGTCGGCGGCTACCT
TraesCS1D02G210500      CTCCGTGGACCTCCCCATCCTGGGCCTCCTCTTTGCCACCATGGTGGTCGGCGGCTACCT
TraesCS1A02G207300      GTCCGTGGACCTCCCCATCCTGGGCCTCCTCTTTGCCACCATGGTCGTCGGCGGCTACCT
P-juncea-Lsi3           CTCCGTGGACCTCCCCATCCTGGGCCTCCTCTTTGCCACCAAGGTCGTCGGCGGCTACCT
LOC-Hv123411373         CTCCGTGGACCTCCCCATCCTGGGCCTCCTCTTTGCCACCATGGTCGTCGGCGGCTACCT
                         **************************************** *** **************

TraesCS1B02G221100      CAAGAGCGCCGGCATGTTCGGGCACCTTGGCCGGCTGCTGGCGTGGCGGAGCCAGGGCGG
TraesCS1D02G210500      CAAGAGCGCCGGCATGTTCGGGCACCTTGGCCGGCTGCTGGCGTGGCGGAGCCAGGGCGG
TraesCS1A02G207300      CAAGAGCGCCGGCATGTTCGGGCACCTTGGCCGGCTGCTGGCGTGGCGGAGCCAGGGCGG
P-juncea-Lsi3           CAAGAGCGCCGGCATGTTCGGGCACCTGGGCCGGCTGCTGGCGTGGCGGAGCCAGGGCGG
LOC-Hv123411373         CAAGAGCGTCGGCATGTTCGGGCACCTGGGCCGGCTGCTGGCGTGGCGGAGCCAGGGCGG
                        ******** ****************** ********************************

TraesCS1B02G221100      GCGCGACCTGCTTTGCCGCGTCTGCGTCGTCACGGCCCTCGCCAGCGCGCTCTTCACCAA
TraesCS1D02G210500      GCGCGACCTGCTGTGCCGCGTCTGCGTCGTCACGGCCCTCGCCAGCGCGCTCTTCACCAA
TraesCS1A02G207300      GCGCGACCTGCTGTGCCGCATCTGCGTCGTCACGGCCCTCGCCAGCGCGCTCTTCACCAA
P-juncea-Lsi3           GCGCGACCTCCTGTGCCGCGTCTGCGTCGTCACGGCCCTCGCCAGCGCGCTCTTCACCAA
LOC-Hv123411373         GCGCGACCTCCTGTGCCGCGTCTGCGTCGTCACGGCACTCGCCAGCGCGCTCTTCACCAA
                        ********* ** ****** **************** ***********************

TraesCS1B02G221100      CGACACCTGCTGCGTCGTGCTCACGGAGTTCGTGCTCGAGCTCGCCGCCGAGCGCAACCT
TraesCS1D02G210500      CGACACCTGCTGCGTGGTGCTCACGGAGTTCGTGCTCGAGCTCGCCGCCGAGCGCAACCT
TraesCS1A02G207300      CGACACCTGCTGCGTCGTGCTCACGGAGTTCGTGCTCGAACTCGCCGCCGAGCGCAACCT
P-juncea-Lsi3           CGACACCTGCTGCGTCGTGCTCACGGAGTTCGTGCTCGAGCTCGCAGACGAGCGCAACCT
LOC-Hv123411373         CGACACCTGCTGCGTCGTGCTCACGGAGTTCGTGCTCGAGCTCGCCGCCGAGCGCAACCT
                        ************** ************************ ***** * ************
```

```
TraesCS1B02G221100      CCCCGCGAAGCCGTTCCTGCTGGCGCTGGCCACCAGCGCCAATATCGGGTCCAGCGCCAC
TraesCS1D02G210500      CCCCGCGAAGCCGTTCCTGCTGGCGCTGGCCACCAGCGCCAATATCGGGTCCAGCGCCAC
TraesCS1A02G207300      CCCCGCGAAGCCGTTCCTGCTGGCGCTGGCCACCAGCGCCAACATCGGGTCCAGCGCCAC
P-juncea-Lsi3           ACCCGCGAAGCCGTTCCTGCTGGCGCTGGCCACCAGCGCCAACATCGGGTCCAGCGCCAC
LOC-Hv123411373         CCCCGCGAAGCCGTTCCTGCTGGCGCTGGCCACCAGCGCCAACATCGGGTCCAGCGCCAC
                         ***************************************** *****************

TraesCS1B02G221100      GCCCATCGGGAACCCGCAGAACCTGGTCATCGCCTTCAACAGCAAGATCTCTTTCGTGGG
TraesCS1D02G210500      GCCCATCGGGAACCCGCAGAACCTGGTCATCGCCTTCAACAGCAAGATCTCCTTCGTGGG
TraesCS1A02G207300      GCCCATCGGGAACCCGCAGAACCTGGTCATCGCCTTCAACAGTAAGATCTCATTCGTGGG
P-juncea-Lsi3           GCCCATCGGGAACCCGCAGAACCTGGTTATCGCCTTCAACACCAAGATCTCCTTCGTGGG
LOC-Hv123411373         GCCCATCGGGAACCCGCAGAACCTGGTCATCGCCTTCAACAGCAAGATAACCTTCGTGGG
                        *************************** *************  *****  * ********

TraesCS1B02G221100      CTTCTTCCTCGGCATCCTGCCGGCCATGCTCGCAGGTATGGCCGTCAACATGGTCATGCT
TraesCS1D02G210500      CTTCTTCCTCGGCATCCTACCGGCCATGCTCGCAGGCATGGCCGTCAACATGGTCATGCT
TraesCS1A02G207300      CTTCCTCCTCGGCATCCTGCCGGCCATGCTCGTTGGTATGGCCGTCAACATGGTCATGCT
P-juncea-Lsi3           CTTCTTCCTCGGCATCCTGCCGGCCATGCTCGCCGGCATGGCCGTCAACATGGCCATGCT
LOC-Hv123411373         ATTCTTCCTCGGCATCCTGCCGGCCATGGTCGCAGGCATGGCCGTCAACATGGTCATGCT
                         *** ************* ********* ***  ** **************** ******

TraesCS1B02G221100      GCTCTGCATGTACTGGAAGGACCTCGAGGGGAGCAGTCCCGACGAGGTGGCCGCCGAGAA
TraesCS1D02G210500      GCTCTGCATGTACTGGAAGGACCTCGAGGGGAGCAGTCCCGACGCGGTGGCCGCCGAGAA
TraesCS1A02G207300      GCTCTGCATGTACTGGAAGGACCTCGAGGGGAGCAGTCCCGACGCGGTGGCCGCCGAGAA
P-juncea-Lsi3           GCTCTGCATGTACTGGAAGGACCTCGAGGGGAACAGTCCCGACGCGGTGGCCGCCGAGAA
LOC-Hv123411373         GCTCTGCATGTACTGGAAGGACCTCGAGGGGAACAGTCCCGACGCGATAGCCGCCGAGAA
                        ******************************** *********** * * ***********

TraesCS1B02G221100      GGAGATGGCGGCCGTCGAGGAGGGTCGCCTGCCGTCCCCGGCAACGTCGACGACGCTCAA
TraesCS1D02G210500      GGAGATGGCGGCAGTCGAGGAGGGTCGCCGGCCGTCCCCGGCAACGTCGACGACGCTCAA
TraesCS1A02G207300      GGAGATGGCGGCAGTCGAGGAGGGTCGCCGGCCGTCCCCGGCGACGTCGACGACGCTCAA
P-juncea-Lsi3           GGAGATGGCGGCCGTCGAGGAGGGTCGCCGGCCGTCCCCGGCAACGTCGACGACGCTCAA
LOC-Hv123411373         GGAGATGGCGGCAGTTGAGGAGGGTCGCCGGCCGTCCCCGGCGACGTCGGGGACGCTCAA
                        ************ ** ************* ************ ******  *********

TraesCS1B02G221100      GGGCCCCGGCCAGACGATGCTGGTCCAGTCGTCATCGCTGGTCGCCGCCGCCGGCCACGA
TraesCS1D02G210500      GAGCCCCGGCCAGACGATGCTGGTCCAGTCGCCGTCGCTGGTCGCGGCCGCCGACCACGA
TraesCS1A02G207300      GAGCCCCGGCCGGACGATGCTGGTCCAGTCCTCGTCGCTGGTCGCCGGCGCCGACCACGA
P-juncea-Lsi3           GAGCCCCGCCCAGACGATGTTGGTCCAGTCGTCGTCGCTGGTCGCGGCCGCCGACCTCGA
LOC-Hv123411373         GAGCCCCGGCCAGACGATGCTGGTTCAGTCGTCATCGCTGGTCGCGGCCGCCGACCACGA
                        * ****** ** ******* **** *****  * *********** * ***** ** ***

TraesCS1B02G221100      CTCGGTGATGATGGAGAGCATCTCGACCAAGCACCGGTGGTTCATACAGTGCTCGGCGCC
TraesCS1D02G210500      CTCGGTGATGGTGGAGAGCATCTCGACCAAGCACCGGTGGTTCATACAGTGCTCGGCGCC
TraesCS1A02G207300      GTCGGTGATGGTGGAGAGCATCTCGACCAAGCACCGGTGGTTCATACAGTGCTCGGCGCC
P-juncea-Lsi3           CTCGGCGATGGCGGAGAGCATCTCGACCAAGCACCGGTGGTTCATACAGTGCTCGGCGCC
LOC-Hv123411373         CTCGGTGATGGTGGAGAGCATCTCGACCAAGCACCGGTGGTTCATACAGTGCTCGGCGCC
                         **** ****  ************************************************

TraesCS1B02G221100      GCAGCGGAGGCTGTTCCTCAAGAGCTTCGCGTACATCGTGACCGCCGGCATGCTGGTCGC
TraesCS1D02G210500      GCAGCGGAGGCTGTTCCTCAAGAGCTTCGCGTACATCGTGACCGCCGGCATGCTGGTCGC
TraesCS1A02G207300      GCAGCGGAGGCTGTTCCTCAAGAGCTTCGCGTACGTCGTGACCGCCGGCATGCTGGTCGC
P-juncea-Lsi3           GCAGCGGAGGCTGTTCCTCAAGAGCTTCGCGTACATCGTGACCGCCGGCATGCTGGTCGC
LOC-Hv123411373         GCAGCGGAGGCTGTTCCTCAAGAGCTTCGCGTACATCGTGACCGCCGGCATGCTGGTCGC
                        ********************************** *************************

TraesCS1B02G221100      CTACATGCTCGGGCTCAACATGTCGTGGACGGCCATCACCACCGCCGTCGCGCTCATCGT
TraesCS1D02G210500      CTACATGCTCGGGCTCAACATGTCGTGGACCGCCATCACCACCGCCGTCGCGCTCATCGT
TraesCS1A02G207300      CTACATGCTGGGGCTCAACATGTCGTGGACGGCCATCACCACCGCCGTCGCCCTCATCGT
P-juncea-Lsi3           CTACATGCTGGGGCTCAACATGTCGTGGACCGCCATCACCACCGCCGTCGCCCTCATCGT
LOC-Hv123411373         CTACATGCTGGGGCTCAACATGTCGTGGACCGCCATCACCACCGCCGTCGCCCTCATCGT
                        ********* ******************** ******************** ********
```

```
TraesCS1B02G221100      GGTGGACTTCCGCGACGCGGAGCTCTGCCTCGGCAAGGTGTCTTACTCGCTGCTGGTTTT
TraesCS1D02G210500      GGTGGACTTCCGCGACGCCGAGCTCTGCCTCGGCAAGGTGTCTTACTCGCTGCTGGTTTT
TraesCS1A02G207300      GGTGGACTTCCGCGACGCCGAGCTCTGCCTCGGCAAGGTGTCTTACTCGTTGCTGGTTTT
P-juncea-Lsi3           CGTGGACTTCCGCGACGCCGAGCTCTGCCTCGGCAAGGTGTCTTACTCGCTGCTGGTTTT
LOC-Hv123411373         CGTGGACTTCCGCGACGCCGAGCTCTGCCTCGGCAAGGTGTCTTACTCGCTGCTGGTTTT
                         ***************** ****************************** **********

TraesCS1B02G221100      CTTCACGGGGATGTTCGTGACGGTGAGCGGGTTCAATAAGACGGGGCTCCCTGGTGCCAT
TraesCS1D02G210500      CTTCACGGGGATGTTCGTGACGGTGAGCGGGTTCAATAAGACGGGGCTCCCTGGCGCCAT
TraesCS1A02G207300      CTTCACGGGAATGTTCGTGACCGTGAGCGGGTTCAACAAGACGGGGCTCCCCGGCGCCAT
P-juncea-Lsi3           CTTCACGGGGATGTTCGTGACGGTGAGCGGGTTCAATAAGACGGGGCTCCCTGGCGCCAT
LOC-Hv123411373         CTTCACGGGGATGTTCGTGACGGTGAGCGGGTTCAATAAGACGGGGCTCCCTGGCGCCAT
                        ********* *********** ************** ************** ** *****

TraesCS1B02G221100      CTGGAACGTCATGGAGCCATACTCCAAGATCAACCACGTCAGGGGCATCACCGTGCTCTC
TraesCS1D02G210500      CTGGAAAGTCATGGAGCCATACTCCAAGATCAACCACGTCAGGGGCATCACCGTGCTCTC
TraesCS1A02G207300      CTGGAAAGTCATGGAGCCATACTCCAAGATCAACCACGTGAGGGGCATCACAGTGCTCTC
P-juncea-Lsi3           CTGGAACGTCATGGCGCCATACTCCAAGATCAACCACGTCAGCGGCGTCACCGTGCTCTC
LOC-Hv123411373         CTGGAACGTCATGGCGCCATACTCCAAGATCAGCCACGTCAGCGGCGTCACCGTGCTCTC
                        ****** ******* ***************** ****** ** *** **** ********

TraesCS1B02G221100      CCTCATCGTCATCATCCTCTCCAACCTAGCCTCCAACGTACCAACCGTGCTCCTGATGGG
TraesCS1D02G210500      CCTCATCGTCATCATCCTCTCCAACCTAGCCTCCAACGTACCAACCGTGCTGCTGATGGG
TraesCS1A02G207300      CCTCATCGTCATCGTCCTCTCCAACCTTGCCTCCAACGTACCAACCGTGCTGCTGATGGG
P-juncea-Lsi3           CCTCATCATCCTCCTCCTCTCCAACCTTGCCTCCAACGTACCAACCGTGCT---GATGGG
LOC-Hv123411373         CATCATCATCATTATCCTCTCCAACCTAGCCTCCAACGTACCGACCGTGCTGCTGATGGG
                        * ***** ** *  ************* ************** ********   ******

TraesCS1B02G221100      TGACGAGGTGGCGGCGTCGGCAGCGACGATCTCGGCGGCGGTGACGCGGTCGTGGCT
TraesCS1D02G210500      TGACGAGGTGGCGGCGTCGGCGGCGACGATCTCGGCGGCGGTGACGCGGTCGTGGCT
TraesCS1A02G207300      TGACGAGGTGGCGGCGTCGGCAGCGACGATCTCGACGGCGGTGACGCGGTCGTGGCT
P-juncea-Lsi3           TGACGAGGTGGCGGCGTCGGCAGCGACCATCTCGGCGGCGGTGACGCGGTCGTGGCT
LOC-Hv123411373         TGACGAGGTGGCGGCATCGGCAGCGACGATCTCGGCGGCAGTGACGCGGTCGTGGCT
                        *************** ***** ***** ****** ******* *****************

TraesCS1B02G221100      GCTGCTGGCGTGGGTGAGCACGGTGGCGGGTAACCTGTCCCTGCTGGGGTCGGCGGCGAA
TraesCS1D02G210500      GCTGCTGGCGTGGGTGAGCACGGTGGCGGGTAACCTGTCCCTGCTGGGGTCGGCGGCGAA
TraesCS1A02G207300      GCTGCTGGCGTGGGTGAGCACGGTGGCGGGTAACCTGTCCCTGCTGGGGTCGGCGGCGAA
P-juncea-Lsi3           GCTGCTGGCGTGGGTGAGCACGGTGGCGGGTAACCTGTCCCTGCTGGGGTCGGCGGCGAA
LOC-Hv123411373         GCTGCTGGCGTGGGTCAGCACGGTGGCGGGTAACCTGTCCCTGCTGGGGTCAGCGGCGAA
                        *************** *********************************** ********

TraesCS1B02G221100      CCTCATCGTGTGCGAGCAGGCACGCCGGGCGCCGCGCAACGCACACGACCTCACCTTCTG
TraesCS1D02G210500      CCTCATCGTGTGCGAGCAGGCACGCCGGGCGCCGCGCAACGCACACGACCTCACCTTCTG
TraesCS1A02G207300      CCTCATCGTGTGCGAGCAGGCACGCCGGGCGCCGCGCAACGCGCACGACCTCACCTTCTG
P-juncea-Lsi3           CCTCATCGTGTGCGAGCAGGCGCGCCGGGCGCCGCGCAACGCGCATGACCTCACCTTCTG
LOC-Hv123411373         CCTCATCGTGTGCGAGCAGGCACGCCGGGCGCCGCGCAACGCACACGACCTCACGTTCTG
                        ********************* ******************** ** ******** *****

TraesCS1B02G221100      GAGCCACGTCGTCTTCGGCGCGCCGTCCACGCTCGTTGTCACCGCCGTCGGCATACCACT
TraesCS1D02G210500      GAGCCACGTCGTCTTCGGCGCGCCGTCCACGCTCGTCGTCACCGCCGTCGGCATACCCCT
TraesCS1A02G207300      GAGCCACGTCGTCTTCGGCGCGCCGTCCACGCTCGTCGTCACCGCCGTCGGCATACCCCT
P-juncea-Lsi3           GAGCCACGTCGTCTTCGGCGCACCGTCCACGCTCGTCGTCACCGCCGTCGGCATACCCCT
LOC-Hv123411373         GAGCCACGTCGTCTTCGGCGCACCATCCACGCTCGTCGTCACCGCCGTCGGCATACCCCT
                        ********************* ** *********** ******************** **

TraesCS1B02G221100      CATCGGCATCATCAACGCTGCCTAGATCGA
TraesCS1D02G210500      CATCGGCATCATCAACGCTGCCTAGATCGA
TraesCS1A02G207300      CATTGGCATCATCAACGCTGCCTAGTTCGA
P-juncea-Lsi3           CATCGGCATCATCAACGCTGCCTGAATCCA
LOC-Hv123411373         CATCGGCATCATCAACGCTGCCTAGATTCA
                        *** *******************   *  *
```

Supplementary material S3

Details of primers used in this study for sequencing and amplicon size of the produced PCR products based on genomic DNA of bread wheat.

Primers are indicated by different colours as shown in Supplementary material S2.

Name	Sequence, 5'-3'	Length, bp	GC, %	T_m, ºC salt adj.	Amplicon size, bp
			PjLsi1		
PjLsi1-F1	GGAAATGGCCACCAACTCGAG	21	57	63	486
PjLsi1-R1	GAATCCAGGGGAAATGCCGGA	21	57	63	
PjLsi1-F2	TCCGGCATTTCCCCTGGATTC	21	57	63	1,631
PjLsi1-R2	GCGAGGGTGACGAACATCATG	21	57	63	
PjLsi1-F3	CATGATGTTCGTCACCCTCGC	21	57	63	877
PjLsi1-R3	TGGATCAGACCGGGATGTGGT	21	57	63	
			PjLsi2		
PjLsi2-F1	ATGGCGCTCGCGTCTCTCC	19	68	64	725
PjLsi2-R1	GTGCCGTTGAACGGGTGCG	19	68	64	
PjLsi2-F2	TTCCTCCTCGCGCTCGCCT	19	68	64	705
PjLsi2-R2	GCACGGAGATGCCGCCGG	18	78	65	
PjLsi2-F3	CCTACTCCAAGGTCGACAGC	20	60	63	986
PjLsi2-R3	GGCGGCTACATCTTTCCGATG	21	57	63	
			PjLsi3		
PjLsi3-F1	ATGGCGCTGGCGTCTCTGT	19	63	62	750
PjLsi3-R1	CGACGACTGGACCAACATCGT	21	57	63	
PjLsi3-F2	TTCCTGCTGGCGCTGGCCA	19	68	64	704
PjLsi3-R2	AGCACGGTGACGCCGCTGA	19	68	64	
PjLsi3-F3	GCCATACTCCAAGATCAACCAC	22	50	62	685
PjLsi3-R3	GATTCAGGCAGCGTTGATGATG	22	50	62	

Supplementary material S4

Details of primers used for RT-qPCR analysis of gene expression in *Psathyrostachys juncea*, with following indication of the position in the consolidated CDS sequences.

Name	Sequence, 5'-3'	Length, bp	GC, %	T_m, ºC salt adj.	Amplicon size, bp
PjLsi1-Fq	GCGGTGTCAGGTGGATCGAT	20	60	63	144
PjLsi1-Rq	GAAGCGGATGTAGGTGTAGGTC	22	55	64	
PjLsi2-Fq	GAACCTGATCGTGTGTGAGCAG	22	55	64	84
PjLsi2-Rq	ACGCCAAAGACGAGGTGGTTC	21	57	63	
PjLsi3-Fq	GGCGAACCTCATCGTGTGC	19	63	62	104
PjLsi3-Rq	CGACGAGCGTGGACGGTG	18	73	63	
PjActin1-Fq	TTCTTCATTGCCTGGTCCCC	20	55	60	83
PjActin1-Rq	TCTTGCTTGATGCGGTCCTT	20	50	58	

>PjLsi1

GGAAATGGCCACCAACTCGAGATCGAACTCCAGGGCGACCTTCTCCAGCGAGATCCACGA
CATCGCCACCGTGCAGAACTCCACCACGCCCAGCATGGTGTACTACACCGAGAGGTCCAT
CGCCGACTACTTCCCTCCTCATCTCCTCAAGAAGGTGGTGTCGGAGGTGGTGTCGACGTT
CCTGCTGGTGTTCGTGACGTGCGGGGCGGCGGCGATCAGCGCCCACGACGTCACGCGCAT
ATCGCAGCTCGGCCAGTCGGTCGCCGGCGGGCTCATCGTCGTCGTGATGATCTATGCCGT
CGGCCACATCTCCGGCGCGCACATGAACCCCGCCGTCACCCTCGCCTTCGCCATCTTCCG
GCATTTCCCCTGGATTCAGGTCCCGTTCTACTGGGCGGCGCAGTTCACGGGCGCGATCTG
CGCGTCGTTCGTTCTCAAGGCGGTGCTCCACCCCATCACCGTGATCGGCACCACCGAGCC
GGTGGGGCCGCACTGGCACGCGCTGGTCATCGAGATCGTCGTCACCTTCAACATGATGTT
CGTCACCCTCGCCGTCGCCACGGACACCAGAGCGGTGGGTGAGTTGGCTGGGTTGGCTGT
CGGTTCCTCCGTTTGCATTACCTCCATCTTCGCAGGG**GCGGTGTCAGGTGGATCGAT**GAA
CCCGGCGAGGACGCTGGGGCCGGCGCTGGCGAGCAACCGCTACTCCGGCCTCTGGCTCTA
CTTCCTGGGCCCCGTCCTCGGCACGCTCAGCGGGGCCTG**GACCTACACCTACATCCGCTT**
CGAGGACCCGCCCAAGGACGCGCCCCAGAAGCTCTCCTCCTTCAAGCTCCGCCGGCTGCA
GAGCCAGTCCGTCGCCGCCGACGACGACCTCGACCACATCCCGGTCTGATCCA

>PjLsi2

AGCCATGGCGCTCGCGTCTCTCCCCAAGGTGGTGCTGGGCTCCTTCGCCTTCGCCATCTT
CTGGATGATGGCGGTGTTCCCGTCGGTGCCCTTCCTGCCCATCGGCCGGACGGCGGGGTC
GCTGCTCTCCGCCGTGCTCATGATCGTCTTCCACGTGATCAGCCCCGACGACGCCTACGC
CTCCATCGACCTCCCCATCCTCGGCCTGCTCTTCTCCACCATGGTCGTCGGTGGCTACCT
CAAGAACGCCGGCATGTTCAAGCACCTGGGCACGCTCCTGGCCTGGAAGAGCCAGGGCGG

GCGCGACCTGCTCTGCCGCGTATGCGTCGTCACCGCGCTGGCCTCGGCGCTCTTCACCAA
CGACACCTGCTGCGTCGTGCTCACCGAGTTCGTGCTCGAGCTCGCCGCTGAGCGGAACCT
CCCGGCCAAGCCCTTCCTCCTCGCGCTCGCCTCCAGCGCCAACATCGGCTCCAGTGCCAC
CCCCATCGGCAACCCGCAGAATCTGGTCATCGCCTTCAACAGCAAGATCTCCTTCCCCAA
GTTTTTGATCGGCATCCTGCCAGCCATGCTCGCCGGCATGGCCGTCAACATGGTCATGCT
GCTCTGCATGTACTGGAAGGACCTTGAGGGCGTCGCCCCCGACGCGGCCGGCAAGCAGAT
GACGGTCGTGGAGGAGGGCCGCTCGCCATCCGTGGCATCGCACAAGAGCCCGCACCCGTT
CAACGGCACCGTGGCCGACGACGGGGCCGAGTCAATGATGGAGGAGAACATCTCGACCAA
GCACCCATGGTTCATGCAGTGCACGGAGCACCGGCGCAAGCTGTTCCTCAAGAGCTTCGC
CTACATCGTGACGGTGGGCATGGTGGTGGCCTACATGGCCGGGCTCAACATGTCGTGGAC
GGCCATCACCACCGCCATCGCGCTCGTCGTCGTCGACTTCCGGGACGCCGAGCCCTGCCT
CGTCAAGGTCTCCTACTCGCTGCTCGTCTTCTTCTCCGGCATGTTCATCACGGTGAGTGG
GTTCAACAAGACGGGGCTGCCGGGCGCCATCTGGAACTTCATGGCGCCCTACTCCAAGGT
CGACAGCGCCGGCGGCATCTCCGTGCTCTCCGTCATCATCCTCCTCCTCTCCAACCTCGC
ATCCAACGTACCCACAGTGCTGCTGATGGGGAACGAGGTGGCCACCGCGGCGGCGCTGAT
CTCGCCGGCAGCGGTGACGCGGTCGTGGCTGCTGCTAGCGTGGGTGAGCACGGTGGCGGG
CAACCTGTCGCTGCTGGGGTCGGCGGC**GAACCTGATCGTGTGTGAGCAG**GCGCGCCGGGC
GCCGCGGAACGCCTACGAGCTCACCTTCTG**GAACCACCTCGTCTTTGGCGT**GCCCTCCAC
CCTCATCGTCACCGCCGTCGGCATCCCCCTCATCGGAAAGATGTAGCCGCC

>PjLsi3

TAGCATGGCGCTGGCGTCTCTGTCCAAGGTGGTGATGGGGTCGGCGGCGTTCGGGGTGTT
CTGGGTGCTGGCCGTGTTCCCGTCGGTGCCTTTCCTGCCGATCGGCCGCACGGCGGGTGC
GCTGCTGGGGGCTGCGCTGATGGTCGTCTTCCACGTGATCAGCCCGGATGACGCCTACGC
CTCCGTGGACCTCCCCATCCTGGGCCTCCTCTTTGCCACCAAGGTCGTCGGCGGCTACCT
CAAGAGCGCCGGCATGTTCGGGCACCTGGGCCGGCTGCTGGCGTGGCGGAGCCAGGGCGG
GCGCGACCTCCTGTGCCGCGTCTGCGTCGTCACGGCCCTCGCCAGCGCGCTCTTCACCAA
CGACACCTGCTGCGTCGTGCTCACGGAGTTCGTGCTCGAGCTCGCAGACGAGCGCAACCT
ACCCGCGAAGCCGTTCCTGCTGGCGCTGGCCACCAGCGCCAACATCGGGTCCAGCGCCAC
GCCCATCGGGAACCCGCAGAACCTGGTTATCGCCTTCAACACCAAGATCTCCTTCGTGGG
CTTCTTCCTCGGCATCCTGCCGGCCATGCTCGCCGGCATGGCCGTCAACATGGCCATGCT
GCTCTGCATGTACTGGAAGGACCTCGAGGGGAACAGTCCCGACGCGGTGGCCGCCGAGAA
GGAGATGGCGGCCGTCGAGGAGGGTCGCCGGCCGTCCCCGGCAACGTCGACGACGCTCAA
GAGCCCCGCCCAGACGATGTTGGTCCAGTCGTCGTCGCTGGTCGCGGCCGCCGACCTCGA
CTCGGCGATGGCGGAGAGCATCTCGACCAAGCACCGGTGGTTCATACAGTGCTCGGCGCC
GCAGCGGAGGCTGTTCCTCAAGAGCTTCGCGTACATCGTGACCGCCGGCATGCTGGTCGC
CTACATGCTGGGGCTCAACATGTCGTGGACCGCCATCACCACCGCCGTCGCCCTCATCGT
CGTGGACTTCCGCGACGCCGAGCTCTGCCTCGGCAAGGTGTCTTACTCGCTGCTGGTTTT
CTTCACGGGGATGTTCGTGACGGTGAGCGGGTTCAATAAGACGGGGCTCCCTGGCGCCAT
CTGGAACGTCATGGCGCCATACTCCAAGATCAACCACGTCAGCGGCGTCACCGTGCTCTC
CCTCATCATCCTCCTCCTCTCCAACCTTGCCTCCAACGTACCAACCGTGCTGATGGGTGA
CGAGGTGGCGGCGTCGGCAGCGACCATCTCGGCGGCGGCGGTGACGCGGTCGTGGCTGCT
GCTGGCGTGGGTGAGCACGGTGGCGGGTAACCTGTCCCTGCTGGGGTCGGC**GGCGAACCT**
CATCGTGTGCGAGCAGGCGCGCCGGGCGCCGCGCAACGCGCATGACCTCACCTTCTGGAG
CCACGTCGTCTTCGGCG**CACCGTCCACGCTCGTCG**TCACCGCCGTCGGCATACCCCTCAT
CGGCATCATCAACGCTGCCTGAATCCA

Supplementary material S5

Details of allele-specific primers used for ASQ genotyping of three genes *PjLsi* in *Psathyrostachys juncea*.

PjLsi1

GTGGGTGAGTTGGCTGGGTTGGCTGTCGGTTCCTCCGTTTGCATTACCTCCATCTTCGCA
GGGTAAATATATACTASTACGTTAATTAGAGATTAATTAATTAACAACTCCCCTTGCCGG
CTAGTTAAGAAATCACTGCTTGGACTACGTTAGTATCTAAAATTTATTCTCACTCGCGTC
[S]=[C] or [G]

K-1698, PI-314521 (High Si):
PjLsi1-SNP1-F1: AATCTCTAATTAACGTACT 19bp, 26%GC, Tm=46C
(RevCom): AGTACGTTAATTAGAGATT

K-46752, p-0241 (Low Si):
PjLsi1-SNP1-F2: AATCTCTAATTAACGTAGT 19bp, 26%GC, Tm=46C
(RevCom): ACTACGTTAATTAGAGATT

PjLsi1-SNP1-R: ATCTTCGCAGGGTAAAT 17bp, 41%GC, Tm=47C
Amplicon size: 43 bp

ASQ primers:
PjLsi1-SNP1-F1: GTCCTTGCGAAGGCATCCAATCTCTAATTAACGTACT
PjLsi1-SNP1-F2: GTCCTTGCGAAGGCCAACAATCTCTAATTAACGTAGT
PjLsi1-SNP1-R: ATCTTCGCAGGGTAAAT

PjLsi2

AGCGCCGGCGGCATCTCCGTGCTCTCCGTCATCATCCTCCTCCTCTCCAACCTCGCATCC
AACGTACCCACAGGTATWCATATGCATGCATGCATGCATGGATTTTGTACCTCGGTACTA
TATATGACAGGAGCTAGTTAATTTGTCACATATGAACATATTATTCTGGTAAGAGTGACA

[W]=[A] or [T]

K-1698, PI-314521 (High Si):
PjLsi2-SNP4-F1: CAACGTACCCACAGGTATTC 20 bp, 50%GC, Tm=58CC

K-46752, p-0241 (Low Si):
PjLsi2-SNP4-F2: CAACGTACCCACAGGTATAC 20 bp, 50%GC, Tm=58CC

PjLsi2-SNP4-R: AGGTACAAAATCCATGCATGCA 22 bp, 41%GC, Tm=58C
(RevCom): TGCATGCATGGATTTTGTACCT
Amplicon size: 53 bp

ASQ primers:
PjLsi2-SNP4-F1: GTCCTTGCGAAGGCATCCCAACGTACCCACAGGTATTC
PjLsi2-SNP4-F2: GTCCTTGCGAAGGCCAACCAACGTACCCACAGGTATAC
PjLsi2-SNP4-R: AGGTACAAAATCCATGCATGCA

PjLsi3

AGCACGGTGGCGGGTAACCTGTCGCTGCTGGGGTCGGCGGCGAACCTCATCGTGTGCGAGCAGG
CACGCCGAGCGCCGCGCAACGCRCAYGACCTCACSTTCTGGAGCCACGTCGTCTTCGGCGCACC
GTCCACGCTCGTCGTCACCGCCGTCGGCATACCCCTCATCGGCATCATCAACGCTGCCTGAATC

[R]=[A] or [G]
[Y]=[C] or [T]
[S]=[C] or [G]

K-1698, PI-314521 (High Si):
PjLsi3-SNP25-27-F1: CGCGCATGACCTCACCT 17bp; 65%GC; Tm=57C

K-46752, p-0241 (Low Si):
PjLsi3-SNP25-27-F2: CGCACACGACCTCACGT 17bp; 65%GC; Tm=57C

PjLsi3-SNP25-27-R: TGCGCCGAAGACGACGT 17bp; 65%GC; Tm=57C
(RevCom): ACGTCGTCTTCGGCGCA
Amplicon size: 43 bp

ASQ primers:
PjLsi3-SNP25-27-F1: GTCCTTGCGAAGGCATCCCGCGCATGACCTCACCT
PjLsi3-SNP25-27-F2: GTCCTTGCGAAGGCCAACCGCACACGACCTCACGT
PjLsi3-SNP25-27-R: TGCGCCGAAGACGACGT

Supplementary material S6

Microscopy analysis in *Psathyrostachys juncea*.

Group A. High Si content in leaf biomass

(1) K-1698, PI-314521. Root surface

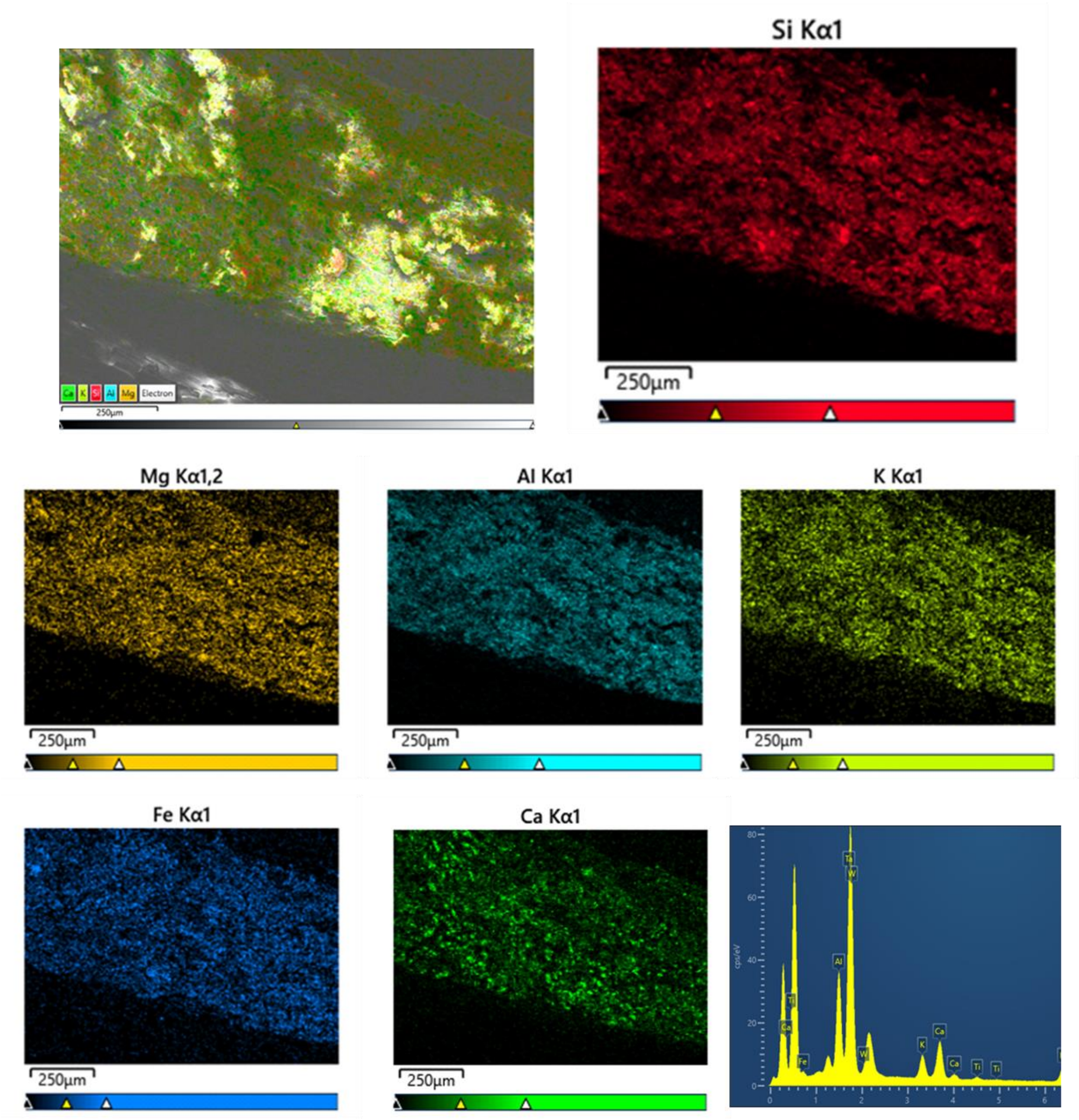

Group A. High Si content in leaf biomass

(1) K-1698, PI-314521. Root cross-section

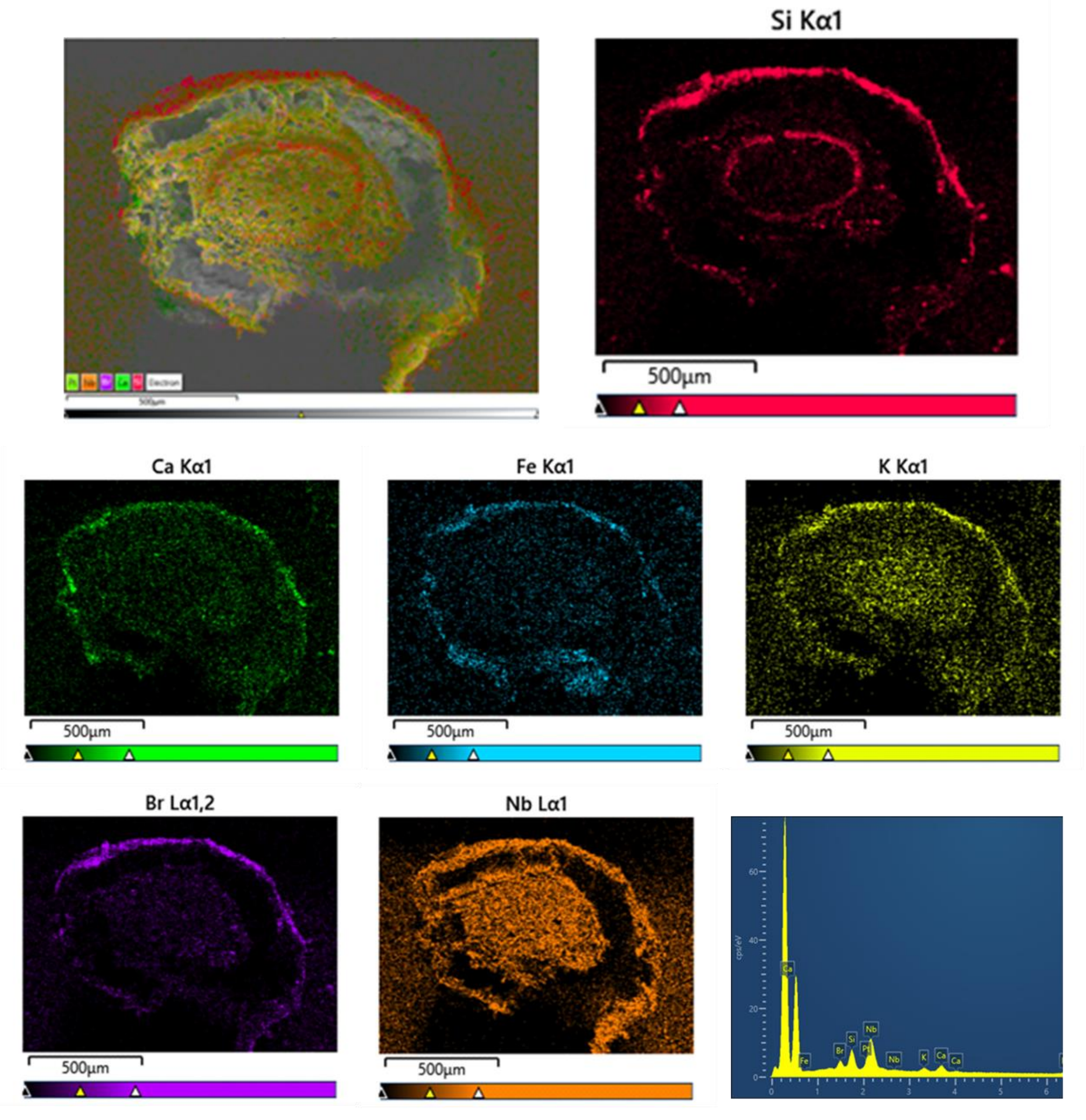

Group A. High Si content in leaf biomass

(1) K-1698, PI-314521. Leaf surface

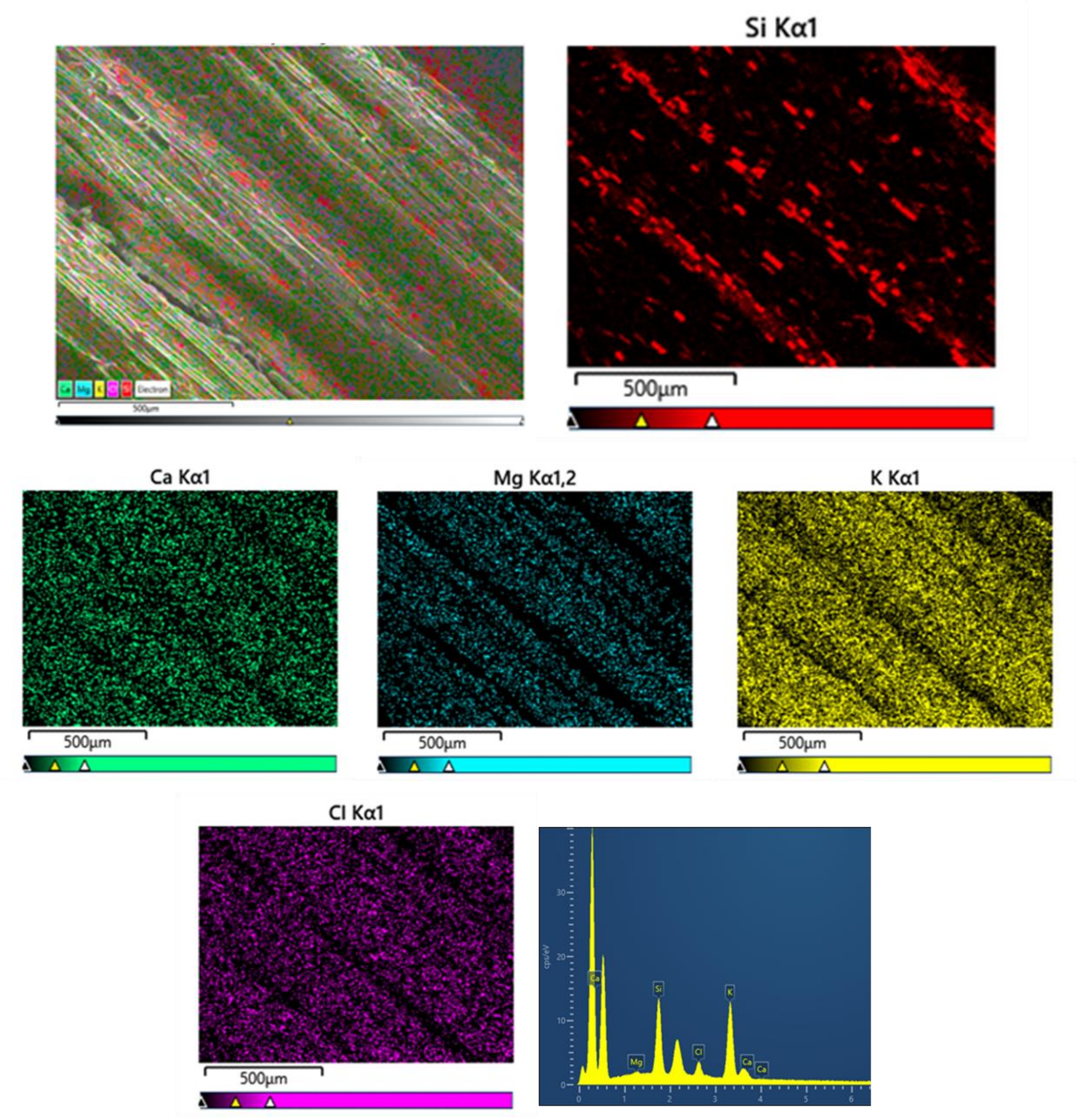

Group A. High Si content in leaf biomass

(2) K-1749, PI-565067. Root surface

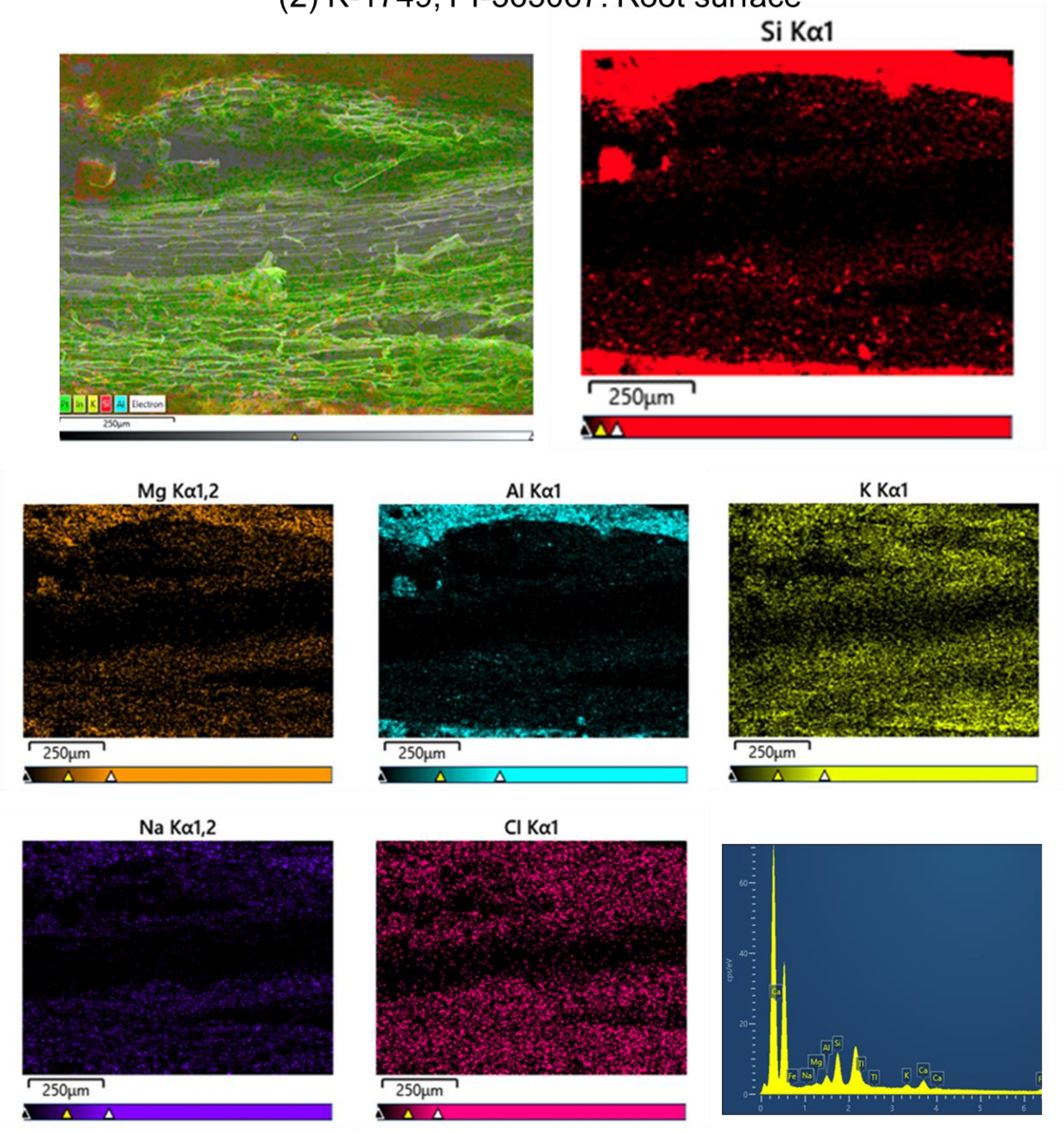

Group A. High Si content in leaf biomass
(2) K-1749, PI-565067. Root cross-section

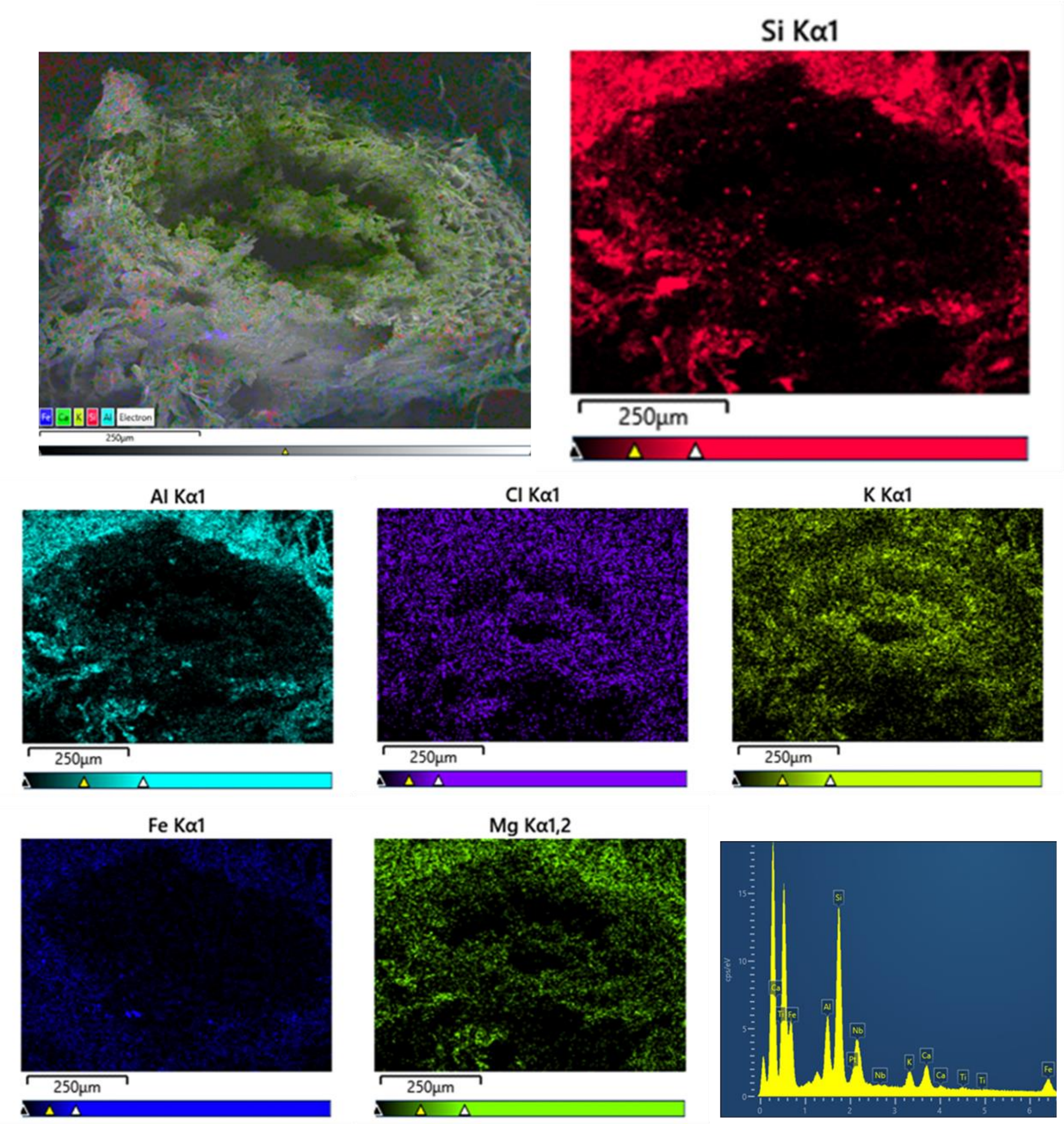

Group A. High Si content in leaf biomass

(2) K-1749, PI-565067. Leaf surface

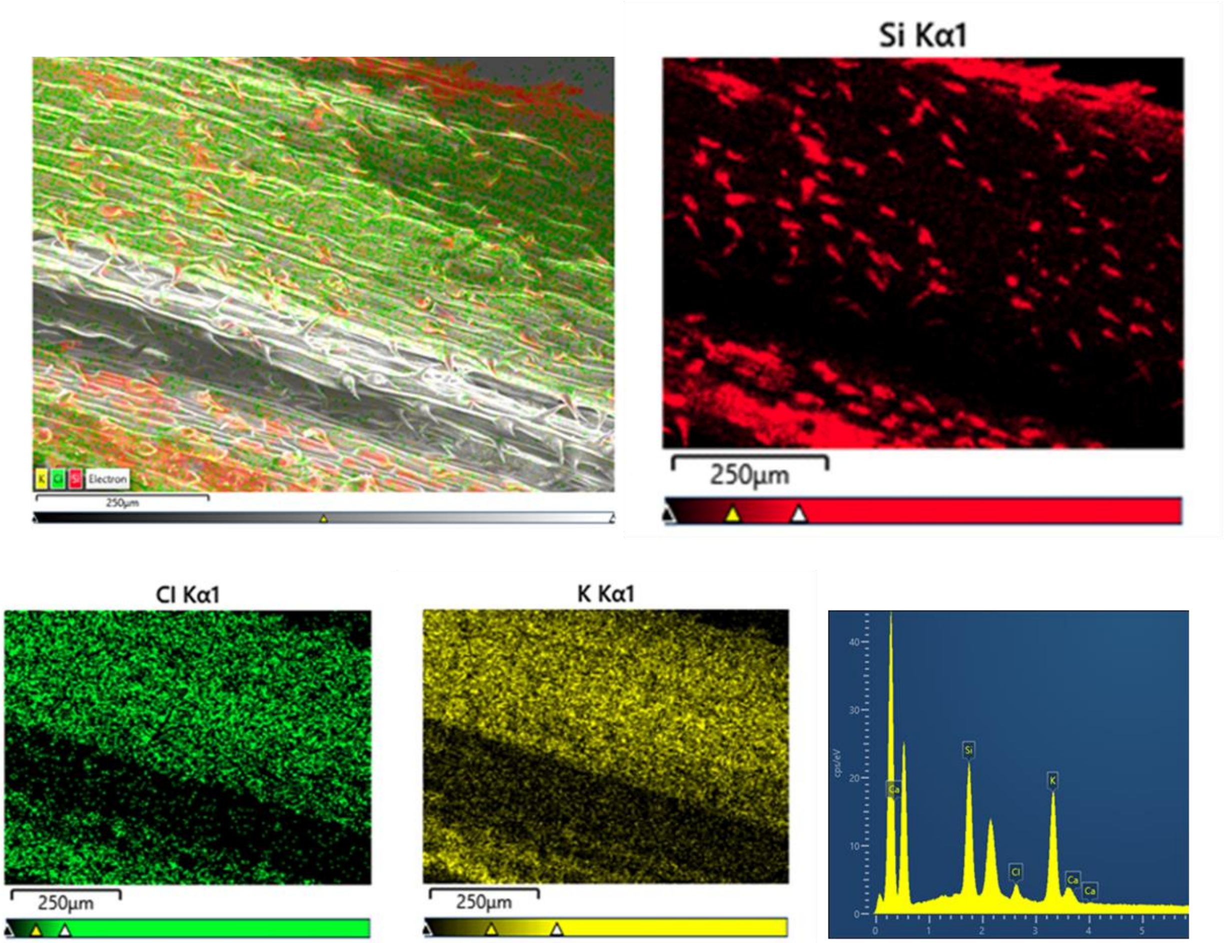

Group A. High Si content in leaf biomass

(3) K-1752, PI-565073. Root surface

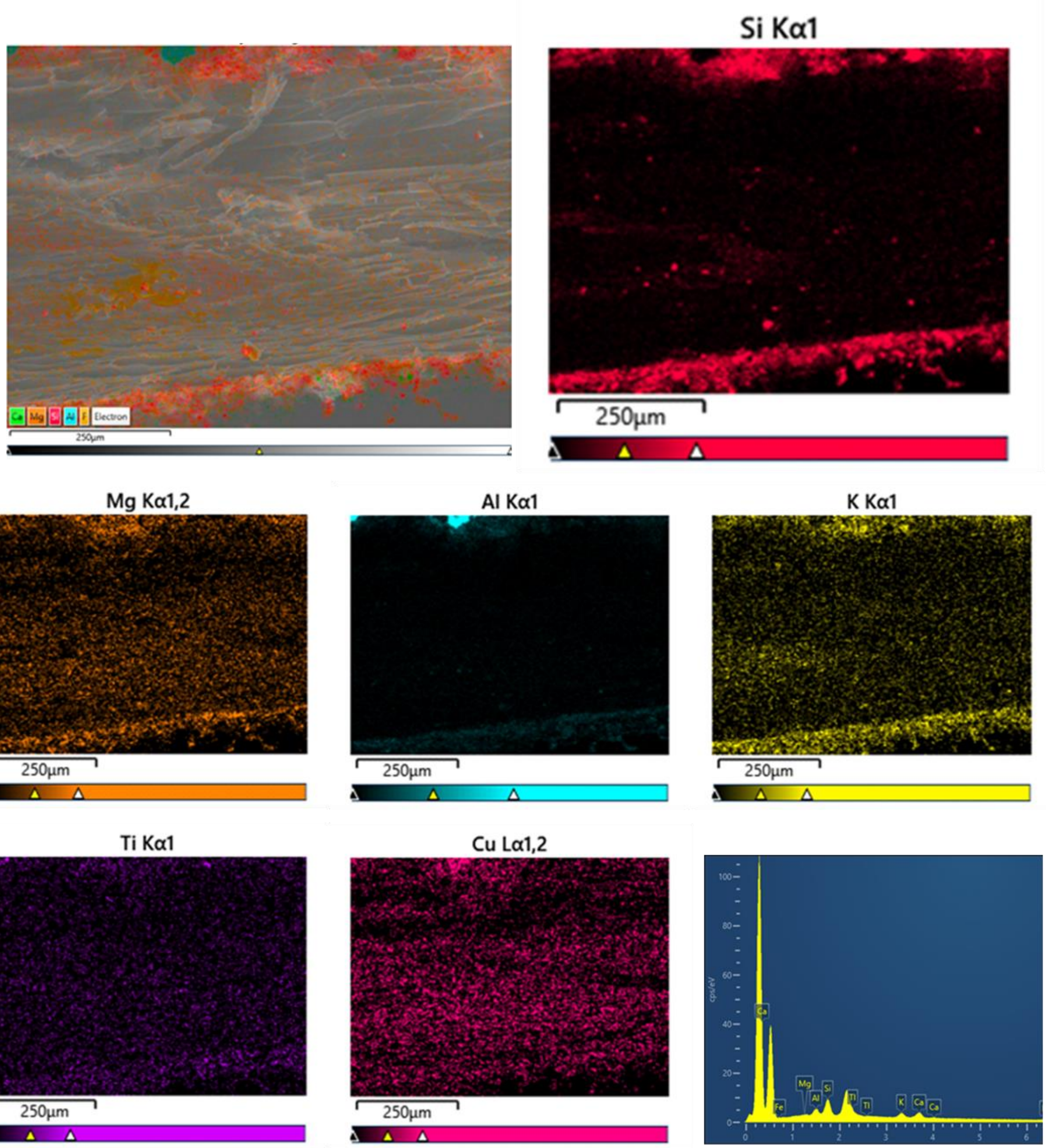

Group A. High Si content in leaf biomass

(3) K-1752, PI-565073. Root cross-section

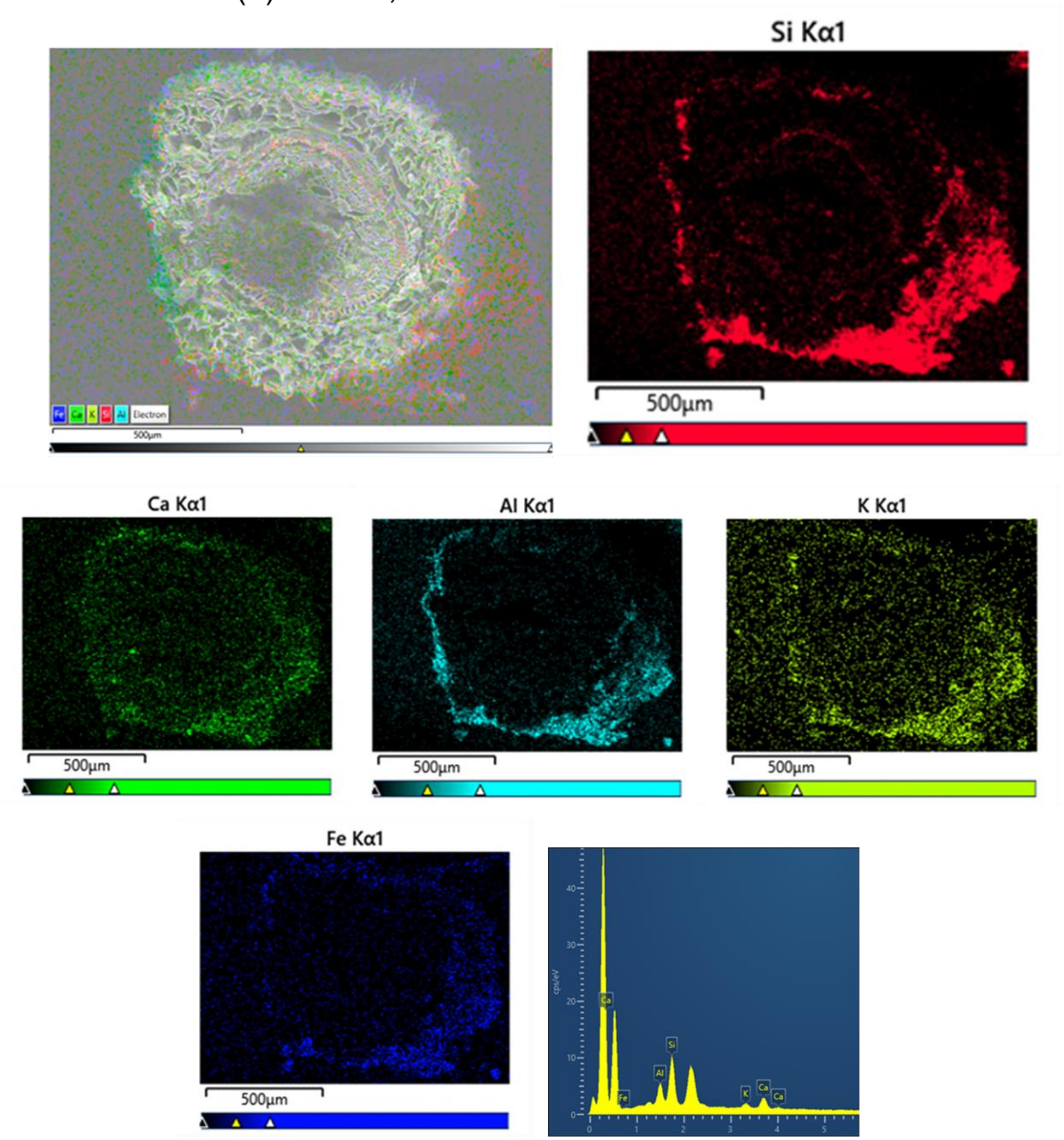

Group A. High Si content in leaf biomass

(3) K-1752, PI-565073. Leaf surface

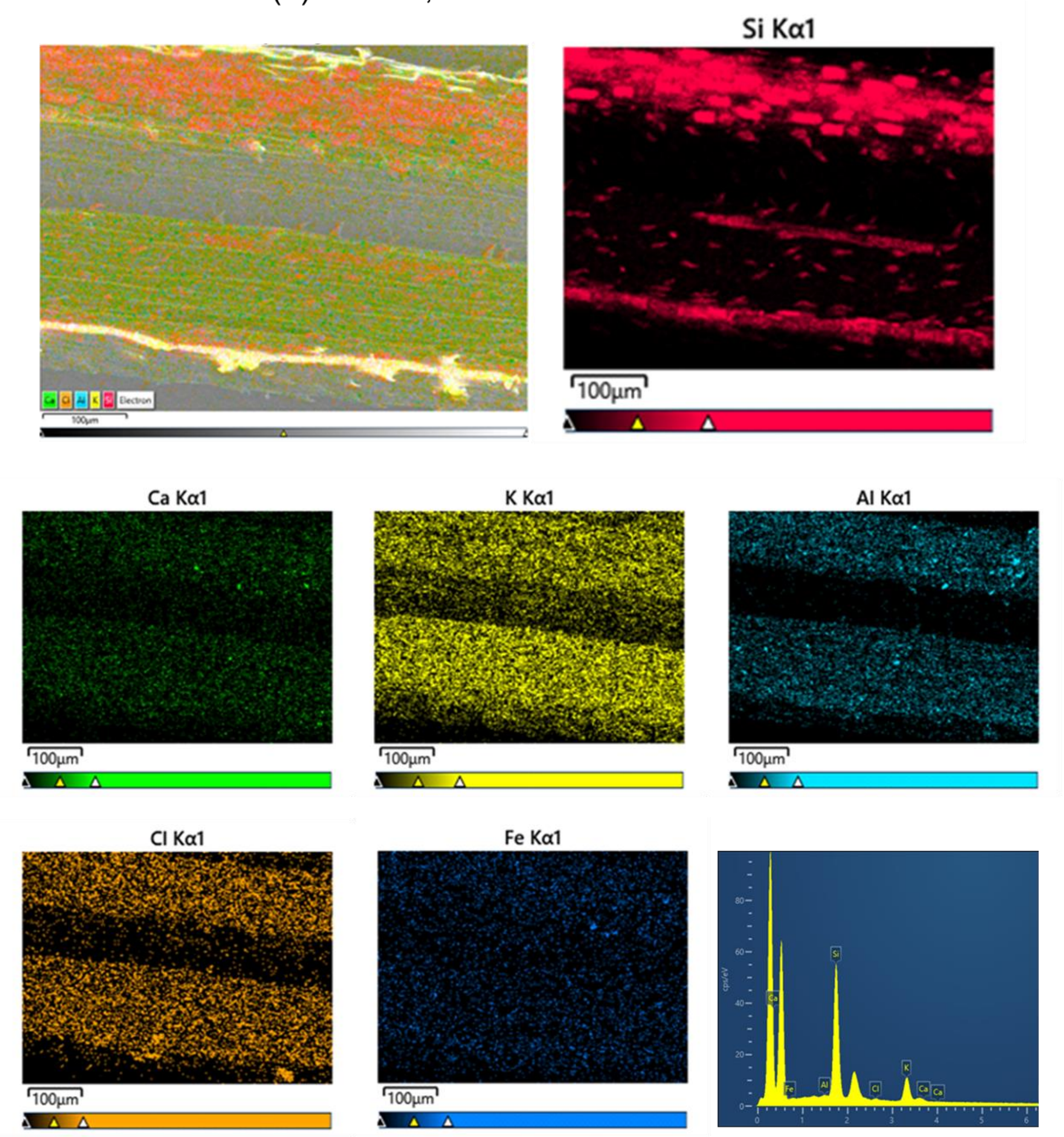

Group B. Low Si content in leaf biomass

(4) K-1716, PI-430875. Root surface

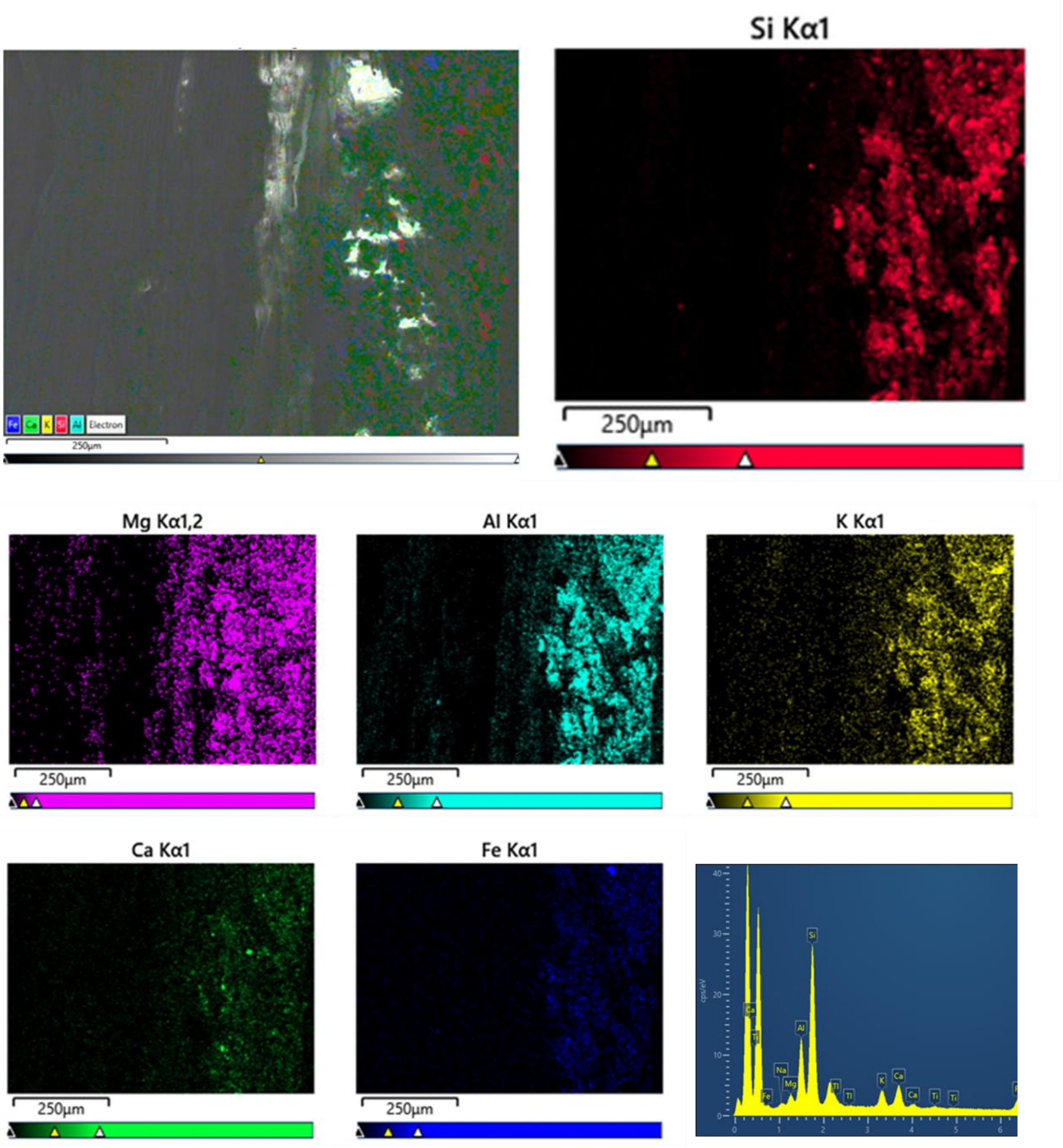

Group B. Low Si content in leaf biomass

(4) K-1716, PI-430875. Root cross-section

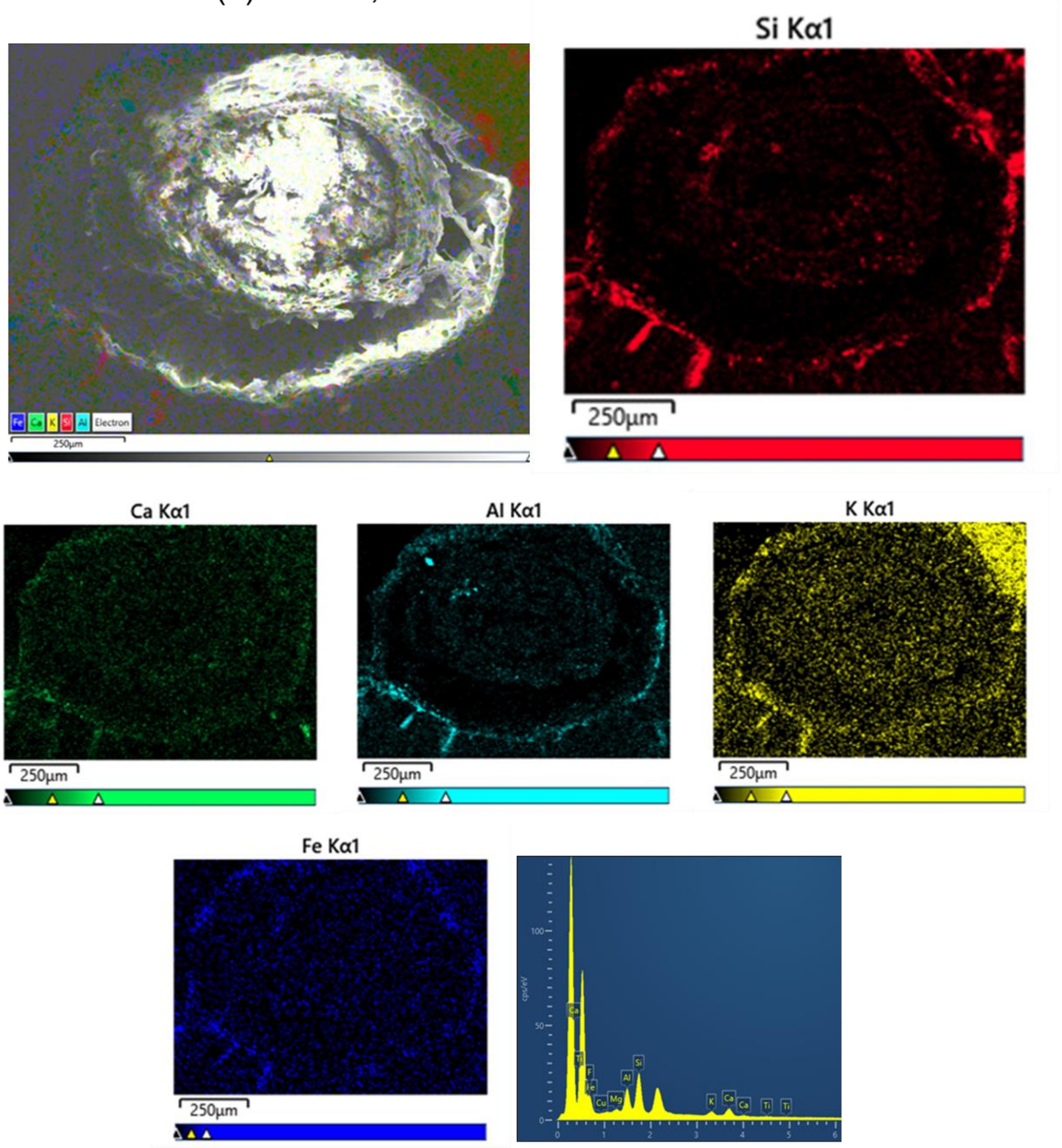

Group B. Low Si content in leaf biomass

(4) K-1716, PI-430875. Leaf surface

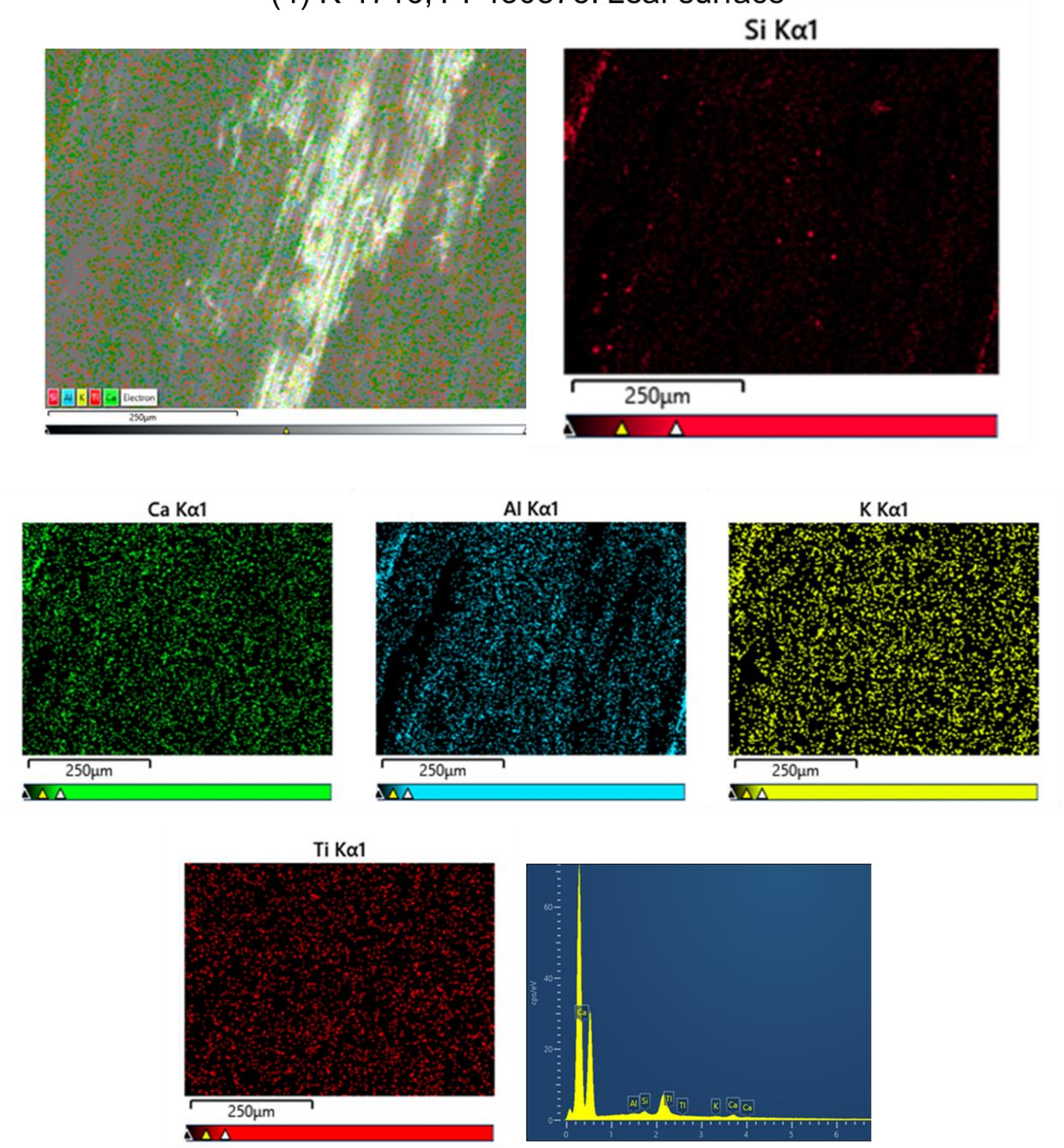

Group B. Low Si content in leaf biomass

(5) K-1731, PI-502577. Root surface

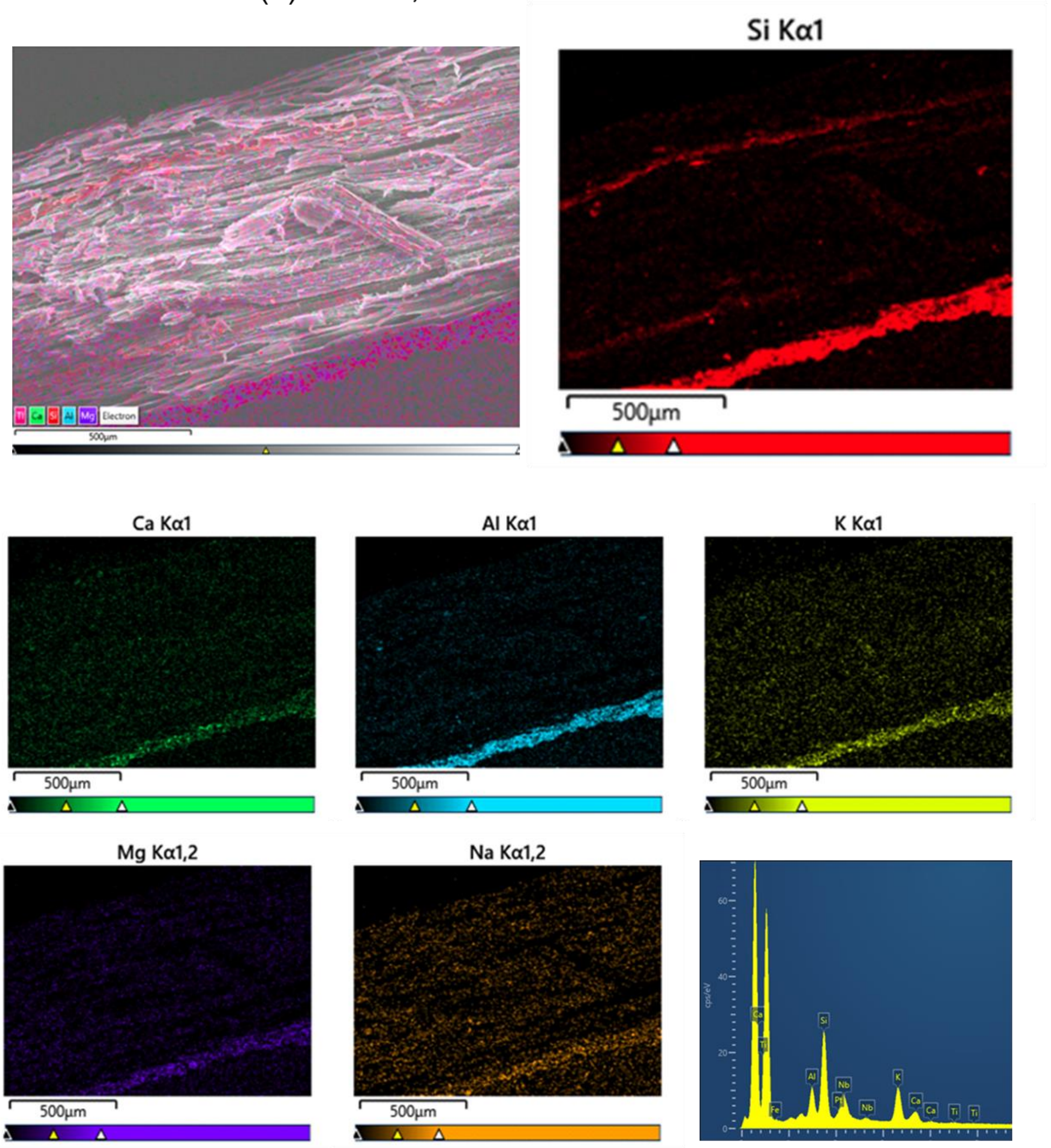

Group B. Low Si content in leaf biomass

(5) K-1731, PI-502577. Root cross-section

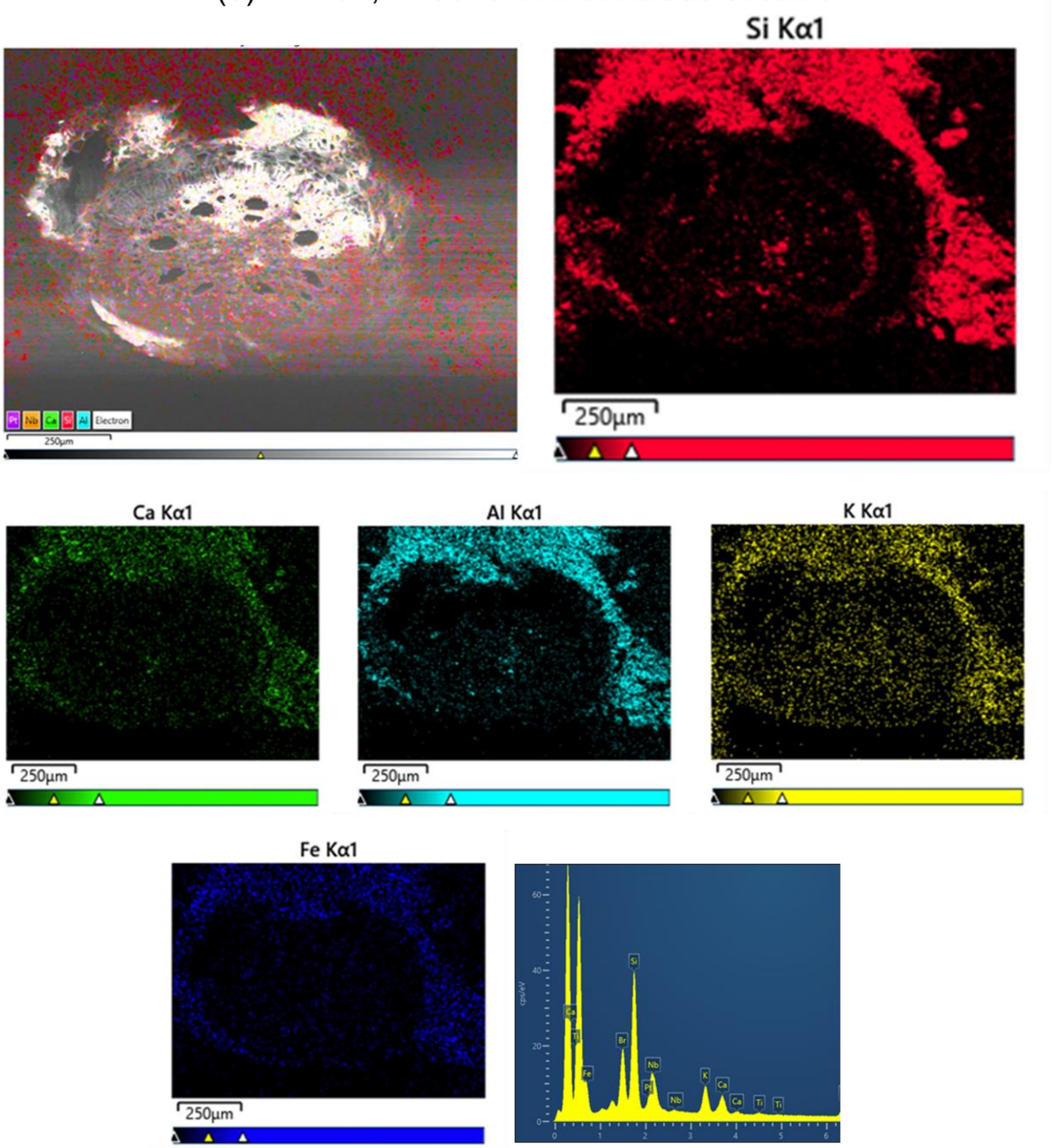

Group B. Low Si content in leaf biomass

(5) K-1731, PI-502577. Leaf surface

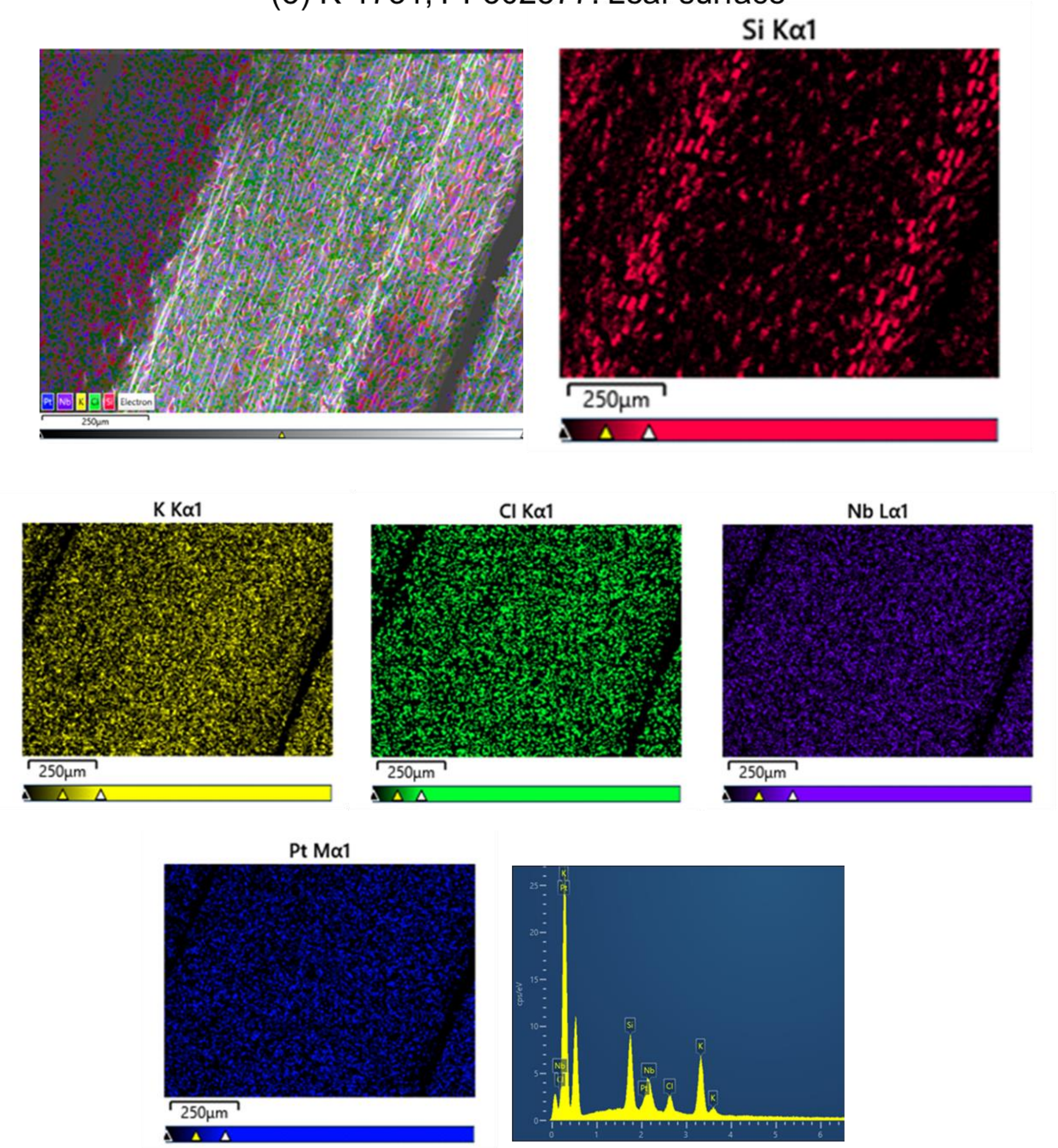

Group B. Low Si content in leaf biomass

(6) K-46752, p-0241. Root surface

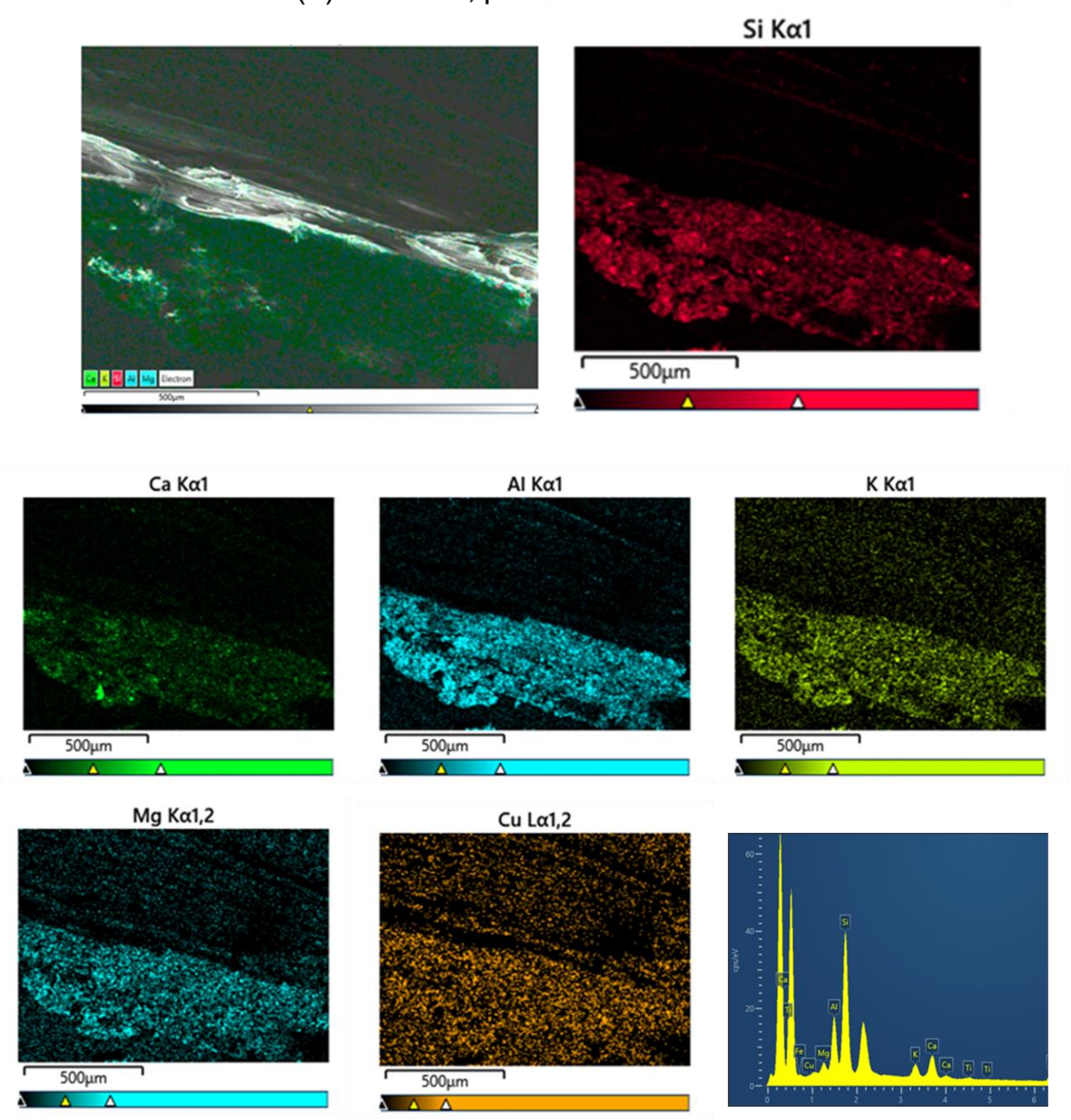

Group B. Low Si content in leaf biomass

(6) K-46752, p-0241. Root cross-section

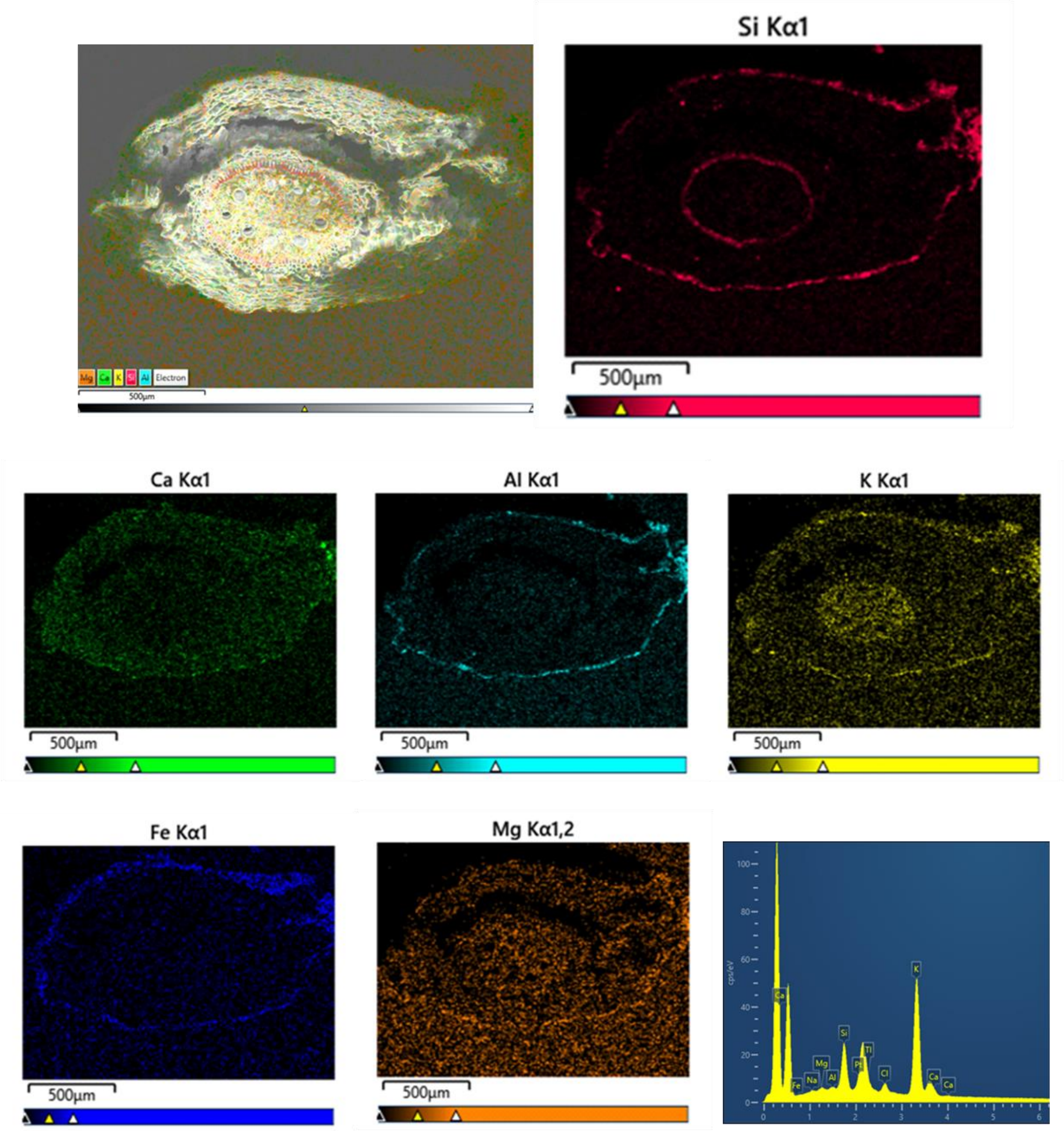

Group B. Low Si content in leaf biomass

(6) K-46752, p-0241. Leaf surface

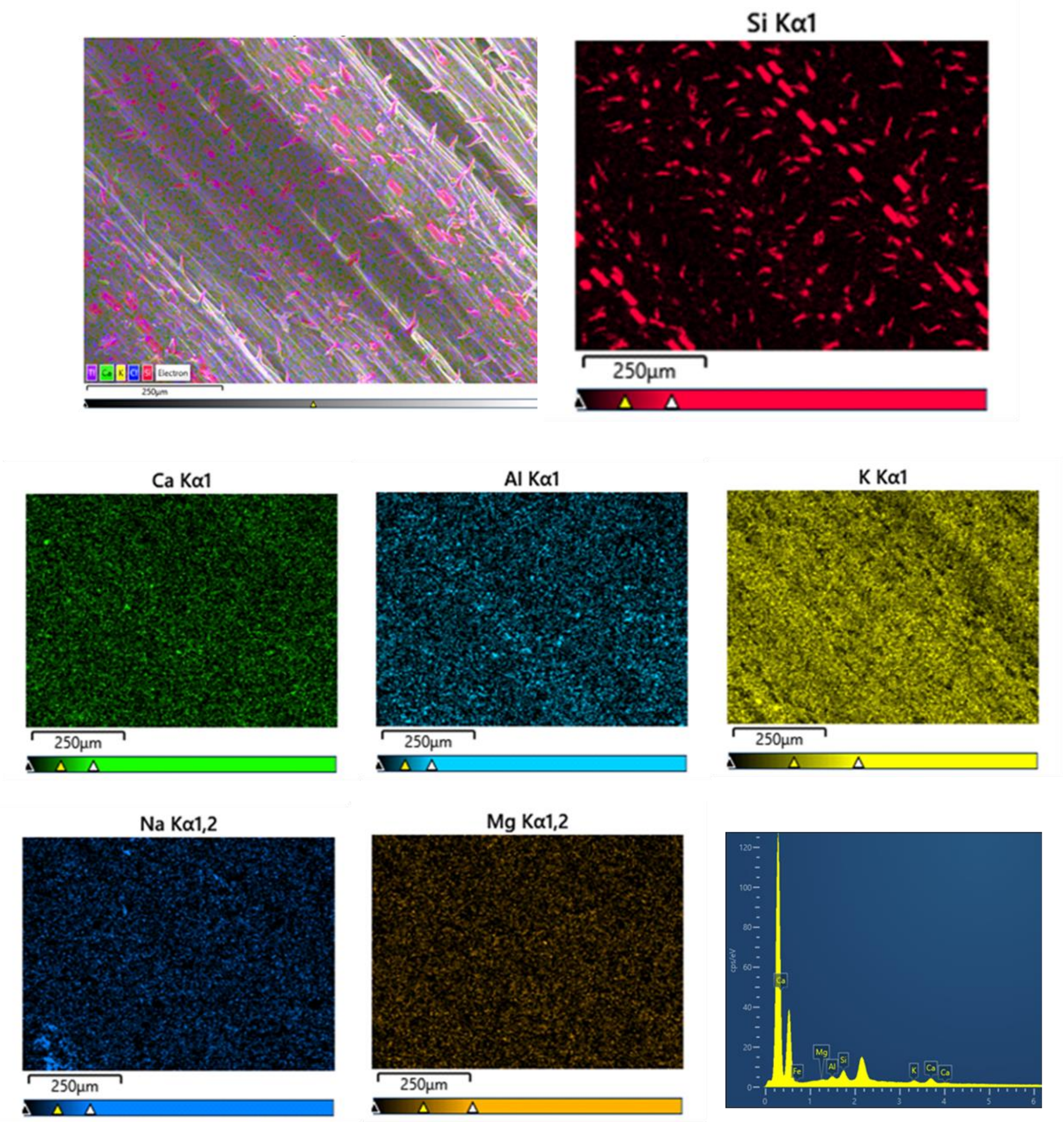

Supplementary material S7

Consolidated sequences of studied *Lsi1*, *Lsi2* and *Lsi3* genes in *Psathyrostachys juncea* produced in this study using Sanger sequencing. Primers for sequencing are indicated in the presented sequences. Exons are indicated by different colours while introns did not highlighted by colours.

PjLsi1

>PjLsi1-K1698-PI314521-highSi-genomic

GGAAATGGCCACCAACTCGAGATCGAACTCCAGGGCGACCTTCTCCAGCGAGATCCACGA
CATCGCCACCGTGCAGAACTCCACCACGCCCAGCATGGTGTACTACACCGAGAGGTCCAT
CGCCGACTACTTCCCTCCTCATCTCCTCAAGAAGGTGAGCCACATCGACCGATCCATCGA
TCATATAACTAGCACCGGCTGTGTGATCGATCACTCTTCGAGATGAATTAGGATCACTGC
ATAATTGACGCGAACTGTTCTCTCTATCTGCAGGTGGTGTCGGAGGTGGTGTCGACGTTC
CTGCTGGTGTTCGTGACGTGCGGGGCGGCGGCGATCAGCGCCGACGACGTCACGCGCATA
TCGCAGCTCGGCCAGTCGGTCGCCGGCGGGCTCATCGTCGTCGTGATGATCTATGCCGTC
GGCCACATCTCCGGCGCGCACATGAACCCCGCCGTCACCCTCGCCTTCGCCATCT**TCCGG**
CATTTCCCCTGGATTCAGGTACTACATACGTTACGCAACAGGAAACTCCTCTGCAGCAAT
CAAGCAGTGACGCTGCATGTGCACTATTTGCTAAGGCCTAAATCATTCAGCCACAAAAGC
CATCCCACTACTGATCGTAACGTCAATTCTCTCGCTTTCTCTTAATCTGTCATTTCCAAC
GAAGATTTTGGCAAACACCCCACTCATCGATCGAGCATCGATTGGATGACACAAGTGGCA
CAAGAGTACCCATGGATTGCTTTGTTATGGCTACAGATGCTCAGGACTGCCTCCGGTTAA
AGAAATGTTCAGCGCCTTATTAGATTAGTACAGTTGCTTCAGTATTTGAAAAACCGTTTT
CAAATTAGAAAATGGCATGATCACCATGGGCTGATCTAGTCATGGCTAAAAACTCAGTTT
ACTCACACGTACTTTTCAGGNNNNNNNNNNNNNNNNNNNNNNNNNNNNNNNNNNNNNNNN
AGGTCCCGTTCTACTGGGCGGCGCAGTTCACGGGCGCGATCTGCGCGTCGTTCGTTCTCA
AGGCGGTGCTCCACCCCATCACCGTGATCGGCACCACCGAGCCGGTGGGGCCGCACTGGC
ACGCGCTGGTCATCGAGATCGTCGTCACCTTCAA**CATGATGTTCGTCACCCTCGC**CGTCG
CCACGGACACCAGAGCGGTAAGCCGGTCGCCCGCCCGCCGTTGGATGATATTGCCATTTG
TCGATCTCTGTAAAACCATGGGTTCTGACGTTGTTGTTGTTACTGTGTTCATTCATGCAG
GTGGGTGAGTTGGCTGGGTTGGCTGTCGGTTCCTCCGTTTGCATTACCTCCATCTTCGCA
GGGTAAATATATACTAGTACGTTAATTAGAGATTAATTAATTAACAACTCCCCTTGCCGG
CTAGTTAAGAAATCACTGCTTGGACTACGTTAGTATCTAAAATTTATTCTCACTCGCGTC
GAAAACGAGGGCTTAGTTAACTAGGTCCAATTAATCACCACTTGGCTCTTGGCAACCAAG
GGAAGAGCCAAGCCGGAGTCACTGATAACATTCTTAATTTTGGTAACCTTGACCATGTCC
TCCATGGCCTAAGCTGGTATGGAACGATGGGACACGCTAAGTACACTGACATGATTGATA
GTGGCCGGCCGCGTCAGACGATAAATATGTTAGTCTAGTAGTATTATGCTCTGATATAAT
AAACGGGATTAGTGATGGAATAAAACTGAGACGCGACGGCGACCGATCGAGTGCAGATCG
ATGAACCCGGCGAGGACGCTGGGGCCGGCGCTGGCGAGCAACCGCTACTCCGGCCTCTGG
CTCTACTTCCTGGGCCCCGTCCTCGGCACGCTCAGCGGGGCCTGGACCTACACCTACATC
CGCTTCGAGGACCCGCCCAAGGACGCGCCCCAGAAGCTCTCCTCCTTCAAGCTCCGCCGG
CTGCAGAGCCAGTCCGTCGCCGCCGACGACGACCTCGACCACATCCCGGTCTGATCCA

>PjLsi1-K46752-p0241-lowSi-genomic

GGAAATGGCCACCAACTCGAGATCGAACTCCAGGGCGACCTTCTCCAGCGAGATCCACGA
CATCGCCACCGTGCAGAACTCCACCACGCCCAGCATGGTGTACTACACCGAGAGGTCCAT
CGCCGACTACTTCCCTCCTCATCTCCTCAAGAAGGTGAGCCACATCGACCGATCCATCGA
TCATATAACTAGCACCGGCTGTGTGATCGATCACTCTTCGAGATGAATTAGGATCACTGC
ATAATTGACGCGAACTGTTCTCTCTATCTGCAGGTGGTGTCGGAGGTGGTGTCGACGTTC
CTGCTGGTGTTCGTGACGTGCGGGGCGGCGGCGATCAGCGCCGACGACGTCACGCGCATA
TCGCAGCTCGGCCAGTCGGTCGCCGGCGGGCTCATCGTCGTCGTGATGATCTATGCCGTC
GGCCACATCTCCGGCGCGCACATGAACCCCGCCGTCACCCTCGCCTTCGCCATCT**TCCGG**
CATTTCCCCTGGATTCAGGTACTACATACGTTACGCAACAGGAAACTCCTCTGCAGCAAT
CAAGCAGTGACGCTGCATGTGCACTATTTGCTAAGGCCTAAATCATTCAGCCACAAAAGC
CATCCCACTACTGATCGTAACGTCAATTCTCTCGCTTTCTCTTAATCTGTCATTTCCAAC
GAAGATTTTGGCAAACACCCCACTCATCGATCGAGCATCGATTGGATGACACAAGTGGCA
CAAGAGTACCCATGGATTGCTTTGTTATGGCTACAGATGCTCAGGACTGCCTCCGGTTAA
AGAAATGTTCAGCGCCTTATTAGATTAGTACAGTTGCTTCAGTATTTGAAAAACCGTTTT
CAAATTAGAAAATGGCATGATCACCATGGGCTGATCTAGTCATGGCTAAAAACTCAGTTT
ACTCACACGTACTTTTCAGGNNNNNNNNNNNNNNNNNNNNNNNNNNNNNNNNNNNNNNNN
AGGTCCCGTTCTACTGGGCGGCGCAGTTCACGGGCGCGATCTGCGCGTCGTTCGTTCTCA
AGGCGGTGCTCCACCCCATCACCGTGATCGGCACCACCGAGCCGGTGGGGCCGCACTGGC
ACGCGCTGGTCATCGAGATCGTCGTCACCTTCAA**CATGATGTTCGTCACCCTCGC**CGTCG
CCACGGACACCAGAGCGGTAAGCCGGCCGCCCGCCCGCCGTTGGATGATATTGCCATTTG
TCGATCTCTGTAAACCATGGGTTCTGACGTTGTTGTTGTTACTGTGTTCATTCATGCAGG
TGGGTGAGTTGGCTGGGTTGGCTGTCGGTTCCTCCGTTTGCATTACCTCCATCTTCGCAG
GGTAAATTTATACTACTACGTTAATTAGAGATTAATTAATTAACAACTCCCCTTGCCGGC
TAGTTAAGAAATCACTACTTGGACTACGTTAGTATCTAAAATTTATTCTCACTCGCGTCG
AAAACGAGGGCTTAGTTAACTAGGTCCAATTAATCACCACTTGGCTCTTGGCAACCAAGG
GAAGAGCCAAGCCGGAGTCACTGATAACATTCTTAATTTTGGTAACCTTGACCATGTCCT
CCATGGCCTAAGCTGGTATGGAACGATGGGACACGCTAAGTACACTGACATGATTGATAG
TGGCCGGCCGCGTCAGACGATGAATATGTTAGTCTAGTAGTATTATGCTCTGATATAATA
AACGGGATTAGTGATGGAATAAAACTGAGACGCGACGGCGACCGATCGAGTGCAGATCGA
TGAACCCGGCGAGGACGCTGGGGCCGGCGCTGGCGAGCAACCGCTACTCCGGCCTCTGGC
TCTACTTCCTGGGCCCCGTCCTCGGCACGCTCAGCGGGGCCTGGACCTACACCTACATCC
GCTTCGAGGACCCGCCCAAGGACGCGCCCCAGAAGCTCTCCTCCTTCAAGCTCCGCCGGC
TGCAGAGCCAGTCCGTCGCCGCCGACGACGACCTCGACCACATCCCGGTCTGATCCA

PjLsi2

>PjLsi2-K1698-PI314521-highSi-genomic

ATGGCGCTCGCGTCTCTCCCCAAGGTGGTGCTGGGCTCCTTCGCCTTCGCCATCTTCTGG
ATGATGGCGGTGTTCCCGTCGGTGCCCTTCCTGCCCATCGGCCGGACGGCGGGGTCGCTG
CTCTCCGCCGTGCTCATGATCGTCTTCCACGTGATCAGCCCCGACGACGCCTACGCCTCC
ATCGACCTCCCCATCCTCGGCCTGCTCTTCTCCACCATGGTCGTCGGCGGCTACCTCAAG
AACGCCGGCATGTTCAAGCACCTGGGCACGCTCCTGGCCTGGAAGAGCCAGGGCGGGCGC
GACCTGCTCTGCCGCGTATGCGTCGTCACCGCGCTGGCCTCGGCGCTCTTCACCAACGAC
ACCTGCTGCGTCGTGCTCACCGAGTTCGTGCTCGAGCTCGCCGCTGAGCGGAACCTCCCG
GCCAAGCCCTTCCTCCTCGCGCTCGCCTCCAGCGCCAACATCGGCTCCAGTGCCACCCCC

ATCGGCAACCCGCAGAACCTGGTCATCGCCTTCAACAGCAAGATCTCCTTCCCCAAGTTT
TTGATCGGCATCCTGCCGGCCATGCTCGCCGGCATGGCCGTCAACATGGTCATGCTGCTC
TGCATGTACTGGAAGGACCTTGAGGGCGTCGCCCCCGACGCGGCCGGCAAGCAGATGACG
GTCGTGGAGGAGGGCCGCTCGCCATCCGTGGCATCGCACAAGAGCCCGCACCCGTTCAAC
GGCACCGTGGCCGACGACGGGGCCGAGTCAATGATGGAGGAGAACATCTCGACCAAGCAC
CCATGGTTCATGCAGTGCACGGAGCACCGGCGCAAGCTGTTCCTCAAGAGCTTCGCCTAC
ATCGTGACGGTGGGCATGGTGGTGGCCTACATGGCTGGGCTCAACATGTCGTGGACGGCC
ATCACCACCGCCATCGCGCTCGTCGTCGTCGACTTCCGGGACGCCGAGCCCTGCCTCGTC
AAGGTCTCCTACTCGCTGCTCGTCTTCTTCTCCGGCATGTTCATCACGGTGAGTGGGTTC
AACAAGACGGGGCTGCCGAGCGCCATCTGGAACTTCATGGCGCCCTACTCCAAGGTCGAC
AGCGCCGGCGGCATCTCCGTGCTCTCCGTCATCATCCTCCTCCTCTCCAACCTCGCATCC
AACGTACCCACAGGTATTCATACGCATGCATGCATGCATGGATTTTGTACCTCGGTACTA
TATATGACAGGAGCTAGTTAATTTGTCACATATGAACATATTATTCTGGTAAGAGTGACA
CCGTGACAGGACAAAGCACTCAAAGGAATAGAAAATATCTCTGCATATTCCAAACTAAAC
AAAGTACAAATTAAGTAAGCATTAATAATGTTGGTGCGGCTGCAGTTAGCACACGCAGGC
ATGCATGATGCATGGTTTTCTTTCCGAATACAACAAGCCAATTTTAAAGTATGAAACGGA
CCAAAATTGCTATCTAGTCATCACTCACTCACTAGACTCTAGCTAACTTAATAATTTGTT
ATTTTTCAAGCTTAATAATCGCTTACAATACTAAATATCACTACTAATATTATAGAGTTG
TAAAAATGTAACCCTTGGCCCCAATAGATCTCTGCCGGTGTAAGTGAATGACGTCATGTG
TTTTTGGTATGTGCAGTGCTGCTGATGGGGAACGAGGTGGCCACCGCGGCGGCGCTGATC
TCGCCGGCAGCGGTGACGCGGTCGTGGCTGCTGCTAGCGTGGGTGAGCACGGTGGCGGGC
AACCTGTCGCTGCTGGGGTCGGCGGCCAACCTGATCGTGTGCGAGCAGGCGCGCCGGGCG
CCGCGGAACGCCTACGAGCTCACCTTCTGGAACCACCTCGTCTTTGGCGTGCCCTCCACC
CTCATCGTCACCGCCGTCGGCATCCCCCTCATCGGAAAGATGTAGCCGCCTTGTCTAAGC

>PjLsi2-K46752-p0241-lowSi-genomic

ATGGCGCTCGCGTCTCTCCCCAAGGTGGTGCTGGGCTCCTTCGCCTTCGCCATCTTCTGG
ATGATGGCGGTGTTCCCGTCGGTGCCCTTCCTGCCCATCGGCCGGACGGCGGGGTCGCTG
CTCTCCGCCGTGCTCATGATCGTCTTCCACGTGATCAGCCCCGACGACGCCTACGCCTCC
ATCGACCTCCCCATCCTCGGCCTGCTCTTCTCCACCATGGTCGTCGGCGGCTACCTCAAG
AACGCCGGCATGTTCAAGCACCTGGGCACGCTCCTGGCCTGGAAGAGCCAGGGCGGGCGC
GACCTGCTCTGCCGCGTATGCGTCGTCACCGCGCTGGCCTCGGCGCTCTTCACCAACGAC
ACCTGCTGCGTCGTGCTCACCGAGTTCGTGCTCGAGCTCGCCGCTGAGCGGAACCTCCCG
GCCAAGCCCTTCCTCCTCGCGCTCGCCTCCAGCGCCAACATCGGCTCCAGTGCCACCCCC
ATCGGCAACCCGCAGAATCTGGTCATCGCCTTCAACAGCAAGATCTCCTTCCCCAAGTTT
TTGATCGGCATCCTGCCGGCCATGCTCGCCGGCATGGCCGTCAACATGGTCATGCTGCTC
TGCATGTACTGGAAGGACCTTGAGGGCGTCGCCCCCGACGCGGCCGGCAAGCAGATGACG
GTCGTGGAGGAGGGCCGCTCGCCATCCGTGGCATCGCACAAGAGCCCGCACCCGTTCAAC
GGCACCGTGGCCGACGACGGGGCCGAGTCAATGATGGAGGAGAACATCTCGACCAAGCAC
CCATGGTTCATGCAGTGCACGGAGCACCGGCGCAAGCTGTTCCTCAAGAGCTTCGCCTAC
ATCGTGACGGTGGGCATGGTGGTGGCCTACATGGCCGGGCTCAACATGTCGTGGACGGCC
ATCACCACCGCCATCGCGCTCGTCGTCGTCGACTTCCGGGACGCTGAGCCCTGCCTCGTC
AAGGTCTCCTACTCGCTGCTCGTCTTCTTCTCCGGCATGTTCATCACGGTGAGTGGGTTC
AACAAGACGGGGCTGCCGAGCGCCATCTGGAACTTCATGGCGCCCTACTCCAAGGTCGAC
AGCGCCGGCGGCATCTCCGTGCTCTCCGTCATCATCCTCCTCCTCTCCAACCTCGCATCC
AACGTACCCACAGGTATACATATGCATGCATGCATGCATGGATTTTGTACCTCGGTACTA
TATATGACAGGAGCTAGTTAATTTGTCACATATGAACATATTATTCTGGTAAGAGTGACA

CCGTGACAGGACAAAGCACTCAAAGGAATAGAAAATATCTCTGCATATTCCAAACTAAAC
AAAGTACAAATTAAGTAAGCATTAATAATGTTGGTGCGGCTGCAGTTAGCACACGCAGGC
ATGCATGATGCATGGTTTTCTTTCCGAATACAACAAGCCAATTTTAAAGTATGAAACGGA
CCAAAATTGCTATCTAGTCATCACTCACTCACTGCTTAATAATCGCTTACAATACTAAAT
ATCACTACTAATATTATAGAGTTGTAAAAATGTAACCCTTGGCCCCAATAGATCTCTGCC
GGTGTAAGTGAATGACGTCATGTGTTTTTGGTATGTGCAGTGCTGCTGATGGGGAACGAG
GTGGCCACCGCGGCGGCGCTGATCTCGCCGGCAGCGGTGACGCGGTCGTGGCTGCTGCTA
GCGTGGGTGAGCACGGTGGCGGGCAACCTGTCGCTGCTGGGGTCGGCGGCCAACCTGATC
GTGTGTGAGCAGGCGCGCCGGGCGCCGCGGAACGCCTACGAGCTCACCTTCTGGAACCAC
CTCGTCTTTGGCGTGCCCTCCACCCTCATCGTCACCGCCGTCGGCATCCCCCTCATCGGA
AAGATGTAGCCGCCTTGTCTAAGC

PjLsi3

>PjLsi3-K1698-PI314521-highSi-genomic
ATGGCGCTGGCGTCTCTGTCCAAGGTGGTGATGGGGTCGGCGGCGTTCGGGGTGTTCTGG
GTGCTGGCCGTGTTCCCGTCGGTGCCTTTCCTGCCGATCGGCCGCACGGCGGGTGCGCTG
CTGGGGGCTGCGCTGATGGTGGTCTTCCACGTGATCAGCCCGGATGACGCCTACGCCTCC
GTGGACCTCCCCATCCTGGGCCTCCTCTTTGCCACCATGGTCGTCGGCGGCTACCTCAAG
AGCGCCGGCATGTTCGGGCACCTGGGCCGGCTGCTGGCGTGGCGGAGCCAGGGCGGGCGC
GACCTCCTGTGCCGCGTCTGCGTCGTCACGGCCCTCGCCAGCGCGCTCTTCACCAACGAC
ACCTGCTGCGTCGGGCTCACCGAGTTCGTGCTCGAGCTCGCCGCCGAGCGCAACCTCCCC
GCGAAGCCGTTCCTGCTGGCGCTGGCCACCAGCGCCAACATCGGGTCCAGCGCCACGCCC
ATCGGGAACCCGCAGAACCTGGTCATCGCCTTCAACAGCAAGATCTCCTTCGTGGGCTTC
TTCCTCGGCATCCTGCCGGCCATGCTCGCGGGTATGGCCGTCAACATGGTCATGCTGCTC
TGCATGTACTGGAAGGACCTCGAGGGGAACAGTCCCGACGCGGTGGCCGCCGAGAAGGAG
ATGTCGGCCGTCGAGGAGGGTCGCCGGCCGTCCCCGGCAACGTCGACGACGCTCAAGAGC
CCCGCCCAGACGATGTTGGTCCAGTCGTCGTCGCTGGTCGCCGCCGCCGACCACGACTCG
GTGATGGCGGAGAGCATCTCGACCAAGCACCGGTGGTTCATACAGTGCTCGGCGCCGCAG
CGGAGGCTGTTCCTCAAGAGCTTCGCGTACATCGTGACCGCCGGCATGCTGGTCGCCTAC
ATGCTGGGGCTCAACATGTCGTGGACCGCCATCACCACCGCCGTGGCCCTCATCGTCGTG
GACTTCCGCGACGCCGAGCTCTGCCTCGGCAAGGTGTCTTACTCGCTGCTGGTTTTCTTC
ACGGGGATGTTCGTGACGGTGAGCGGGTTCAATAACACGGGGCTCCCTGGCGCCATCTGG
AACGTCGTGGC**GCCATACTCCAAGATCAACCAC**GTCAGCGGCGTCACCGTGCTCTCCCTC
ATCATCCTCCTCCTCTCCAACCTTGCCTCCAACGTACCAACCGGTTAGTGAATTCTTACA
TCAAGTGTACGTACTATCACTTGTGCAGTTGTGCTAGCTGTTGTGCATGCACGTAAAATA
TAGTACTATTTGTGTAGATCTTTCTTGTTACGGCATAGTACCGGAATAATTGTTTAGTGG
CTAGCTAAGGGCTATGGAAGTACTGCCTGTTTTGTTCGTGTGTGCCGCTGACGCATTAAG
CAATTTGTATGCCGTACATGCATGCCGTGCGTGCGTGCATGACAACTGACAAGACGGCTT
GAAAAAATGTTTGGCAATTAACTGACACGAGGATGCGTGTGATTCATGCAGTGCTGCTGA
TGGGTGACGAGGTGGCGGCGTCGGCAGCGACCATCTCAGCGGCGGCGGTGACGCGGTCGT
GGCTGCTGCTGGCGTGGGTGAGCACGGTGGCGGGTAACCTGTCGCTGCTGGGGTCGGCGG
CGAACCTCATCGTGTGCGAGCAGGCACGCCGAGCGCCGCGCAACGCACACGACCTCACGT
TCTGGAGCCACGTCGTCTTCGGCGCACCGTCCACGCTCGTCGTCACCGCCGTCGGCATAC
CCCTCATCGGCATCATCAACGCTGCCTGAATC

>PjLsi3-K46752-p0241-lowSi-genomic

ATGGCGCTGGCGTCTCTGTCCAAGGTGGTGATGGGGTCGGCGGCGTTCGGCGTGTTCTGG
GTTCTGGCCGTGTTCCCGTCGGTGCCTTTCCTGCCGATCGGCCGCACGGCGGGTGCGCTG
CTGGGGGCTGCGCTGATGGTCGTCTTCCACGTGATCAGCCCGGATGACGCCTACGCCTCC
GTGGACCTCCCCATCCTGGGCCTCCTCTTTGCCACCATGGTCGTCGGCGGCTACCTCAAG
AGCGCCGGCATGTTCGGGCACCTGGGCCGGCTGCTGGCGTGGCGGAGCCAGGGCGGGCGC
GACCTCCTGTGCCGCGTCTGCGTCGTCACGGCCCTCGCCAGCGCGCTCTTCACCAACGAC
ACCTGCTGCGTCGTGCTCACGGAGTTCGTGCTCGAGCTCGCCGCCGAGCGCAACCTCCCC
GCGAAGCCGTTCCTGCTGGCGCTGGCCACCAGCGCCAACATCGGGTCCAGCGCCACGCCC
ATCGGGAACCCGCAGAATCTGGTCATCGCTTTCAACAGCAAGATCTCCTTCGTGGGCTTC
TTCCTCGGCATCCTGCCGGCCATGCTCGCGGGTATGGCCGTCAACATGGTCATGCTGCTC
TGCATGTACTGGAAGGACCTCGAGGGGAACAGTCCCGACGCGGTGGCCGCCGAGAAGGAG
ATGGCGGCCGTCGAGGAGGGTCGCCGGCCGTCCCCGGAAACGTCGACGACGCTCAAGAGC
CCCGCCCAGACGATGTTGGTCCAGTCGTCGTCGCTGGTCGCCGCCGCCAACCACGACTCG
GTGATGGCGGAGAGCATCTCGACCAAGCACCGGTGGTTCATACAGTGCTCGGCGCCGCAG
CGGAGGCTGTTCCTCAAGAGCTTCGCGTACATCGTGACCGCCGGCATGCTGGTCGCCTAC
ATGCTGGGGCTCAACATGTCGTGGACCGCCATCACCACCGCCGTGGCCCTTATCGTCGTG
GACTTCCGCGACGCCGAGCTCTGCCTCGGCAAGGTGTCTTACTCGCTGCTGGTTTTCTTC
ACGGGGATGTTCGTGACGGTGAGCGGGTTCAATAAGACGGGGCTCCCTGGCGCCATCTGG
AACGTCATGGC**GCCATACTCCAAGATCAACCAC**GTCAGCGGCGTCACCGTGCTCTCCCTC
ATCATCCTCCTCCTCTCCAACCTTGCCTCCAACGTACCAACCGGTTAGTGAACTCTTACA
TCAAGTGTACGTATCACTTGTGCTAGCTGTTGTGCATGCACGTAAAATCTACTGTACTAT
ACTATTTGTGTAGATCTTTCTTGTTACGGCATAGTACCGGAACAATTGTTTAGTGGCTAG
CTAAGGGCTATGGAAAGTACTGCCTGTTTTGTTCGGGTGTGCCGCTGACGCATTAAGCAA
TTTGTATGCCGTGCGTGCATGACAACTGACAAGACGGCTTGAAAAAATTGTTGGCAATTA
ACTGACACGAGGATGCGTGTGGTTCATGCAGTGCTGCTGATGGGTGACGAGGTGGCGGCG
TCGGCAGCGACCATTTCAGCGGCGGCGGTGACGCGGTCGTGGCTGCTGCTGGCGTGGGTG
AGCACGGTGGCGGGTAACCTGTCCCTGCTGGGGTCGGCGGCGAACCTCATCGTGTGCGAG
CAGGCGCGCCGGGCGCCCGCGCAACGCGCATGACCTCACCTTCTGGAGCCACGTCGTCTTC
GGCGCACCGTCCACGCTCGTCGTCACCGCCGTCGGCATACCCCTCATCGGCATCATCAAC
GCTGCCTGAATC

Supplementary material S8

Statistical analysis of Kruskal–Wallis test presented in Table 3. Results of statistical treatments to analyse the differences between genotype groups (*aa*, *ab* and *bb*) with corresponding Si accumulation for each of studied *PjLsi* gene

PjLsi1

C13 =CHIDIST(C11,SUM(X19:AQ19)-1)

	A	B	C	D
1	This spreadsheet performs the **Kruskal-Wallis test**. If you have just two groups, this is equivalent to a Mann-Whitney U-test.			
2	It will handle up to 20 groups with up to 1000 observations in each group.			
3	For more information, see http://www.biostathandbook.com/kruskalwallis.html			
4	The spreadsheet comes with the oyster Fst data from that web page entered as an example.			
5	To use it, replace the oyster data with your numbers.			
6	"Adjusted H" is the test statistic, including an adjustment for the number of ties.			
7				
8				
11		**adjusted H:**	**59.819**	
12		**d.f.:**	**2**	
13		**P value:**	**1.02E-13**	
14				
15				
20	group names-->	aa	ab	bb
21	data-->	4.11	2.65	2.35
22		3.79	2.61	2.34
23		3.72	**2.59**	2.30
24		3.55	2.57	2.30
25		3.42	2.56	2.26
26		3.40	2.54	2.24
27		3.34	2.53	2.22
28		3.29	2.48	2.20
29		3.26	2.46	2.18
30		3.15	2.44	2.09
31		3.13	2.44	2.09
32		3.00	2.44	2.06
33		2.93	2.44	1.86
34		2.92	2.43	1.86
35		2.92	2.40	1.85
36		2.92	2.39	1.73
37		2.91	2.36	1.59
38		2.85	2.36	1.46
39		2.84	2.31	1.45
40		2.80	2.29	
41		2.79		
42		2.74		
43		2.74		
44		2.69		
45		2.68		
46		2.66		
47		2.66		
48		**2.66**		
49		2.65		
50		2.62		
51		2.61		
52		2.59		
53		2.58		

Sheet1

PjLsi2

C13 =CHIDIST(C11,SUM(X19:AQ19)-1)

This spreadsheet performs the **Kruskal-Wallis test**. If you have just two groups, this is equivalent to a Mann-Whitney U-test.
It will handle up to 20 groups with up to 1000 observations in each group.
For more information, see http://www.biostathandbook.com/kruskalwallis.html
The spreadsheet comes with the oyster Fst data from that web page entered as an example.
To use it, replace the oyster data with your numbers.
"Adjusted H" is the test statistic, including an adjustment for the number of ties.

**adjusted H:**	**57.196**
**d.f.:**	**2**
**P value:**	**3.80E-13**

group names-->	aa	ab	bb
data-->	4.11	**2.66**	2.39
	3.79	2.65	2.36
	3.72	2.62	2.36
	3.55	2.59	2.34
	3.42	2.56	2.31
	3.40	2.54	2.30
	3.34	2.53	2.29
	3.29	2.48	2.22
	3.26	2.46	2.20
	3.15	2.44	2.18
	3.13	2.44	2.09
	3.00	2.44	2.09
	2.93	2.44	2.06
	2.92	2.43	1.86
	2.92	2.40	1.86
	2.92	2.35	1.85
	2.91	2.30	1.73
	2.85	2.26	1.59
	2.84	2.24	1.46
	2.80		1.45
	2.79		
	2.74		
	2.74		
	2.69		
	2.68		
	2.66		
	2.66		
	2.65		
	2.61		
	2.61		
	**2.59**		
	2.58		
	2.57		

Sheet1

PjLsi3

C13 =CHIDIST(C11,SUM(X19:AQ19)-1)

This spreadsheet performs the **Kruskal-Wallis test**. If you have just two groups, this is equivalent to a Mann-Whitney U-test.
It will handle up to 20 groups with up to 1000 observations in each group.
For more information, see http://www.biostathandbook.com/kruskalwallis.html
The spreadsheet comes with the oyster Fst data from that web page entered as an example.
To use it, replace the oyster data with your numbers.
"Adjusted H" is the test statistic, including an adjustment for the number of ties.

**adjusted H:**	**59.172**
**d.f.:**	**2**
**P value:**	**1.42E-13**

group names-->	aa	ab	bb
data-->	4.11	2.66	2.36
	3.79	2.65	2.34
	3.72	2.61	2.30
	3.55	**2.59**	2.30
	3.42	2.57	2.29
	3.40	2.56	2.24
	3.34	2.54	2.22
	3.29	2.53	2.20
	3.26	2.48	2.18
	3.15	2.46	2.09
	3.13	2.44	2.09
	3.00	2.44	2.06
	2.93	2.44	1.86
	2.92	2.44	1.86
	2.92	2.43	1.85
	2.92	2.40	1.73
	2.91	2.39	1.59
	2.85	2.36	1.46
	2.84	2.35	1.45
	2.80	2.31	
	2.79	2.26	
	2.74		
	2.74		
	2.69		
	2.68		
	2.66		
	**2.66**		
	2.65		
	2.62		
	2.61		
	2.59		
	2.58		

Sheet1
